# Genetic variants in the TGFβ-signaling pathway influence expression of miRNAs in colon and rectal normal mucosa and tumor tissue

**DOI:** 10.18632/oncotarget.14508

**Published:** 2017-01-05

**Authors:** Martha L. Slattery, Andromahi Trivellas, Andrew J. Pellatt, Lila E. Mullany, John R. Stevens, Roger K. Wolff, Jennifer S. Herrick

**Affiliations:** ^1^ Department of Medicine, University of Utah, Salt Lake City, Utah, USA; ^2^ Tulane Medical School, New Orleans, Louisiana, USA; ^3^ Department of Mathematics and Statistics, Utah State University, Logan, Utah, USA

**Keywords:** colorectal cancer, miRNA, TGF-β, eIF4E, SMAD

## Abstract

The TGF-β signaling pathway is involved in regulation of cell growth, angiogenesis, and metastasis. We test the hypothesis that genetic variation in the TGF-β signaling pathway alters miRNA expression.

We use data from 1188 colorectal cancer cases to evaluate associations between 80 SNPs in 21 genes.

Seven variants *eIF4E* rs12498533, *NFκB1* rs230510, *TGFB1* rs4803455, *TGFBR1* rs1571590 and rs6478974, *SMAD3* rs3743343, and *RUNX1* rs8134179 were associated with expression level of miRNAs in normal colorectal mucosa. *RUNX2* rs12333172 and *BMPR1B* rs13134042 were associated with miRNAs in normal colon mucosa; *eIF4EBP3* rs250425, *SMAD3* rs12904944, *SMAD7* rs3736242, and *PTEN* rs532678 were associated with miRNA expression in normal rectal mucosa. Evaluation of the differential expression between carcinoma and normal mucosa showed that *SMAD3* rs12708491 and rs2414937, *NFκB1* rs230510 and rs3821958, and RUNX3 rs6672420 were associated with several miRNAs for colorectal carcinoma. Evaluation of site-specific differential miRNA expression showed that *BMPR1B* rs2120834, *BMPR2* rs2228545, and *eIF4EBP3* rs250425 were associated with differential miRNA expression in colon tissue and *SMAD3* rs12901071, rs1498506, and rs2414937, *BMPR2* rs2228545, and *RUNX2* rs2819854, altered differential miRNA expression in rectal tissue.

These data support the importance of the TGF-β signaling pathway to the carcinogenic process, possibly through their influence on miRNA expression levels.

## INTRODUCTION

The TGF-β signaling pathway is an essential regulator of cellular proliferation, differentiation, apoptosis, extracellular matrix remodeling in the cell, and is involved in angiogenesis and inflammation [1, 2]. Components of this pathway have been associated with colorectal cancer risk and survival [3–8]. There are several components of the TGF-β signaling pathway. Smads are key intracellular mediators of the transcriptional responses to TGF-β [9]. Bone morphogenetic proteins (BMP), trigger a Smad-signaling cascade that is linked to reduced cell proliferation and cellular growth kinetics of glioblastomas [10, 11]. The Runt-related transcription factors (RUNX), *RUNX1*, *RUNX2*, and *RUNX3* [12] are involved in signaling cascades mediated by TGF-β and BMP [13–16]. All three of the RUNX genes have been shown to bind Smads [17–19]. Studies in *RUNX3* knockout mice have shown defects in apoptotic response to TGF-β; *RUNX2* transgenic mice were hypersensitive to TGF-β in one study [14]. Mitogen-activated protein kinase 1 (MAPK1) activates RUNX2 [20] and is involved in the TGF-β-signaling pathway through its role in Smad signaling [21, 22]. Eukaryotic translation initiation factor 4E (eIF4E) is a translational regulator; expression of eIF4E in human colon cancer cells promotes the TGFβ stimulation of adhesion molecules [23]. Other genes, such as nuclear factor kappa B1 (NFκB1), mammalian target of rapamycin (mTOR), and phosphatase tensin homolog deleted on chromosome 10 (PTEN), are major regulators of inflammation, are associated with colorectal cancer, and influence the TGF-B signaling pathway [24–26].

The TGF-β signaling pathway has been shown to suppress growth and tumorigenicity in a normal cellular environment; but, in neoplastic cells, its role converts to the promotion of invasion and metastasis [27]. It has recently been hypothesized that miRNAs may be intermediaries in a TGFβ-miRNA pathway that both mediates cell growth and promotes invasive behavior [28]. Some studies have linked miRNAs and the TGF-β signaling pathway. For instance miR-181a has its expression upregulated by TGFβ, which in turn promotes breast cancer metastasis [28, 29]. Other studies have shown that TGFβ activates AKT kinase through mechanisms involving miRNAs, and that TGFβ-signaling may be involved in the control of miRNA biogenesis [30, 31].

In this paper, we test the hypothesis that genetic variation in the TGF-β signaling pathway is associated with miRNA expression level. We use data from a large study of colorectal cancer cases. We focus on genetic variants in 21 genes in the TGF-β signaling pathway that we have previously shown to be associated with either colon or rectal cancer risk or survival after diagnosis. Our ability to show that these genetic variants alter miRNA expression adds support to the importance of these SNPs in cancer risk.

## RESULTS

The majority of cases for both colon and rectal cancer studies were male (Table 1). The mean age at diagnosis was slightly older for those individuals enrolled in the colon cancer study compared to those enrolled in the rectal cancer study. Over 80% of the study population was non-Hispanic white. The majority of cases were diagnosed at either an AJCC stage 1 or stage 2.

**Table 1:** Description of study population

	Colon		Rectal	
	N	%	N	%
Center				
Kaiser	509	73.9	302	60.5
Utah	180	26.1	197	39.5
Sex				
Male	383	55.6	296	59.3
Female	306	44.4	203	40.7
Race				
White, non-Hispanic	594	86.2	400	80.2
Hispanic	47	6.8	46	9.2
Black, non-Hispanic	46	6.7	18	3.6
Other/Unknown	2	0.3	35	7.0
Vital Status^1^				
Dead	279	40.6	184	36.9
Alive	409	59.4	315	63.1
AJCC Stage^2^				
1	169	24.8	227	46.0
2	221	32.4	94	19.0
3	226	33.1	142	28.7
4	66	9.7	31	6.3
	Mean	SD	Mean	SD
Age	65.3	9.1	61.8	11.1
Survival Months	69.3	32.7	67.6	27.2

^1^ Vital Status missing for one individual.

^2^ AJCC stage unknown for 12 people.

MiRNA expression level differed across genotypes of seven SNPs, *eIF4E* rs12498533, *NFκB1* rs230510, *TGFB1* rs4803455, *TGFBR1* rs1571590 and rs6478974, *SMAD3* rs3743343, and *RUNX1* rs8134179 in normal colorectal mucosa (Table 2). All but four miRNAs, miR-4324, miR-4655-3p, miR-3663-3p, and miR-1539 that were significantly different by both *TGFBR1* rs1571590 and *TGFBR1* rs6478974 when considering an FDR of <0.09 (Supplementary Table 1 for all associations). However, the majority of miRNAs associated with rs6478974 were downregulated for the variant allele, while for rs1571590 the miRNAs were equally up and downregulated for the variant allele. For *EIF4E* rs12498533, *NFKB1* rs230510, *SMAD3* rs3743343, *TGFB1* rs4803455, all miRNAs were downregulated in the presence of the variant allele. However, for *RUNX1* rs8134179 all of the miRNAs were upregulated in the variant allele.

**Table 2:** Associations between TGFB-pathway SNPs and miRNA differential expression in colorectal tissue

miRNA	Mean	Mean	P-values	FDR adjusted P	Direction
***EIF4E*** (rs12498533)	AA (N=342)	AC/CC (N=798)			
hsa-miR-221-3p	3.21	2.25	<.0001	0.0820	Downregulated
***TGFBR1*** (rs1571590)^1^	AA (N=738)	AG/GG (N=372)			
hsa-miR-100-5p	7.83	6.06	<.0001	0.0373	Downregulated
hsa-miR-130a-3p	3.20	2.26	0.0004	0.0373	Downregulated
hsa-miR-143-3p	7.23	5.89	0.0004	0.0373	Downregulated
hsa-miR-19b-3p	8.53	6.67	0.0003	0.0373	Downregulated
hsa-miR-29c-3p	15.56	13.19	0.0005	0.0373	Downregulated
hsa-miR-3666	19.31	20.74	<.0001	0.0373	Upregulated
hsa-miR-4324	1.30	0.98	0.0002	0.0373	Downregulated
hsa-miR-4655-3p	22.54	24.35	0.0005	0.0373	Upregulated
hsa-miR-4659a-3p	27.57	29.81	0.0003	0.0373	Upregulated
hsa-miR-5003-3p	18.59	20.12	0.0005	0.0373	Upregulated
hsa-miR-6500-5p	18.18	19.53	0.0005	0.0373	Upregulated
hsa-miR-662	41.11	43.73	0.0006	0.0410	Upregulated
hsa-miR-140-3p	7.16	6.09	0.0008	0.0492	Downregulated
hsa-miR-26a-5p	86.12	75.56	0.0009	0.0492	Downregulated
hsa-miR-4294	21.48	22.41	0.0009	0.0492	Upregulated
***NFKB1*** (rs230510)	AA (N=387)	AT/TT (N=723)			
hsa-miR-4446-3p	16.16	13.90	<.0001	0.0820	Downregulated
***SMAD3*** (rs3743343)^1^	TT/TC (N=1035)	CC (N=75)			
hsa-miR-1285-3p	19.98	17.98	<.0001	0.0410	Downregulated
hsa-miR-3935	16.98	14.78	<.0001	0.0410	Downregulated
***TGFB1*** (rs4803455)	CC (N=275)	CA/AA (N=881)			
hsa-miR-146b-5p	1.48	0.92	0.0002	0.0820	Downregulated
hsa-miR-3609	1.68	1.20	<.0001	0.0820	Downregulated
***TGFBR1*** (rs6478974)^1^	TT (N=359)	TA/AA (N=750)			
hsa-miR-1226-5p	54.62	51.50	0.0011	0.0355	Downregulated
hsa-miR-151a-5p	7.57	9.03	0.0011	0.0355	Upregulated
hsa-miR-1587	1405.23	1292.11	0.0013	0.0355	Downregulated
hsa-miR-199a-3p	16.51	19.29	0.0005	0.0355	Upregulated
hsa-miR-200a-3p	18.50	20.89	0.0006	0.0355	Upregulated
hsa-miR-20a-5p	13.21	15.67	0.0016	0.0355	Upregulated
hsa-miR-214-3p	4.76	5.65	0.0005	0.0355	Upregulated
hsa-miR-222-3p	8.28	8.99	0.0016	0.0355	Upregulated
hsa-miR-22-3p	12.78	14.57	0.0007	0.0355	Upregulated
hsa-miR-25-3p	8.70	10.46	0.0016	0.0355	Upregulated
hsa-miR-26b-5p	15.33	17.83	0.0004	0.0355	Upregulated
hsa-miR-30c-5p	7.73	9.17	0.0012	0.0355	Upregulated
hsa-miR-3156-5p	95.91	90.85	0.0009	0.0355	Downregulated
hsa-miR-3185	17.99	16.77	0.0005	0.0355	Downregulated
hsa-miR-33b-3p	19.25	15.53	0.0006	0.0355	Downregulated
hsa-miR-3620-5p	95.85	89.60	0.0015	0.0355	Downregulated
hsa-miR-3621	38.13	35.90	0.0015	0.0355	Downregulated
hsa-miR-370	41.68	39.44	0.0002	0.0355	Downregulated
hsa-miR-3917	99.12	93.52	0.0005	0.0355	Downregulated
hsa-miR-3934-3p	29.59	28.27	0.0014	0.0355	Downregulated
hsa-miR-3937	70.06	65.67	0.0013	0.0355	Downregulated
hsa-miR-4253	46.35	43.92	0.0012	0.0355	Downregulated
hsa-miR-4294	22.41	21.50	0.0011	0.0355	Downregulated
hsa-miR-4535	70.15	66.72	0.0007	0.0355	Downregulated
hsa-miR-4655-5p	34.25	32.23	0.0013	0.0355	Downregulated
hsa-miR-4695-5p	385.66	365.84	0.0009	0.0355	Downregulated
hsa-miR-4726-5p	22.13	21.13	0.0015	0.0355	Downregulated
hsa-miR-4734	210.70	198.06	0.0013	0.0355	Downregulated
hsa-miR-5196-5p	63.33	58.28	0.0012	0.0355	Downregulated
hsa-miR-550a-3-5p	28.29	26.77	0.0009	0.0355	Downregulated
hsa-miR-564	67.17	64.76	0.0014	0.0355	Downregulated
hsa-miR-601	46.91	44.06	0.0010	0.0355	Downregulated
hsa-miR-6084	18.25	16.81	0.0012	0.0355	Downregulated
hsa-miR-610	25.93	24.45	0.0013	0.0355	Downregulated
hsa-miR-6511b-5p	65.88	60.79	0.0005	0.0355	Downregulated
hsa-miR-760	34.80	32.70	0.0014	0.0355	Downregulated
hsa-miR-93-5p	10.92	12.98	<.0001	0.0355	Upregulated
hsa-miR-15b-5p	20.02	23.05	0.0019	0.0359	Upregulated
hsa-miR-3187-3p	9.99	9.16	0.0021	0.0359	Downregulated
hsa-miR-4646-5p	112.80	106.87	0.0021	0.0359	Downregulated
hsa-miR-4738-3p	50.24	48.07	0.0020	0.0359	Downregulated
hsa-miR-4740-5p	33.39	31.96	0.0017	0.0359	Downregulated
hsa-miR-5189	33.40	31.42	0.0021	0.0359	Downregulated
hsa-miR-550b-2-5p	38.12	35.90	0.0020	0.0359	Downregulated
hsa-miR-6124	522.71	487.27	0.0018	0.0359	Downregulated
hsa-miR-6165	323.94	306.32	0.0021	0.0359	Downregulated
hsa-miR-6500-5p	19.30	18.32	0.0019	0.0359	Downregulated
hsa-miR-671-5p	291.07	271.09	0.0018	0.0359	Downregulated
hsa-miR-6722-3p	103.52	97.67	0.0022	0.0368	Downregulated
hsa-miR-196b-5p	3.53	4.76	0.0023	0.0370	Upregulated
hsa-miR-4481	84.65	78.48	0.0023	0.0370	Downregulated
hsa-miR-4758-5p	131.05	120.75	0.0024	0.0378	Downregulated
hsa-miR-1247-3p	26.28	25.11	0.0028	0.0396	Downregulated
hsa-miR-199a-5p	6.22	7.34	0.0027	0.0396	Upregulated
hsa-miR-345-3p	59.74	56.13	0.0026	0.0396	Downregulated
hsa-miR-3622b-5p	38.67	36.86	0.0028	0.0396	Downregulated
hsa-miR-451a	25.98	32.37	0.0029	0.0396	Upregulated
hsa-miR-5001-5p	1180.01	1096.07	0.0028	0.0396	Downregulated
hsa-miR-6511a-5p	43.38	40.66	0.0029	0.0396	Downregulated
hsa-miR-6724-5p	886.55	827.04	0.0027	0.0396	Downregulated
hsa-miR-28-5p	0.65	0.97	0.0030	0.0397	Upregulated
hsa-miR-30b-5p	17.20	19.78	0.0030	0.0397	Upregulated
***RUNX1*** (rs8134179)^1^	TT (N=793)	TC/CC (N=317)			
hsa-miR-138-2-3p	6.29	7.08	0.0003	0.0469	Upregulated
hsa-miR-3177-5p	4.23	4.85	0.0004	0.0469	Upregulated
hsa-miR-3614-5p	4.96	5.95	<.0001	0.0469	Upregulated
hsa-miR-4461	3.36	4.14	0.0003	0.0469	Upregulated
hsa-miR-4519	7.77	8.67	0.0003	0.0469	Upregulated
hsa-miR-5696	6.03	7.10	0.0004	0.0469	Upregulated
hsa-miR-658	0.64	0.80	0.0002	0.0469	Upregulated

^1^Data presented is restricted to those with the lowest adjusted p values. Supplemental Table 1 contains all associations with FDR <0.09.

Four SNPs, *RUNX2* rs12333172, *eIF4E* rs12498533, *BMPR1B* rs13134042, and *RUNX1* rs8134179 were associated predominately with increased miRNA expression in normal colonic mucosa for the variant allele. Six SNPs were associated with miRNA expression in normal rectal mucosa: *SMAD3* rs12904944, *TGFBR1* rs1571590, *eIF4EBP3* rs250425, *SMAD7* rs3736242, and *PTEN* rs532678, and *TGFBR1* rs6478974 (Table 3). SNPs in *TGFBR1* were associated with expression of miRNAs for colorectal normal mucosa overall as well as for rectal normal mucosa specifically. *eIF4E* rs12498533 was associated with downregulation of miR-221-3p in overall colorectal mucosa and also was associated with upregulation of miR-3180-3p in normal colonic mucosa. *RUNX1* rs8134179 was associated with miR-658 in a similar manner for colorectal (Supplemental Table 2 For all miRNAs associated with TGFBeta signaling pathway SNPs in normal colonic mucosa and Supplemental Table 3 for associations in normal rectal mucosa) normal mucosa overall and colon-specific normal mucosa.

**Table 3:** Associations between SNPs in TGFβ-signaling pathway and miRNA expression in site-specific normal mucosa

Colon Tissue					
miRNA	Mean	Mean	P-values	FDR adjusted P	Direction
***RUNX2*** (rs12333172)^1^	CC/CT (N=626)	TT (N=36)			
hsa-miR-1226-5p	54.59	79.49	0.0015	0.0717	Upregulated
hsa-miR-26a-5p	82.50	64.46	0.0009	0.0717	Downregulated
hsa-miR-30c-1-3p	11.15	15.53	0.0012	0.0717	Upregulated
hsa-miR-3621	37.96	53.40	0.0014	0.0717	Upregulated
hsa-miR-3663-3p	167.22	252.07	0.0015	0.0717	Upregulated
hsa-miR-3960	10454.46	20170.24	0.0015	0.0717	Upregulated
hsa-miR-4316	6.93	4.87	0.0014	0.0717	Downregulated
hsa-miR-4498	63.88	56.98	0.0002	0.0717	Downregulated
hsa-miR-4516	17558.75	31136.08	0.0015	0.0717	Upregulated
hsa-miR-4634	326.83	612.64	0.0013	0.0717	Upregulated
hsa-miR-4701-3p	75.06	109.36	0.0003	0.0717	Upregulated
hsa-miR-4783-3p	16.70	23.43	0.0013	0.0717	Upregulated
hsa-miR-5739	1304.71	1856.95	0.0012	0.0717	Upregulated
hsa-miR-628-3p	18.91	17.63	0.0010	0.0717	Downregulated
hsa-miR-6723-5p	135.57	197.55	0.0009	0.0717	Upregulated
hsa-miR-758-5p	32.86	30.14	0.0008	0.0717	Downregulated
hsa-miR-921	21.74	18.66	0.0012	0.0717	Downregulated
***EIF4E*** (rs12498533)	AA (N=188)	AC/CC (N=473)			
hsa-miR-3180-3p	16.08	18.65	<.0001	0.0813	Upregulated
***BMPR1B*** (rs13134042)	GG/GA (N=640)	AA (N=22)			
hsa-miR-10a-3p	3.40	6.55	<.0001	0.0813	Upregulated
***RUNX1*** (rs8134179)^1^	TT (N=463)	TC/CC (N=199)			
hsa-miR-526b-5p	3.52	4.13	<.0001	0.0406	Upregulated
hsa-miR-658	0.72	0.95	<.0001	0.0406	Upregulated
hsa-miR-4659b-3p	3.77	4.78	0.0002	0.0542	Upregulated
**Rectal Tissue**					
***SMAD3*** (rs12904944)	GG (N=193)	GA/AA (N=255)			
hsa-miR-662	38.47	42.35	<.0001	0.0825	Upregulated
***TGFBR1*** (rs1571590)	AA (N=301)	AG/GG (N=147)			
hsa-miR-29b-3p	7.59	6.19	0.0002	0.0825	Downregulated
hsa-miR-29c-3p	15.03	12.35	0.0002	0.0825	Downregulated
***EIF4EBP3*** (rs250425)	CC (N=288)	CT/TT (N=181)			
hsa-miR-4715-5p	1.79	2.68	<.0001	0.0825	Upregulated
***SMAD7*** (rs3736242)	GG/GA (N=423)	AA (N=25)			
hsa-miR-431-5p	37.73	29.85	<.0001	0.0825	Downregulated
PTEN (rs532678)^1^	CC (N=167)	CT/TT (N=281)			
hsa-miR-10a-3p	2.55	1.86	0.0002	0.0450	Downregulated
hsa-miR-1203	2.03	1.55	0.0014	0.0450	Downregulated
hsa-miR-1261	3.26	2.61	0.0012	0.0450	Downregulated
hsa-miR-130b-3p	3.22	2.47	0.0002	0.0450	Downregulated
hsa-miR-1323	3.74	2.91	0.0008	0.0450	Downregulated
hsa-miR-138-2-3p	5.85	4.80	0.0018	0.0450	Downregulated
hsa-miR-146b-5p	1.11	0.73	0.0008	0.0450	Downregulated
hsa-miR-3617-5p	3.28	2.63	0.0012	0.0450	Downregulated
hsa-miR-3660	3.56	2.73	0.0003	0.0450	Downregulated
hsa-miR-425-3p	13.76	16.55	0.0005	0.0450	Upregulated
hsa-miR-4433-5p	18.04	20.40	0.0010	0.0450	Upregulated
hsa-miR-4436a	4.67	3.67	0.0005	0.0450	Downregulated
hsa-miR-4436b-3p	6.46	5.40	0.0011	0.0450	Downregulated
hsa-miR-4448	8.25	7.07	0.0017	0.0450	Downregulated
hsa-miR-4508	51.88	57.14	0.0012	0.0450	Upregulated
hsa-miR-452-5p	8.70	7.25	0.0009	0.0450	Downregulated
hsa-miR-4657	1.60	1.16	0.0018	0.0450	Downregulated
hsa-miR-4665-3p	104.81	119.79	0.0012	0.0450	Upregulated
hsa-miR-4682	0.95	0.62	0.0018	0.0450	Downregulated
hsa-miR-4746-5p	2.91	2.33	0.0013	0.0450	Downregulated
hsa-miR-4748	4.64	3.78	0.0006	0.0450	Downregulated
hsa-miR-4787-3p	94.67	106.09	0.0003	0.0450	Upregulated
hsa-miR-500a-3p	2.43	1.95	0.0007	0.0450	Downregulated
hsa-miR-509-5p	7.57	6.81	0.0018	0.0450	Downregulated
hsa-miR-516b-5p	5.41	4.24	<.0001	0.0450	Downregulated
hsa-miR-518a-5p	4.58	3.86	0.0013	0.0450	Downregulated
hsa-miR-518c-5p	1.22	0.82	0.0014	0.0450	Downregulated
hsa-miR-519e-5p	1.93	1.32	0.0006	0.0450	Downregulated
hsa-miR-583	3.31	2.54	0.0018	0.0450	Downregulated
hsa-miR-629-3p	14.57	16.06	0.0015	0.0450	Upregulated
hsa-miR-659-5p	1.58	0.98	0.0008	0.0450	Downregulated
hsa-miR-766-3p	26.60	29.75	0.0017	0.0450	Upregulated
hsa-miR-92a-1-5p	0.50	0.33	0.0015	0.0450	Downregulated
***TGFBR1*** (rs6478974)	TT (N=150)	TA/AA (N=298)			
hsa-miR-3917	94.31	87.13	<.0001	0.0825	Downregulated

^1^ Data presented is restricted to those with the lowest adjusted p values. Supplementary Table 2 and 3 contain all associations with FDR <0.09.

Differences in expression between carcinoma and normal mucosa, or differential miRNA expression by genotype, was associated with six SNPs, *SMAD3* rs12708492, *BMPR2* rs228545, and *NFκB1* rs230510, SMAD3 rs2414937, *NFκB1* rs3821958, *and RUNX3* rs6672420 (Table 4) for colorectal cancer overall. *SMAD3* rs12708492 was associated with differential miRNA expression of six miRNAs miR-23a-5p, miR-30c-2-3p, miR-4286, miR-4753-5p, miR-4753-3p and miR-4679-5p by genotype. All miRNAs were downregulated with the variant allele except for miR-4286, where the expression was upregulated in the presence of the variant allele. *SMAD3* rs2414937 was associated with only one miRNA, miR-590-5p, and it was upregulated among those with the variant allele. *NFκB1* rs230510 was associated with expression of several miRNAs, all of which were downregulated for the variant allele, however the miRNAs associated with *NFκB1* rs3821958 were upregulated. Interestingly miR-4684-3p was upregulated in the variant allele of *NFκB1* rs230510 while downregulated in the presences of the variant allele of *NFκB1* rs3821958.

**Table 4:** Associations with SNPs in TGFβ-signaling pathway and differential expression of colorectal carcinoma and normal mucosa

miRNA	Mean	Mean	P-value	FDR adjusted P	Direction
***SMAD3*** (rs12708492)	CC (N=284)	CT/TT (N=778)			
hsa-miR-23a-5p	1.00	0.21	0.0002	0.0820	Downregulated
hsa-miR-30c-2-3p	0.57	-0.20	0.0003	0.0820	Downregulated
hsa-miR-4286	-198.94	-9.20	0.0005	0.0820	Upregulated
hsa-miR-4753-5p	3.88	2.04	0.0003	0.0820	Downregulated
hsa-miR-4755-3p	0.08	-1.16	0.0005	0.0820	Downregulated
hsa-miR-4769-5p	2.51	1.45	0.0006	0.0820	Downregulated
***BMPR2*** (rs2228545)	GG (N=1001)	GA/AA (N=65)			
hsa-miR-484	-0.01	0.79	<.0001	0.0820	Upregulated
**NFKB1** (rs230510)	AA (N=369)	AT/TT (N=697)			
hsa-miR-3187-5p	1.37	0.63	<.0001	0.0410	Downregulated
hsa-miR-3617-5p	0.38	-0.41	<.0001	0.0410	Downregulated
hsa-miR-4684-3p	0.12	-0.32	0.0002	0.0410	Downregulated
hsa-miR-6500-3p	0.44	-0.33	0.0002	0.0410	Downregulated
hsa-miR-493-3p	0.34	-0.17	0.0003	0.0492	Downregulated
hsa-miR-3189-5p	0.43	-0.34	0.0005	0.0586	Downregulated
hsa-miR-596	-0.01	-0.43	0.0005	0.0586	Downregulated
hsa-miR-4768-3p	-0.04	-0.68	0.0006	0.0615	Downregulated
hsa-miR-4461	0.94	0.31	0.0007	0.0638	Downregulated
hsa-miR-302c-5p	0.63	0.27	0.0008	0.0656	Downregulated
hsa-miR-4296	0.62	0.31	0.0009	0.0671	Downregulated
hsa-miR-4675	0.57	-0.22	0.0011	0.0752	Downregulated
hsa-miR-4251	1.52	0.93	0.0013	0.0761	Downregulated
hsa-miR-4458	-1.46	-2.15	0.0013	0.0761	Downregulated
hsa-miR-1276	0.34	-0.07	0.0014	0.0765	Downregulated
**SMAD3** (rs2414937)	GG/GC (N=1027)	CC (N=39)			
hsa-miR-590-5p	-0.14	1.65	<.0001	0.0820	Upregulated
***NFKB1*** (rs3821958)	AA (N=352)	AG/GG (N=714)			
hsa-miR-4282	-1.09	-0.30	<.0001	0.0410	Upregulated
hsa-miR-4684-3p	-0.51	0.00	<.0001	0.0410	Upregulated
hsa-miR-632	-0.51	0.13	0.0003	0.0820	Upregulated
***RUNX3*** (rs6672420)	AA (N=296)	AT/TT (N=770)			
hsa-miR-4261	1.38	0.51	<.0001	0.0820	Downregulated

Four SNPs, *BMPR1B* rs12508087, *BMPR1B* rs2120834, *BMPR2* rs2228545, and e*IF4EBP3* rs250425 were associated with differences in differential miRNA expression for colon tissue (Table 5). The majority of miRNAs associated with *BMPR1B* rs12508087 and rs2120834, and for *BMPR2* rs2228545 showed upregulated differential expression. Supplemental Table 4 shows all miRNAs whose differential expression was associated with TGFBeta signaling pathway SNPs in colon tissue for the variant allele of e*IF4EBP3* rs250425 were downregulated.

**Table 5:** Associations between SNPs in TGFB-signaling pathway and differential expression between colon carcinoma and normal mucosa

miRNA	Mean	Mean	P-values	FDR adjusted P	Direction
***BMPR1B*** (rs12508087)^1^	TT (N=378)	TA/AA (N=252)			
hsa-miR-1182	0.29	3.37	0.0008	0.0165	Upregulated
hsa-miR-1224-5p	-127.48	-16.55	0.0008	0.0165	Upregulated
hsa-miR-1225-5p	-422.11	83.75	0.0009	0.0165	Upregulated
hsa-miR-1228-3p	-2.66	2.94	0.0006	0.0165	Upregulated
hsa-miR-1233-1-5p	-26.41	-7.97	0.0012	0.0165	Upregulated
hsa-miR-1234-5p	-828.58	-28.93	0.0012	0.0165	Upregulated
hsa-miR-1268b	-24.76	179.09	0.0003	0.0165	Upregulated
hsa-miR-138-2-3p	-0.75	-1.86	0.0002	0.0165	Downregulated
hsa-miR-140-3p	-1.45	-2.78	<.0001	0.0165	Downregulated
hsa-miR-146b-5p	1.07	0.36	0.0004	0.0165	Downregulated
hsa-miR-150-3p	-31.45	11.22	0.0007	0.0165	Upregulated
hsa-miR-188-5p	-76.36	2.88	0.0010	0.0165	Upregulated
hsa-miR-1915-3p	-668.47	-162.98	0.0003	0.0165	Upregulated
hsa-miR-194-3p	-1.66	-0.88	0.0006	0.0165	Upregulated
hsa-miR-2861	-1203.17	-328.03	0.0002	0.0165	Upregulated
hsa-miR-3141	5.19	45.05	0.0011	0.0165	Upregulated
hsa-miR-345-3p	-1.80	7.61	0.0008	0.0165	Upregulated
hsa-miR-3621	-3.53	0.83	0.0011	0.0165	Upregulated
hsa-miR-3648	38.94	92.29	<.0001	0.0165	Upregulated
hsa-miR-3656	-298.14	144.78	0.0004	0.0165	Upregulated
hsa-miR-3665	-590.36	-110.88	0.0004	0.0165	Upregulated
hsa-miR-3940-5p	-127.51	-9.57	0.0008	0.0165	Upregulated
hsa-miR-4298	12.36	42.06	0.0009	0.0165	Upregulated
hsa-miR-4322	3.79	16.68	0.0003	0.0165	Upregulated
hsa-miR-4433-3p	-32.27	34.14	0.0008	0.0165	Upregulated
hsa-miR-4436a	-0.07	-0.82	0.0010	0.0165	Downregulated
hsa-miR-4463	-20.48	28.51	0.0005	0.0165	Upregulated
hsa-miR-4466	-264.72	32.20	0.0012	0.0165	Upregulated
hsa-miR-4507	-330.49	-70.42	0.0006	0.0165	Upregulated
hsa-miR-4508	-10.99	-5.08	0.0011	0.0165	Upregulated
hsa-miR-4640-5p	2.32	5.29	0.0005	0.0165	Upregulated
hsa-miR-4687-3p	-435.67	-17.28	0.0006	0.0165	Upregulated
hsa-miR-4688	1.16	5.27	0.0004	0.0165	Upregulated
hsa-miR-4690-5p	-54.41	-29.56	0.0010	0.0165	Upregulated
hsa-miR-4701-3p	-0.09	7.90	0.0011	0.0165	Upregulated
hsa-miR-4707-5p	-7.99	7.31	0.0006	0.0165	Upregulated
hsa-miR-4710	0.85	5.10	0.0011	0.0165	Upregulated
hsa-miR-4741	-91.90	96.96	0.0009	0.0165	Upregulated
hsa-miR-483-5p	3.36	25.50	0.0010	0.0165	Upregulated
hsa-miR-487b	0.04	-0.44	0.0011	0.0165	Downregulated
hsa-miR-498	-3.77	0.20	0.0008	0.0165	Upregulated
hsa-miR-5196-5p	6.80	16.80	0.0006	0.0165	Upregulated
hsa-miR-5585-3p	-7.93	25.37	0.0011	0.0165	Upregulated
hsa-miR-572	-95.60	-27.10	0.0007	0.0165	Upregulated
hsa-miR-6068	-507.73	-158.29	0.0005	0.0165	Upregulated
hsa-miR-6069	-1.29	2.00	0.0003	0.0165	Upregulated
hsa-miR-6076	-32.27	-1.35	0.0006	0.0165	Upregulated
hsa-miR-6083	-8.29	-4.69	0.0006	0.0165	Upregulated
hsa-miR-6088	-412.01	127.38	0.0012	0.0165	Upregulated
hsa-miR-6089	-5587.19	-1383.72	0.0010	0.0165	Upregulated
hsa-miR-6165	17.88	87.26	0.0008	0.0165	Upregulated
hsa-miR-623	-5.24	0.67	0.0010	0.0165	Upregulated
hsa-miR-638	-690.46	-126.32	0.0008	0.0165	Upregulated
hsa-miR-6511a-5p	1.31	6.67	0.0003	0.0165	Upregulated
hsa-miR-671-5p	-49.00	2.17	0.0003	0.0165	Upregulated
hsa-miR-6722-3p	-11.44	7.03	<.0001	0.0165	Upregulated
hsa-miR-6724-5p	-90.62	32.59	0.0012	0.0165	Upregulated
hsa-miR-718	-17.75	1.48	0.0004	0.0165	Upregulated
hsa-miR-937-5p	-43.41	42.52	0.0009	0.0165	Upregulated
hsa-miR-3937	-6.32	3.66	0.0013	0.0170	Upregulated
hsa-miR-4734	-24.78	-2.09	0.0013	0.0170	Upregulated
hsa-miR-6073	-1.63	-2.34	0.0013	0.0170	Downregulated
***BMPR1B*** (rs2120834)	GG/GC (N=545)	CC (N=85)			
hsa-miR-4638-3p	-0.02	0.66	<.0001	0.0813	Upregulated
***BMPR2*** (rs2228545)^1^	GG (N=598)	GA/AA (N=32)			
hsa-miR-3676-3p	0.06	3.22	<.0001	0.0406	Upregulated
hsa-miR-550a-5p	-0.86	1.65	<.0001	0.0406	Upregulated
***EIF4EBP3*** (rs250425)^1^	CC (N=398)	CT/TT (N=235)			
hsa-let-7g-5p	9.14	-2.75	0.0003	0.0406	Downregulated
hsa-miR-103a-3p	21.18	9.39	0.0005	0.0406	Downregulated
hsa-miR-141-3p	13.30	3.76	0.0004	0.0406	Downregulated
hsa-miR-15b-5p	9.35	1.64	<.0001	0.0406	Downregulated
hsa-miR-16-5p	16.07	-0.96	0.0004	0.0406	Downregulated
hsa-miR-194-5p	-20.67	-42.84	0.0004	0.0406	Downregulated
hsa-miR-200c-3p	36.61	6.04	0.0003	0.0406	Downregulated
hsa-miR-215	-20.96	-33.34	0.0004	0.0406	Downregulated
hsa-miR-30b-5p	2.26	-2.26	0.0004	0.0406	Downregulated
hsa-miR-92a-3p	62.24	45.02	0.0005	0.0406	Downregulated
hsa-miR-192-5p	-32.40	-59.23	0.0007	0.0474	Downregulated
hsa-miR-200a-3p	5.36	2.30	0.0007	0.0474	Downregulated
hsa-miR-107	13.80	6.52	0.0009	0.0488	Downregulated
hsa-miR-20a-5p	43.71	33.81	0.0009	0.0488	Downregulated
hsa-miR-29a-3p	58.54	37.06	0.0008	0.0488	Downregulated
hsa-miR-200b-3p	33.13	3.61	0.0011	0.0497	Downregulated
hsa-miR-27a-3p	31.16	23.35	0.0010	0.0497	Downregulated
hsa-miR-27b-3p	8.79	3.34	0.0011	0.0497	Downregulated

^1^ Data presented limited to the most significant adjusted p values; Supplementary Table 4 contains information for all associations with FDR <0.09.

*SMAD3* rs12901071, rs1498506, and rs2414937 were associated with differential miRNA expression in rectal tissue; however, only one miRNA was dysregulated for both rs12901071 and rs2414937. MiR-17-5p was upregulated by *SMAD3* rs12901071 and downregulated by *SMAD3* rs2414937 (Table 6). Additionally, *BMPR2* rs2228545 and *RUNX2* rs2819854 genotypes were associated with differential miRNA expression between rectal carcinoma and normal rectal mucosa. Both miR-324-5p and miR-484 were upregulated for the variant allele of *BMPR2* rs2228545 while all but two miRNAs were (Supplemental Table 5 shows associations with TGFBeta signaling pathway SNPs for all differentially expressed miRNAs in rectal tissue) downregulated in the variant allele of *RUNX2* rs2819854.

**Table 6:** Associations between SNPs in TGFB-signaling pathway and differential expression between paired rectal carcinoma and normal mucosa

miRNA	Mean	Mean	P-values	FDR adjusted P	Direction
***SMAD3*** (rs12901071)^1^	AA/AG (N=401)	GG (N=35)			
hsa-miR-106b-5p	8.99	10.77	0.0062	0.0633	Downregulated
hsa-miR-1202	-87.93	-307.26	0.0050	0.0633	Downregulated
hsa-miR-1207-5p	-271.79	-642.49	0.0037	0.0633	Downregulated
hsa-miR-1224-5p	-108.00	-243.68	0.0038	0.0633	Downregulated
hsa-miR-1226-5p	-3.96	-12.74	0.0049	0.0633	Downregulated
hsa-miR-1229-3p	-7.12	-11.15	0.0021	0.0633	Downregulated
hsa-miR-1233-1-5p	-26.35	-52.61	0.0061	0.0633	Downregulated
hsa-miR-1249	-1.87	-8.53	0.0023	0.0633	Downregulated
hsa-miR-1273c	-0.78	-5.34	0.0020	0.0633	Downregulated
hsa-miR-134	-35.39	-87.31	0.0011	0.0633	Downregulated
hsa-miR-149-3p	-2.88	-7.98	0.0046	0.0633	Downregulated
hsa-miR-17-5p	40.01	46.53	0.0030	0.0633	Upregulated
hsa-miR-188-5p	-52.14	-138.57	0.0026	0.0633	Downregulated
hsa-miR-197-5p	-238.29	-716.04	0.0036	0.0633	Downregulated
hsa-miR-19b-3p	17.46	20.65	0.0024	0.0633	Downregulated
hsa-miR-20b-5p	12.23	14.48	0.0018	0.0633	Upregulated
hsa-miR-26b-5p	0.16	2.92	0.0037	0.0633	Downregulated
hsa-miR-2861	-1175.18	-2275.40	0.0065	0.0633	Downregulated
hsa-miR-29b-3p	11.43	16.18	0.0002	0.0633	Upregulated
hsa-miR-29c-3p	1.32	5.20	0.0017	0.0633	Downregulated
hsa-miR-3132	-0.42	-4.66	0.0022	0.0633	Downregulated
hsa-miR-3188	-35.57	-68.70	0.0059	0.0633	Downregulated
hsa-miR-3194-5p	-15.89	-43.87	0.0063	0.0633	Downregulated
hsa-miR-3196	-223.46	-396.40	0.0047	0.0633	Downregulated
hsa-miR-3197	-3.13	-7.12	0.0055	0.0633	Downregulated
hsa-miR-34a-5p	8.57	11.04	0.0012	0.0633	Upregulated
hsa-miR-3610	-20.83	-45.81	0.0049	0.0633	Downregulated
hsa-miR-3621	-3.32	-8.78	0.0038	0.0633	Downregulated
hsa-miR-3665	-533.04	-1101.36	0.0066	0.0633	Downregulated
hsa-miR-3682-3p	-1.62	-12.28	0.0063	0.0633	Downregulated
hsa-miR-370	2.70	-4.18	0.0021	0.0633	Downregulated
hsa-miR-371a-5p	-7.65	-39.77	0.0021	0.0633	Downregulated
hsa-miR-3917	-3.25	-18.21	0.0045	0.0633	Downregulated
hsa-miR-3925-5p	-4.64	-10.52	0.0064	0.0633	Downregulated
hsa-miR-3934-3p	-2.65	-6.87	0.0007	0.0633	Downregulated
hsa-miR-3945	-7.76	-14.21	0.0056	0.0633	Downregulated
hsa-miR-4253	-2.64	-9.12	0.0009	0.0633	Downregulated
hsa-miR-4270	-20.58	-94.56	0.0042	0.0633	Downregulated
hsa-miR-4314	-3.87	-10.44	0.0057	0.0633	Downregulated
hsa-miR-4419b	-1.11	-4.38	0.0041	0.0633	Downregulated
hsa-miR-4430	-168.61	-399.75	0.0021	0.0633	Downregulated
hsa-miR-4476	-6.62	-16.39	0.0066	0.0633	Downregulated
hsa-miR-4481	-4.73	-16.10	0.0051	0.0633	Downregulated
hsa-miR-4486	-14.98	-50.49	0.0017	0.0633	Downregulated
hsa-miR-4487	-9.85	-25.84	0.0030	0.0633	Downregulated
hsa-miR-4497	-404.65	-1071.14	0.0006	0.0633	Downregulated
hsa-miR-4508	-11.27	-19.57	0.0058	0.0633	Downregulated
hsa-miR-4513	0.07	-3.34	0.0057	0.0633	Downregulated
hsa-miR-4516	-3221.62	-6327.76	0.0031	0.0633	Downregulated
hsa-miR-4530	-582.44	-2083.04	0.0007	0.0633	Downregulated
hsa-miR-4535	-5.24	-13.70	0.0043	0.0633	Downregulated
hsa-miR-4632-5p	-18.74	-47.70	0.0037	0.0633	Downregulated
hsa-miR-4646-5p	-2.94	-19.35	0.0042	0.0633	Downregulated
hsa-miR-4655-5p	-0.88	-6.36	0.0013	0.0633	Downregulated
hsa-miR-4656	-2.80	-21.20	0.0044	0.0633	Downregulated
hsa-miR-4665-5p	-3.35	-10.95	0.0030	0.0633	Downregulated
hsa-miR-4695-5p	-40.01	-97.81	0.0015	0.0633	Downregulated
hsa-miR-4721	-158.17	-394.68	0.0031	0.0633	Downregulated
hsa-miR-4734	-26.33	-64.44	0.0019	0.0633	Downregulated
hsa-miR-4739	-150.29	-326.46	0.0008	0.0633	Downregulated
hsa-miR-4740-5p	-1.84	-7.04	0.0022	0.0633	Downregulated
hsa-miR-4741	-61.75	-305.13	0.0028	0.0633	Downregulated
hsa-miR-4745-5p	-36.68	-76.35	0.0024	0.0633	Downregulated
hsa-miR-4758-5p	2.72	-16.93	0.0061	0.0633	Downregulated
hsa-miR-4763-3p	-115.00	-347.06	0.0055	0.0633	Downregulated
hsa-miR-4783-3p	-2.21	-5.03	0.0023	0.0633	Downregulated
hsa-miR-4788	-15.89	-64.61	0.0036	0.0633	Downregulated
hsa-miR-5001-5p	-220.39	-447.83	0.0063	0.0633	Downregulated
hsa-miR-5195-3p	-13.20	-40.01	0.0049	0.0633	Downregulated
***SMAD3*** (rs1498506)	AA (N=120)	AC/CC (N=316)			
hsa-miR-652-3p	0.05	0.63	<.0001	0.0825	Upregulated
***BMPR2*** (rs2228545)	GG (N=403)	GA/AA (N=33)			
hsa-miR-324-5p	1.42	3.90	0.0002	0.0825	Upregulated
hsa-miR-484	0.06	1.57	<.0001	0.0825	Upregulated
***SMAD3*** (rs2414937)^1^	GG/GC (N=418)	CC (N=18)			
hsa-miR-1273g-3p	-791.41	-65.42	0.0006	0.0550	Upregulated
hsa-miR-17-5p	41.32	22.24	0.0003	0.0550	Downregulated
hsa-miR-196b-5p	11.40	2.67	0.0003	0.0550	Downregulated
hsa-miR-20a-5p	48.80	28.38	0.0005	0.0550	Downregulated
hsa-miR-3651	34.71	18.55	0.0006	0.0550	Downregulated
hsa-miR-375	-46.53	-24.12	0.0005	0.0550	Upregulated
hsa-miR-4538	-42.83	-10.65	0.0004	0.0550	Upregulated
hsa-miR-4539	-23.96	-5.29	<.0001	0.0550	Upregulated
hsa-miR-4697-5p	-19.48	13.48	0.0003	0.0550	Upregulated
***RUNX2*** (rs2819854)^1^	CC/CT (N=341)	TT (N=95)			
hsa-miR-10b-5p	-2.34	1.92	0.0012	0.0715	Upregulated
hsa-miR-1182	1.20	-1.13	0.0008	0.0715	Downregulated
hsa-miR-1281	-9.98	-17.90	0.0013	0.0715	Downregulated
hsa-miR-129-5p	-1.16	-2.30	0.0008	0.0715	Downregulated
hsa-miR-3150b-5p	-3.63	-6.53	0.0013	0.0715	Upregulated
hsa-miR-3648	45.76	9.95	0.0009	0.0715	Downregulated
hsa-miR-365a-5p	-0.28	-2.66	0.0006	0.0715	Downregulated
hsa-miR-3911	10.28	1.46	0.0008	0.0715	Downregulated
hsa-miR-4665-3p	-20.84	-40.12	0.0012	0.0715	Downregulated
hsa-miR-4767	-2.92	-7.01	0.0009	0.0715	Downregulated
hsa-miR-4769-3p	-2.33	-4.60	0.0009	0.0715	Downregulated
hsa-miR-514b-5p	2.08	-1.03	0.0013	0.0715	Downregulated
hsa-miR-5585-3p	5.26	-36.48	<.0001	0.0715	Downregulated

^1^Data presented for most significant p values; Supplementary Table 5 contains information for all associations with FDR <0.09.

## DISCUSSION

The TGF-β-signaling pathway is one of the most important pathways involved in colorectal cancer development [32]. The TGF-β signaling pathway is involved in maintaining normal cell growth in a non-tumor cellular environment; alternatively, it can promote invasion and metastasis in neoplastic cells [27]. It has been proposed that miRNAs can act as intermediaries in a TGFB-miRNA pathway that both mediates cell growth and promotes invasive behavior [28]. Our data lend support to this dual role of the TGF-β signaling pathway. Distinct SNPs and associated miRNA expression were observed for normal colorectal mucosa and for differential expression of miRNAs between carcinoma and normal mucosa. MiRNA expression in normal mucosa reflects a non-tumor environment and the role of the TGF-β signaling pathway in maintaining normal cell growth. However, when examining differential expression between carcinoma and normal mucosa, associations suggest that the miRNA expression is more related to tumor promotion and possible metastatic potential.

One set of SNPs and genes associated with expression of miRNAs in normal mucosa consist of *eIF4E* and *TGFB1* and their receptors. These SNPs were previously shown to be associated with colon and rectal cancer risk [4, 6, 7]. eIF4E is essential for ribosomal recruitment and the initiation of translation [33]; eIF4E binds eIF4A and eIF4G to form the eIF4F complex that binds target mRNAs. In this system, eIF4E appears to be key in the regulation of translation initiation, as it physically binds the mRNA and is the least abundant initiation factor [34]; the interface of these genes with miRNAs is supported given the role of miRNAs is post-transcriptional. It has a sole phosphorylation site that interacts with either eIF4G or non-phosphorylated 4E-BP1; the PI3-Akt-mTOR pathway phosphorylates 4E-BP1 so that it releases eIF4E, allowing binding to eIF4G [34]. Thus eIF4E is important in the convergence of the TGF-β and Akt pathways, and its dysregulation is believed to be an important downstream regulator of Akt's action in tumorigenesis [6]. In the canonical TGF-β pathway, activated TGF-β receptors phosphorylate the c-terminal serine residues of the transcription factors in two different branches of the pathway [35]. In one branch, BMPs, a pleiotropic group of growth factors, are activated and signal through Smads 1/5/8 which act as transcription factors [36]. In the other branch, Smad2 and Smad3 (p-Smad 2/3) form heterotrimeric complexes with Smad 4, which translocate into the nucleus and regulate target gene expression [37]. *PTEN* rs532678 was associated with miRNA regulation in normal rectal mucosa. *PTEN* has been shown previously to influence the TGFβ pathway through downregulation of miRNAs [30].

The second role of the TGF-β signaling pathway, which is associated with greater metastatic potential, includes SNPs in *BMPs*, *RUNX*, *SMAD3*, and *NFκB1*. In this current study, we show that some of our previously observed SNPs associated with survival, play a role in differential miRNA expression between normal mucosa, colon, and rectal carcinoma tissues. This suggests that the second role of the TGF-β signaling pathway, i.e. greater metastatic potential, may be at play. The miRNAs associated with SNPs in *SMAD3* and *RUNX* for differential expression were different from those associated with these SNPs in normal mucosa, further suggesting different mechanisms by which genes and corresponding pathways are being regulated. These genes have previously been shown to play various roles in phenotype aggression, angiogenesis, and metastasis. BMPs are pleiotropic growth factors, whose dysregulation has been shown to deregulate colonic stem cell renewal in mouse models, allowing for de novo crypt formation [36]. In addition to their contribution to tumor initiation, BMP dysregulation has also been shown to play a role in angiogenesis [38, 39]. *RUNX2*, has been associated with survival and aggressive phenotype in sarcoma [40]. Likewise, in colon cancer, *RUNX2* has been significantly associated with Dukes staging, liver metastasis, and ERβ status [41]. In mice models, *SMAD3* loss has been associated with mRNA expression of VEGF, MCP-1, and IL-6 in the choroid [42]. Similarly, in colon cancer these cytokines have been associated with the tumor microenvironment, including angiogenesis. Moreover, in drug resistant colorectal cancer cells 5-flourouracil (5-FU) can stimulate Smad3, which is believed to contribute to drug resistance [43]. We have previously shown that interferons, possibly through NF-kB signaling, are associated with colon and rectal cancer risk and survival [44]. It is possible that the diversity of mechanisms associated with these genes and SNPs is through their regulation of miRNAs, which in turn influence expression of hundreds of other genes and pathways.

We have previously reported that *SMAD3* was associated with survival in rectal cancer [3]. Of the *SMAD3* SNPs associated with differential miRNA expression by genotype, *SMAD3* rs12708492 and rs2414937 were previously shown to their influence on survival in rectal and colon tumors cancers [6]. Here we find that rs2414037 is associated with differential miRNA expression between normal mucosa, colon, and rectal carcinoma tissues overall as well as for rectal tumors specifically; suggesting that miRNA dysregulation may be responsible for some of our previously documented observations with regards to the TGF-β pathway and survival. *RUNX2* also has been found to be associated with colon and rectal cancer. We previously found *RUNX2*, particularly interactions between these SNPs in *RUNX2* and *SMAD3*, showed strong interaction to increase risk for rectal cancer, as well as prognostic implications [6].

As we have previously shown [4] several SNPs associated with genes in this pathway interact with each other and with lifestyle factors to alter risk of colorectal cancer. Thus, this is a complex pathway. In this study we have only examined the main effects of the SNPs on levels of miRNA expression, although it should be acknowledged that pathways are more complicated given the extensive interaction between SNPs and other genetic and lifestyle factors. Interpretation of results is also complicated by the difficulty in assessing functionality of specific miRNAs. Given that each miRNA can regulated 100s or even 1000s of genes, there is a general lack of specificity in determining functionality in any specific setting. It is hoped that over time, as more information on miRNAs become available, determining specific functionality will become more straightforward. Despite these limitations, we believe that the data presented is making strides in understanding how miRNAs work as well as how specific genes in the TGF-β-signaling pathway operate. The study represents a large collection of colon and rectal data with paired normal and carcinoma tissue. We used the Agilent platform that allowed us to examine almost 1000 miRNAs commonly expressed in colorectal tissue. The data obtained from this platform has been shown to have both high repeatability and validity in terms of comparisons with qPCR expression data. Because studies such as the one presented here are relatively few, we encourage others to conduct similar studies to build on and confirm our results.

In summary we have shown that genetic variation in the TGF-β-signaling pathway influences expression of miRNAs in colorectal tissue. While some genes and their related SNPs influenced miRNA expression in normal tissue, another set influences differential expression between carcinoma and normal mucosa. These results provide support for the functionality of these SNPs that previously have been associated with either colorectal cancer risk or survival, but also provide insight into how expression levels of miRNAs can be altered. The influence on miRNA expression by SNPs in the TGF-β-signaling pathway and the influence of miRNAs on 100s of targeted genes embody the diversity and importance of the TGF-β-signaling pathway in the carcinogenic process.

## MATERIALS AND METHODS

### Study participants

Study participants were recruited as part of two population-based case-control studies that included all incident colon and rectal cancer between 30 to 79 years of age who resided in Utah or were members of the Kaiser Permanente Medical Care Program (KPMCP) in Northern California. Participants were white, Hispanic, or black for the colon cancer study and also included participants of Asian race for the rectal portion of the study [45, 46]. Case diagnosis was verified by tumor registry data as a first primary adenocarcinoma of the colon and were diagnosed between October 1991 and September 1994 and for rectal were diagnosed between May 1997 and May 2001. Detailed study methods have been described [47]. The Institutional Review Boards of the University of Utah and at KPMCP approved the study. In this study we included 1188 participants for whom we have genotype and miRNA data.

### miRNA processing

Formalin-fixed paraffin embedded tissue from the initial biopsy or surgery was used to extract RNA. Carcinoma tissue and adjacent normal mucosa were used to make RNA. Cells were dissected from 1-4 sequential sections on aniline blue stained slides using an H&E slide for reference. Total RNA was extracted, isolated, and purified using the RecoverAll Total Nucleic Acid isolation kit (Ambion); RNA yields were determined using a NanoDrop spectrophotometer.

### miRNA

The Agilent Human miRNA Microarray V19.0 was used due to the number of miRNAs, its high level of reliability (repeatability coefficient was 0.98 in our data), and the amount of RNA needed to run the platform. The microarray contains probes for 2006 unique human miRNAs as described previously. Data were required to pass stringent QC parameters established by Agilent that included tests for excessive background fluorescence, excessive variation among probe sequence replicates on the array, and measures of the total gene signal on the array to assess low signal. If samples failed to meet quality standards for any of these parameters, the sample was re-labeled, hybridized to arrays, and scanned again. If a sample failed QC assessment a second time, the sample was deemed to be of poor quality and the sample was excluded from analysis. Our previous analysis has shown that the repeatability associated with this microarray was extremely high (r=0.98) [47], and that comparison of miRNA expression levels obtained from the Agilent microarray to those obtained from qPCR had an agreement of 100% in terms of directionality of findings and that the fold change calculated for the miRNA expression difference between carcinoma and normal colonic mucosa was almost identical [48]. Of the 2006 unique human miRNAs assessed on the Agilent microarray, 1394 were expressed in colorectal tissue, 1226 in colorectal carcinoma tissue, and 1179 in normal colorectal mucosa. We further restricted the analysis to those miRNAs for which at least 20% of the population showed expression, leaving 820 miRNAs for colorectal cancer, 813 miRNAs for colon cancer, and 825 miRNAs for rectal cancer for analysis.

To normalize differences in miRNA expression that could be attributed to the array, amount of RNA, location on array, or factors that could erroneously influence miRNA expression levels, total gene signal was normalized by multiplying each sample by a scaling factor [49], which was the median of the 75^th^ percentiles of all the samples divided by the individual 75^th^ percentile of each sample.

### TagSNP selection and genotyping

A customized GoldenGate chemistry (Illumina, San Diego, California) platform was assembled based on genes associated with the CHIEF (Convergence of Hormones, Inflammation, and Energy-related Factors) pathway [7, 26, 32, 50]. TagSNPs for genes in this pathway were selected using the following parameters: an r^2^=0.8 defined linkage disequilibrium (LD) blocks using a Caucasian LD map, minor allele frequency or minor allele frequency (MAF) >0.1, range= -1500 bps from the initiation codon to +1500 bps from the termination codon, and 1 SNP/LD bin. A genotyping call rate of 99.85% was attained. Blinded internal replicates represented 4.4% of the sample set. The duplicate concordance rate was 100.00%. In this analysis we focused on 21 genes, *BMP1*, *BMP2*, *BMP4*, *BMPR1A*, *BMPR1B*, *BMPR2*, e*IF4E*, *eIF4EBP3*, *GDF10*, *MAPK1*, *MTOR*, *NFκB1*, *PTEN*, *RUNX1*, *RUNX2*, *RUNX3*, *SMAD2*, *SMAD3*, *SMAD4*, *SMAD7*, *TGFB1*, and *TGFBR1*.

### Statistical methods

We assessed 80 SNPs in 21 genes that we previously have shown to be associated with colon or rectal cancer risk [3, 6, 7, 26, 32, 51] in 1188 individuals who also have miRNA expression in normal mucosa and 1141 who have both carcinoma and normal mucosa miRNA expression. Online Supplemental Table 6 show all SNPs assessed. Analyses were run separately for colon and rectal cancer as well as for colorectal cancer combined. Log base 2 transformed miRNA expression level data were used to determine if miRNA expression levels were significantly different by genotype for each SNP. For the SNPs assessed, we used either a dominant or recessive model of inheritance based on previous reports (See Supplementary Table 7). To determine statistical significance, we fit a least-squares linear regression model to the miRNA expression levels and SNPs, adjusting for age at diagnosis, sex, and race/ethnicity. P-values were generated using the bootstrap method by creating a distribution of 10,000 F statistics derived by resampling the residuals from the null hypothesis of no association between the SNPs and the miRNA (Davison and Hinkley reference), using the ‘boot’ package in R. Associations were considered important if the false discovery rate (FDR) was less than 0.09 as described by Benjamini and Hochberg [52]; these associations had q-values less than 0.05 [53]. We present those most significant in the text and attach online supplements for those with a FDR of less than 0.09 (Supplementary Tables 1, 2, 3, 4, 5). Analyses were run for colorectal normal mucosa (Table 2) as well as for unique association in normal mucosa by sub-site of the tissue, i.e. colon or rectal (Table 3). Similar analysis were run for differential expression (carcinoma – normal) for colorectal cancer overall (Table 4) and for colorectal sub-site (Table 5 for colon and Table 6 for rectal).

## SUPPLEMENTARY TABLES




