# Supplementary material for: Genetic variants in the TGFβ-signaling pathway influence expression of miRNAs in colon and rectal normal mucosa and tumor tissue
Source: Oncotarget. 2017 Jan 5;8(10):16765–83. doi: 10.18632/oncotarget.14508 (PMC5370000; doi:10.18632/oncotarget.14508)
Supplement: Supplementary file 1 [file oncotarget-08-16765-s001.pdf]

Genetic variants in the TGFβ-signaling pathway influence expression of miRNAs in colon and rectal normal mucosa and tumor tissue

SUPPLEMENTARY TABLES

Supplementary Table 1: All miRNAs associated with TGFBeta signaling pathway SNPs in overall colorectal normal mucosa; FDR <0.09

See Supplementary File 1

Supplementary Table 2: SNPs in TGFBeta signaling pathway associated with miRNAs in normal colonic mucosa; FDR<0.09

See Supplementary File 1

Supplementary Table 3: SNPs in TGFBeta-Signaling Pathway Associated with miRNA in normal rectal mucosa; FDR<0.09

See Supplementary File 1

Supplementary Table 4: SNPs in TGFBeta-signaling pathway associated with miRNAs in differential colon tissue; FDR<0.09

See Supplementary File 1

Supplementary Table 5: TGFBeta Pathway SNPs associated with miRNAs in differential rectal tissue; FDR<0.09

See Supplementary File 1

Supplementary Table 6: Summary of genes and SNPs assessed

Gene	SNP ID
BMP1	rs12114940, rs3857979, rs4076873
BMP2	rs3178250
BMP4	rs17563
BMPR1A	rs12765929, rs2168730, rs2883420, rs7088641, rs7895217
BMPR1B	rs12508087, rs13134042, rs17616243, rs1863652, rs2120834, rs2214395, rs6849425, rs7694043, rs7698964
BMPR2	rs12477602, rs2228545, rs6751210
eIF4E	rs11727086, rs12498533
eIF4EBP3	rs250425
GDF10	rs12769499, rs2853838, rs762454
MAPK1	rs11913721
MTOR	rs1057079
NFκB1	rs11722146, rs230510, rs3821958, rs4648090
PTEN	rs532678
RUNX1	rs11701453, rs11702779, rs1475840, rs1883066, rs2242878, rs2253319, rs2300395, rs2834645, rs2834670, rs7279123, rs7280028, rs8134179
RUNX2	rs10948238, rs12208240, rs12333172, rs2819854
RUNX3	rs2135756, rs2236850, rs6672420, rs7517302
SMAD2	rs1787199, rs1792689, rs4940086
SMAD3	rs11639295, rs12708492, rs12901071, rs12904944, rs1498506, rs16950687, rs17293443, rs2414937, rs3743343, rs3825977, rs7163381, rs7176870, rs7181556
SMAD7	rs12953717, rs3736242, rs4939827, rs4939832
TGFB1	rs1800469, rs4803455
TGFBR1	rs10733710, rs1571590, rs6478974

Supplementary Table 7: Summary of model used and previously identified associations for colorectal cancer risk and survival

See Supplementary File 1
