# Supplementary material for: Genetic variants in the TGFβ-signaling pathway influence expression of miRNAs in colon and rectal normal mucosa and tumor tissue
Source: Oncotarget. 2017 Jan 5;8(10):16765–83. doi: 10.18632/oncotarget.14508 (PMC5370000; doi:10.18632/oncotarget.14508)
Supplement: Supplementary file 2 [file oncotarget-08-16765-s002.docx]

| Supplemental Table 1. All miRNAs associated with TGFBeta signaling pathway SNPs in overall colorectal normal mucosa; FDR <0.09 | | | | | |
| --- | --- | --- | --- | --- | --- |
| miRNA | Mean | Mean | P-values | FDR adjusted P | Direction |
| ***EIF4E* (rs12498533)** | AA ( N=342 ) | AC/CC ( N=798 ) |  |  |  |
| hsa-miR-221-3p | 3.21 | 2.25 | <.0001 | 0.0820 | Downregulated |
| ***TGFBR1* (rs1571590)** | AA ( N=738 ) | AG/GG ( N=372 ) |  |  |  |
| hsa-miR-100-5p | 7.83 | 6.06 | <.0001 | 0.0373 | Downregulated |
| hsa-miR-130a-3p | 3.20 | 2.26 | 0.0004 | 0.0373 | Downregulated |
| hsa-miR-143-3p | 7.23 | 5.89 | 0.0004 | 0.0373 | Downregulated |
| hsa-miR-19b-3p | 8.53 | 6.67 | 0.0003 | 0.0373 | Downregulated |
| hsa-miR-29c-3p | 15.56 | 13.19 | 0.0005 | 0.0373 | Downregulated |
| hsa-miR-3666 | 19.31 | 20.74 | <.0001 | 0.0373 | Upregulated |
| hsa-miR-4324 | 1.30 | 0.98 | 0.0002 | 0.0373 | Downregulated |
| hsa-miR-4655-3p | 22.54 | 24.35 | 0.0005 | 0.0373 | Upregulated |
| hsa-miR-4659a-3p | 27.57 | 29.81 | 0.0003 | 0.0373 | Upregulated |
| hsa-miR-5003-3p | 18.59 | 20.12 | 0.0005 | 0.0373 | Upregulated |
| hsa-miR-6500-5p | 18.18 | 19.53 | 0.0005 | 0.0373 | Upregulated |
| hsa-miR-662 | 41.11 | 43.73 | 0.0006 | 0.0410 | Upregulated |
| hsa-miR-140-3p | 7.16 | 6.09 | 0.0008 | 0.0492 | Downregulated |
| hsa-miR-26a-5p | 86.12 | 75.56 | 0.0009 | 0.0492 | Downregulated |
| hsa-miR-4294 | 21.48 | 22.41 | 0.0009 | 0.0492 | Upregulated |
| hsa-miR-5088 | 53.35 | 57.08 | 0.0010 | 0.0512 | Upregulated |
| hsa-miR-222-3p | 9.02 | 8.24 | 0.0012 | 0.0518 | Downregulated |
| hsa-miR-3663-5p | 12.54 | 13.20 | 0.0012 | 0.0518 | Upregulated |
| hsa-miR-93-5p | 12.88 | 11.21 | 0.0012 | 0.0518 | Downregulated |
| hsa-miR-151b | 4.36 | 3.75 | 0.0015 | 0.0615 | Downregulated |
| hsa-miR-601 | 43.92 | 47.05 | 0.0016 | 0.0625 | Upregulated |
| hsa-miR-5001-5p | 1092.69 | 1182.58 | 0.0017 | 0.0634 | Upregulated |
| hsa-miR-532-3p | 0.67 | 0.41 | 0.0018 | 0.0642 | Downregulated |
| hsa-miR-195-5p | 9.68 | 7.98 | 0.0021 | 0.0668 | Downregulated |
| hsa-miR-26b-5p | 17.84 | 15.39 | 0.0022 | 0.0668 | Downregulated |
| hsa-miR-4253 | 43.85 | 46.38 | 0.0021 | 0.0668 | Upregulated |
| hsa-miR-572 | 474.34 | 510.41 | 0.0022 | 0.0668 | Upregulated |
| hsa-miR-361-5p | 5.18 | 4.31 | 0.0023 | 0.0674 | Downregulated |
| hsa-miR-1539 | 2.41 | 1.95 | 0.0025 | 0.0707 | Downregulated |
| hsa-miR-199a-5p | 7.39 | 6.15 | 0.0026 | 0.0711 | Downregulated |
| hsa-miR-5190 | 28.36 | 29.53 | 0.0029 | 0.0767 | Upregulated |
| hsa-miR-3185 | 16.79 | 17.90 | 0.0030 | 0.0769 | Upregulated |
| hsa-miR-3621 | 35.98 | 37.88 | 0.0031 | 0.0770 | Upregulated |
| hsa-miR-155-5p | 43.53 | 45.59 | 0.0033 | 0.0796 | Upregulated |
| hsa-miR-365a-3p | 2.81 | 2.00 | 0.0036 | 0.0843 | Downregulated |
| ***NFKB1* (rs230510)** | AA ( N=387 ) | AT/TT ( N=723 ) |  |  |  |
| hsa-miR-4446-3p | 16.16 | 13.90 | <.0001 | 0.0820 | Downregulated |
| ***SMAD3* (rs3743343)** | TT/TC ( N=1035 ) | CC ( N=75 ) |  |  |  |
| hsa-miR-1285-3p | 19.98 | 17.98 | <.0001 | 0.0410 | Downregulated |
| hsa-miR-3935 | 16.98 | 14.78 | <.0001 | 0.0410 | Downregulated |
| hsa-miR-758-5p | 31.75 | 28.75 | 0.0003 | 0.0820 | Downregulated |
| ***TGFB1* (rs4803455)** | CC ( N=275 ) | CA/AA ( N=881 ) |  |  |  |
| hsa-miR-146b-5p | 1.48 | 0.92 | 0.0002 | 0.0820 | Downregulated |
| hsa-miR-3609 | 1.68 | 1.20 | <.0001 | 0.0820 | Downregulated |
| ***TGFBR1* (rs6478974)** | TT ( N=359 ) | TA/AA ( N=750 ) |  |  |  |
| hsa-miR-1226-5p | 54.62 | 51.50 | 0.0011 | 0.0355 | Downregulated |
| hsa-miR-151a-5p | 7.57 | 9.03 | 0.0011 | 0.0355 | Upregulated |
| hsa-miR-1587 | 1405.23 | 1292.11 | 0.0013 | 0.0355 | Downregulated |
| hsa-miR-199a-3p | 16.51 | 19.29 | 0.0005 | 0.0355 | Upregulated |
| hsa-miR-200a-3p | 18.50 | 20.89 | 0.0006 | 0.0355 | Upregulated |
| hsa-miR-20a-5p | 13.21 | 15.67 | 0.0016 | 0.0355 | Upregulated |
| hsa-miR-214-3p | 4.76 | 5.65 | 0.0005 | 0.0355 | Upregulated |
| hsa-miR-222-3p | 8.28 | 8.99 | 0.0016 | 0.0355 | Upregulated |
| hsa-miR-22-3p | 12.78 | 14.57 | 0.0007 | 0.0355 | Upregulated |
| hsa-miR-25-3p | 8.70 | 10.46 | 0.0016 | 0.0355 | Upregulated |
| hsa-miR-26b-5p | 15.33 | 17.83 | 0.0004 | 0.0355 | Upregulated |
| hsa-miR-30c-5p | 7.73 | 9.17 | 0.0012 | 0.0355 | Upregulated |
| hsa-miR-3156-5p | 95.91 | 90.85 | 0.0009 | 0.0355 | Downregulated |
| hsa-miR-3185 | 17.99 | 16.77 | 0.0005 | 0.0355 | Downregulated |
| hsa-miR-33b-3p | 19.25 | 15.53 | 0.0006 | 0.0355 | Downregulated |
| hsa-miR-3620-5p | 95.85 | 89.60 | 0.0015 | 0.0355 | Downregulated |
| hsa-miR-3621 | 38.13 | 35.90 | 0.0015 | 0.0355 | Downregulated |
| hsa-miR-370 | 41.68 | 39.44 | 0.0002 | 0.0355 | Downregulated |
| hsa-miR-3917 | 99.12 | 93.52 | 0.0005 | 0.0355 | Downregulated |
| hsa-miR-3934-3p | 29.59 | 28.27 | 0.0014 | 0.0355 | Downregulated |
| hsa-miR-3937 | 70.06 | 65.67 | 0.0013 | 0.0355 | Downregulated |
| hsa-miR-4253 | 46.35 | 43.92 | 0.0012 | 0.0355 | Downregulated |
| hsa-miR-4294 | 22.41 | 21.50 | 0.0011 | 0.0355 | Downregulated |
| hsa-miR-4535 | 70.15 | 66.72 | 0.0007 | 0.0355 | Downregulated |
| hsa-miR-4655-5p | 34.25 | 32.23 | 0.0013 | 0.0355 | Downregulated |
| hsa-miR-4695-5p | 385.66 | 365.84 | 0.0009 | 0.0355 | Downregulated |
| hsa-miR-4726-5p | 22.13 | 21.13 | 0.0015 | 0.0355 | Downregulated |
| hsa-miR-4734 | 210.70 | 198.06 | 0.0013 | 0.0355 | Downregulated |
| hsa-miR-5196-5p | 63.33 | 58.28 | 0.0012 | 0.0355 | Downregulated |
| hsa-miR-550a-3-5p | 28.29 | 26.77 | 0.0009 | 0.0355 | Downregulated |
| hsa-miR-564 | 67.17 | 64.76 | 0.0014 | 0.0355 | Downregulated |
| hsa-miR-601 | 46.91 | 44.06 | 0.0010 | 0.0355 | Downregulated |
| hsa-miR-6084 | 18.25 | 16.81 | 0.0012 | 0.0355 | Downregulated |
| hsa-miR-610 | 25.93 | 24.45 | 0.0013 | 0.0355 | Downregulated |
| hsa-miR-6511b-5p | 65.88 | 60.79 | 0.0005 | 0.0355 | Downregulated |
| hsa-miR-760 | 34.80 | 32.70 | 0.0014 | 0.0355 | Downregulated |
| hsa-miR-93-5p | 10.92 | 12.98 | <.0001 | 0.0355 | Upregulated |
| hsa-miR-15b-5p | 20.02 | 23.05 | 0.0019 | 0.0359 | Upregulated |
| hsa-miR-3187-3p | 9.99 | 9.16 | 0.0021 | 0.0359 | Downregulated |
| hsa-miR-4646-5p | 112.80 | 106.87 | 0.0021 | 0.0359 | Downregulated |
| hsa-miR-4738-3p | 50.24 | 48.07 | 0.0020 | 0.0359 | Downregulated |
| hsa-miR-4740-5p | 33.39 | 31.96 | 0.0017 | 0.0359 | Downregulated |
| hsa-miR-5189 | 33.40 | 31.42 | 0.0021 | 0.0359 | Downregulated |
| hsa-miR-550b-2-5p | 38.12 | 35.90 | 0.0020 | 0.0359 | Downregulated |
| hsa-miR-6124 | 522.71 | 487.27 | 0.0018 | 0.0359 | Downregulated |
| hsa-miR-6165 | 323.94 | 306.32 | 0.0021 | 0.0359 | Downregulated |
| hsa-miR-6500-5p | 19.30 | 18.32 | 0.0019 | 0.0359 | Downregulated |
| hsa-miR-671-5p | 291.07 | 271.09 | 0.0018 | 0.0359 | Downregulated |
| hsa-miR-6722-3p | 103.52 | 97.67 | 0.0022 | 0.0368 | Downregulated |
| hsa-miR-196b-5p | 3.53 | 4.76 | 0.0023 | 0.0370 | Upregulated |
| hsa-miR-4481 | 84.65 | 78.48 | 0.0023 | 0.0370 | Downregulated |
| hsa-miR-4758-5p | 131.05 | 120.75 | 0.0024 | 0.0378 | Downregulated |
| hsa-miR-1247-3p | 26.28 | 25.11 | 0.0028 | 0.0396 | Downregulated |
| hsa-miR-199a-5p | 6.22 | 7.34 | 0.0027 | 0.0396 | Upregulated |
| hsa-miR-345-3p | 59.74 | 56.13 | 0.0026 | 0.0396 | Downregulated |
| hsa-miR-3622b-5p | 38.67 | 36.86 | 0.0028 | 0.0396 | Downregulated |
| hsa-miR-451a | 25.98 | 32.37 | 0.0029 | 0.0396 | Upregulated |
| hsa-miR-5001-5p | 1180.01 | 1096.07 | 0.0028 | 0.0396 | Downregulated |
| hsa-miR-6511a-5p | 43.38 | 40.66 | 0.0029 | 0.0396 | Downregulated |
| hsa-miR-6724-5p | 886.55 | 827.04 | 0.0027 | 0.0396 | Downregulated |
| hsa-miR-28-5p | 0.65 | 0.97 | 0.0030 | 0.0397 | Upregulated |
| hsa-miR-30b-5p | 17.20 | 19.78 | 0.0030 | 0.0397 | Upregulated |
| hsa-miR-1227-5p | 1055.30 | 983.03 | 0.0031 | 0.0398 | Downregulated |
| hsa-miR-3138 | 80.25 | 76.45 | 0.0033 | 0.0398 | Downregulated |
| hsa-miR-3666 | 20.58 | 19.41 | 0.0032 | 0.0398 | Downregulated |
| hsa-miR-4271 | 281.52 | 263.83 | 0.0034 | 0.0398 | Downregulated |
| hsa-miR-4322 | 100.06 | 93.99 | 0.0033 | 0.0398 | Downregulated |
| hsa-miR-4442 | 294.85 | 280.61 | 0.0033 | 0.0398 | Downregulated |
| hsa-miR-5195-3p | 180.15 | 169.16 | 0.0032 | 0.0398 | Downregulated |
| hsa-miR-877-5p | 38.97 | 37.47 | 0.0034 | 0.0398 | Downregulated |
| hsa-miR-1299 | 26.80 | 25.05 | 0.0035 | 0.0399 | Downregulated |
| hsa-miR-4507 | 2095.54 | 1947.22 | 0.0035 | 0.0399 | Downregulated |
| hsa-miR-26a-5p | 75.68 | 85.89 | 0.0037 | 0.0404 | Upregulated |
| hsa-miR-30e-5p | 2.36 | 3.06 | 0.0037 | 0.0404 | Upregulated |
| hsa-miR-3679-5p | 784.73 | 728.51 | 0.0036 | 0.0404 | Downregulated |
| hsa-miR-1914-3p | 132.86 | 126.50 | 0.0038 | 0.0410 | Downregulated |
| hsa-miR-195-3p | 0.52 | 0.68 | 0.0039 | 0.0410 | Upregulated |
| hsa-miR-361-5p | 4.26 | 5.19 | 0.0039 | 0.0410 | Upregulated |
| hsa-miR-100-5p | 6.37 | 7.65 | 0.0041 | 0.0415 | Upregulated |
| hsa-miR-143-3p | 5.98 | 7.17 | 0.0041 | 0.0415 | Upregulated |
| hsa-miR-19b-3p | 6.87 | 8.40 | 0.0041 | 0.0415 | Upregulated |
| hsa-miR-10a-5p | 19.59 | 22.76 | 0.0049 | 0.0418 | Upregulated |
| hsa-miR-126-3p | 12.03 | 14.21 | 0.0042 | 0.0418 | Upregulated |
| hsa-miR-155-5p | 45.50 | 43.62 | 0.0049 | 0.0418 | Downregulated |
| hsa-miR-16-5p | 53.14 | 59.45 | 0.0047 | 0.0418 | Upregulated |
| hsa-miR-195-5p | 8.02 | 9.63 | 0.0046 | 0.0418 | Upregulated |
| hsa-miR-2392 | 225.31 | 208.12 | 0.0046 | 0.0418 | Downregulated |
| hsa-miR-27b-3p | 20.99 | 23.95 | 0.0046 | 0.0418 | Upregulated |
| hsa-miR-4314 | 60.71 | 57.51 | 0.0045 | 0.0418 | Downregulated |
| hsa-miR-4429 | 25.75 | 24.14 | 0.0043 | 0.0418 | Downregulated |
| hsa-miR-4463 | 319.67 | 302.25 | 0.0043 | 0.0418 | Downregulated |
| hsa-miR-4470 | 41.11 | 39.38 | 0.0046 | 0.0418 | Downregulated |
| hsa-miR-4665-5p | 60.64 | 57.98 | 0.0047 | 0.0418 | Downregulated |
| hsa-miR-4685-5p | 23.46 | 22.34 | 0.0044 | 0.0418 | Downregulated |
| hsa-miR-4689 | 126.30 | 119.34 | 0.0048 | 0.0418 | Downregulated |
| hsa-miR-623 | 48.16 | 45.28 | 0.0050 | 0.0418 | Downregulated |
| hsa-miR-638 | 4139.82 | 3897.90 | 0.0050 | 0.0418 | Downregulated |
| hsa-miR-937-5p | 653.36 | 607.60 | 0.0049 | 0.0418 | Downregulated |
| hsa-miR-5003-3p | 19.87 | 18.74 | 0.0051 | 0.0422 | Downregulated |
| hsa-miR-1236-5p | 176.12 | 166.33 | 0.0054 | 0.0425 | Downregulated |
| hsa-miR-140-3p | 6.19 | 7.09 | 0.0054 | 0.0425 | Upregulated |
| hsa-miR-194-5p | 97.15 | 107.17 | 0.0053 | 0.0425 | Upregulated |
| hsa-miR-21-3p | 7.90 | 8.85 | 0.0053 | 0.0425 | Upregulated |
| hsa-miR-3154 | 40.05 | 38.83 | 0.0054 | 0.0425 | Downregulated |
| hsa-miR-4707-5p | 94.67 | 87.59 | 0.0055 | 0.0425 | Downregulated |
| hsa-miR-4788 | 336.45 | 319.61 | 0.0055 | 0.0425 | Downregulated |
| hsa-miR-1268a | 1009.79 | 945.37 | 0.0056 | 0.0429 | Downregulated |
| hsa-miR-557 | 75.03 | 70.42 | 0.0057 | 0.0433 | Downregulated |
| hsa-let-7d-5p | 27.91 | 31.33 | 0.0061 | 0.0440 | Upregulated |
| hsa-miR-125a-5p | 9.53 | 10.78 | 0.0065 | 0.0440 | Upregulated |
| hsa-miR-1471 | 247.69 | 230.28 | 0.0062 | 0.0440 | Downregulated |
| hsa-miR-17-5p | 12.69 | 14.46 | 0.0064 | 0.0440 | Upregulated |
| hsa-miR-3127-5p | 37.16 | 34.92 | 0.0062 | 0.0440 | Downregulated |
| hsa-miR-3654 | 32.69 | 31.63 | 0.0060 | 0.0440 | Downregulated |
| hsa-miR-4486 | 190.85 | 181.00 | 0.0066 | 0.0440 | Downregulated |
| hsa-miR-4539 | 80.55 | 76.60 | 0.0066 | 0.0440 | Downregulated |
| hsa-miR-4647 | 39.97 | 38.07 | 0.0065 | 0.0440 | Downregulated |
| hsa-miR-4710 | 40.20 | 38.34 | 0.0064 | 0.0440 | Downregulated |
| hsa-miR-4721 | 1737.92 | 1656.12 | 0.0064 | 0.0440 | Downregulated |
| hsa-miR-497-5p | 3.75 | 4.64 | 0.0065 | 0.0440 | Upregulated |
| hsa-miR-572 | 507.74 | 476.52 | 0.0066 | 0.0440 | Downregulated |
| hsa-miR-617 | 51.89 | 49.47 | 0.0062 | 0.0440 | Downregulated |
| hsa-miR-99b-3p | 59.49 | 57.08 | 0.0065 | 0.0440 | Downregulated |
| hsa-miR-125a-3p | 244.50 | 234.90 | 0.0068 | 0.0442 | Downregulated |
| hsa-miR-4466 | 1977.82 | 1878.30 | 0.0068 | 0.0442 | Downregulated |
| hsa-miR-4787-5p | 1427.02 | 1331.00 | 0.0067 | 0.0442 | Downregulated |
| hsa-miR-4476 | 74.89 | 71.48 | 0.0070 | 0.0448 | Downregulated |
| hsa-miR-874 | 225.27 | 215.75 | 0.0070 | 0.0448 | Downregulated |
| hsa-miR-4505 | 3398.85 | 3177.83 | 0.0071 | 0.0451 | Downregulated |
| hsa-miR-4632-5p | 218.81 | 208.75 | 0.0072 | 0.0454 | Downregulated |
| hsa-miR-134 | 285.25 | 273.10 | 0.0076 | 0.0462 | Downregulated |
| hsa-miR-141-3p | 24.67 | 27.58 | 0.0074 | 0.0462 | Upregulated |
| hsa-miR-652-5p | 83.09 | 78.70 | 0.0075 | 0.0462 | Downregulated |
| hsa-miR-887 | 61.24 | 58.80 | 0.0076 | 0.0462 | Downregulated |
| hsa-miR-99b-5p | 1.88 | 2.48 | 0.0075 | 0.0462 | Upregulated |
| hsa-miR-1229-5p | 694.56 | 651.97 | 0.0078 | 0.0463 | Downregulated |
| hsa-miR-2276 | 96.38 | 91.97 | 0.0083 | 0.0463 | Downregulated |
| hsa-miR-3132 | 39.50 | 38.10 | 0.0078 | 0.0463 | Downregulated |
| hsa-miR-3147 | 34.21 | 32.49 | 0.0078 | 0.0463 | Downregulated |
| hsa-miR-3188 | 240.98 | 229.94 | 0.0082 | 0.0463 | Downregulated |
| hsa-miR-3945 | 49.30 | 46.76 | 0.0082 | 0.0463 | Downregulated |
| hsa-miR-4688 | 41.32 | 39.75 | 0.0083 | 0.0463 | Downregulated |
| hsa-miR-4743-5p | 57.98 | 55.80 | 0.0081 | 0.0463 | Downregulated |
| hsa-miR-5088 | 56.38 | 53.76 | 0.0081 | 0.0463 | Downregulated |
| hsa-miR-548q | 87.37 | 81.08 | 0.0079 | 0.0463 | Downregulated |
| hsa-miR-6127 | 1501.69 | 1417.19 | 0.0080 | 0.0463 | Downregulated |
| hsa-miR-769-3p | 23.64 | 22.59 | 0.0081 | 0.0463 | Downregulated |
| hsa-miR-149-3p | 34.86 | 32.71 | 0.0088 | 0.0484 | Downregulated |
| hsa-miR-4327 | 240.78 | 228.32 | 0.0088 | 0.0484 | Downregulated |
| hsa-miR-4270 | 477.02 | 456.52 | 0.0090 | 0.0489 | Downregulated |
| hsa-miR-4497 | 3647.25 | 3475.82 | 0.0090 | 0.0489 | Downregulated |
| hsa-let-7e-5p | 15.53 | 17.70 | 0.0091 | 0.0491 | Upregulated |
| hsa-miR-3194-5p | 161.47 | 148.78 | 0.0092 | 0.0493 | Downregulated |
| hsa-miR-1208 | 32.90 | 31.48 | 0.0096 | 0.0495 | Downregulated |
| hsa-miR-30a-5p | 2.04 | 2.63 | 0.0095 | 0.0495 | Upregulated |
| hsa-miR-3656 | 2942.27 | 2730.94 | 0.0094 | 0.0495 | Downregulated |
| hsa-miR-4792 | 27.45 | 26.18 | 0.0096 | 0.0495 | Downregulated |
| hsa-miR-6068 | 2659.00 | 2504.12 | 0.0096 | 0.0495 | Downregulated |
| hsa-miR-622 | 92.97 | 87.38 | 0.0093 | 0.0495 | Downregulated |
| hsa-miR-107 | 25.08 | 28.02 | 0.0098 | 0.0496 | Upregulated |
| hsa-miR-3665 | 3566.93 | 3351.19 | 0.0097 | 0.0496 | Downregulated |
| hsa-miR-718 | 115.04 | 110.33 | 0.0098 | 0.0496 | Downregulated |
| hsa-miR-30c-1-3p | 11.53 | 10.96 | 0.0099 | 0.0498 | Downregulated |
| hsa-miR-1183 | 64.05 | 61.61 | 0.0100 | 0.0500 | Downregulated |
| hsa-miR-1207-5p | 2047.51 | 1952.97 | 0.0104 | 0.0501 | Downregulated |
| hsa-miR-192-5p | 113.40 | 125.48 | 0.0101 | 0.0501 | Upregulated |
| hsa-miR-3173-3p | 7.77 | 8.13 | 0.0104 | 0.0501 | Upregulated |
| hsa-miR-3925-5p | 45.30 | 43.60 | 0.0105 | 0.0501 | Downregulated |
| hsa-miR-4417 | 186.10 | 177.56 | 0.0103 | 0.0501 | Downregulated |
| hsa-miR-4419b | 27.06 | 25.81 | 0.0103 | 0.0501 | Downregulated |
| hsa-miR-4508 | 59.03 | 56.54 | 0.0105 | 0.0501 | Downregulated |
| hsa-miR-532-3p | 0.43 | 0.66 | 0.0104 | 0.0501 | Upregulated |
| hsa-miR-4433-3p | 291.83 | 270.02 | 0.0106 | 0.0502 | Downregulated |
| hsa-miR-34a-5p | 9.51 | 10.49 | 0.0109 | 0.0511 | Upregulated |
| hsa-miR-3610 | 132.81 | 124.99 | 0.0109 | 0.0511 | Downregulated |
| hsa-miR-3692-5p | 58.88 | 55.56 | 0.0110 | 0.0512 | Downregulated |
| hsa-let-7g-5p | 41.86 | 46.85 | 0.0114 | 0.0516 | Upregulated |
| hsa-miR-1307-3p | 12.82 | 12.25 | 0.0117 | 0.0516 | Downregulated |
| hsa-miR-181b-5p | 17.61 | 16.94 | 0.0112 | 0.0516 | Downregulated |
| hsa-miR-200a-5p | 4.30 | 4.72 | 0.0116 | 0.0516 | Upregulated |
| hsa-miR-215 | 58.60 | 64.98 | 0.0117 | 0.0516 | Upregulated |
| hsa-miR-29c-3p | 13.42 | 15.40 | 0.0115 | 0.0516 | Upregulated |
| hsa-miR-365a-3p | 2.05 | 2.77 | 0.0117 | 0.0516 | Upregulated |
| hsa-miR-3911 | 85.54 | 82.07 | 0.0115 | 0.0516 | Downregulated |
| hsa-miR-4514 | 25.26 | 23.84 | 0.0117 | 0.0516 | Downregulated |
| hsa-miR-5739 | 1328.67 | 1280.10 | 0.0114 | 0.0516 | Downregulated |
| hsa-miR-4659a-3p | 29.33 | 27.85 | 0.0118 | 0.0517 | Downregulated |
| hsa-miR-151b | 3.74 | 4.35 | 0.0121 | 0.0522 | Upregulated |
| hsa-miR-371a-5p | 227.37 | 217.32 | 0.0120 | 0.0522 | Downregulated |
| hsa-miR-770-5p | 5.32 | 5.63 | 0.0121 | 0.0522 | Upregulated |
| hsa-miR-4430 | 1498.25 | 1429.95 | 0.0122 | 0.0524 | Downregulated |
| hsa-miR-514b-5p | 31.19 | 29.88 | 0.0123 | 0.0525 | Downregulated |
| hsa-miR-5703 | 321.19 | 313.61 | 0.0125 | 0.0531 | Downregulated |
| hsa-miR-4532 | 394.33 | 372.54 | 0.0126 | 0.0533 | Downregulated |
| hsa-miR-3141 | 201.70 | 191.75 | 0.0127 | 0.0534 | Downregulated |
| hsa-miR-342-3p | 14.68 | 16.09 | 0.0130 | 0.0541 | Upregulated |
| hsa-miR-4741 | 1251.79 | 1187.30 | 0.0130 | 0.0541 | Downregulated |
| hsa-miR-1254 | 20.76 | 19.90 | 0.0132 | 0.0547 | Downregulated |
| hsa-miR-106b-5p | 3.27 | 4.04 | 0.0135 | 0.0549 | Upregulated |
| hsa-miR-200c-3p | 99.58 | 111.09 | 0.0138 | 0.0549 | Upregulated |
| hsa-miR-2861 | 6405.29 | 6048.59 | 0.0135 | 0.0549 | Downregulated |
| hsa-miR-373-5p | 17.69 | 16.69 | 0.0134 | 0.0549 | Downregulated |
| hsa-miR-4304 | 17.60 | 17.00 | 0.0137 | 0.0549 | Downregulated |
| hsa-miR-4421 | 1.11 | 1.38 | 0.0140 | 0.0549 | Upregulated |
| hsa-miR-4453 | 76.23 | 70.44 | 0.0139 | 0.0549 | Downregulated |
| hsa-miR-4672 | 938.01 | 897.18 | 0.0136 | 0.0549 | Downregulated |
| hsa-miR-483-5p | 100.99 | 97.01 | 0.0140 | 0.0549 | Downregulated |
| hsa-miR-520b | 20.39 | 19.37 | 0.0139 | 0.0549 | Downregulated |
| hsa-miR-662 | 42.93 | 41.54 | 0.0137 | 0.0549 | Downregulated |
| hsa-miR-10b-5p | 12.69 | 14.87 | 0.0142 | 0.0554 | Upregulated |
| hsa-miR-1182 | 23.38 | 22.27 | 0.0143 | 0.0556 | Downregulated |
| hsa-miR-320c | 190.90 | 186.69 | 0.0145 | 0.0557 | Downregulated |
| hsa-miR-4701-3p | 77.29 | 72.44 | 0.0146 | 0.0557 | Downregulated |
| hsa-miR-498 | 27.07 | 25.43 | 0.0146 | 0.0557 | Downregulated |
| hsa-miR-5190 | 29.35 | 28.46 | 0.0145 | 0.0557 | Downregulated |
| hsa-miR-130a-3p | 2.38 | 3.12 | 0.0152 | 0.0561 | Upregulated |
| hsa-miR-133b | 3.30 | 4.14 | 0.0150 | 0.0561 | Upregulated |
| hsa-miR-15a-5p | 2.61 | 3.33 | 0.0149 | 0.0561 | Upregulated |
| hsa-miR-200b-3p | 113.17 | 126.77 | 0.0154 | 0.0561 | Upregulated |
| hsa-miR-27a-3p | 17.01 | 18.90 | 0.0152 | 0.0561 | Upregulated |
| hsa-miR-4513 | 28.82 | 27.83 | 0.0153 | 0.0561 | Downregulated |
| hsa-miR-4530 | 7705.79 | 7333.08 | 0.0148 | 0.0561 | Downregulated |
| hsa-miR-4763-3p | 1391.43 | 1323.85 | 0.0153 | 0.0561 | Downregulated |
| hsa-miR-5194 | 63.76 | 60.08 | 0.0152 | 0.0561 | Downregulated |
| hsa-miR-575 | 626.23 | 595.07 | 0.0154 | 0.0561 | Downregulated |
| hsa-miR-4496 | 60.10 | 57.82 | 0.0161 | 0.0584 | Downregulated |
| hsa-miR-23b-3p | 57.02 | 63.83 | 0.0162 | 0.0585 | Upregulated |
| hsa-miR-3162-5p | 2914.75 | 2738.08 | 0.0165 | 0.0591 | Downregulated |
| hsa-miR-3940-5p | 824.05 | 784.03 | 0.0165 | 0.0591 | Downregulated |
| hsa-miR-6088 | 3327.60 | 3137.15 | 0.0166 | 0.0592 | Downregulated |
| hsa-miR-4488 | 118.28 | 111.14 | 0.0171 | 0.0605 | Downregulated |
| hsa-miR-630 | 467.66 | 456.02 | 0.0172 | 0.0605 | Downregulated |
| hsa-miR-936 | 52.84 | 50.92 | 0.0172 | 0.0605 | Downregulated |
| hsa-miR-4640-5p | 28.85 | 27.95 | 0.0177 | 0.0620 | Downregulated |
| hsa-miR-3652 | 159.87 | 153.15 | 0.0178 | 0.0621 | Downregulated |
| hsa-miR-6090 | 6344.29 | 6123.43 | 0.0179 | 0.0622 | Downregulated |
| hsa-miR-429 | 4.64 | 5.68 | 0.0181 | 0.0626 | Upregulated |
| hsa-miR-6510-5p | 288.53 | 269.20 | 0.0182 | 0.0627 | Downregulated |
| hsa-miR-4690-5p | 210.65 | 199.02 | 0.0183 | 0.0628 | Downregulated |
| hsa-miR-1185-2-3p | 141.70 | 134.37 | 0.0188 | 0.0634 | Downregulated |
| hsa-miR-1915-3p | 3427.19 | 3278.02 | 0.0188 | 0.0634 | Downregulated |
| hsa-miR-5006-5p | 729.49 | 701.39 | 0.0186 | 0.0634 | Downregulated |
| hsa-miR-6125 | 10900.18 | 10317.19 | 0.0187 | 0.0634 | Downregulated |
| hsa-miR-196a-5p | 2.41 | 3.05 | 0.0193 | 0.0640 | Upregulated |
| hsa-miR-20b-5p | 1.94 | 2.59 | 0.0192 | 0.0640 | Upregulated |
| hsa-miR-29a-3p | 37.52 | 42.06 | 0.0194 | 0.0640 | Upregulated |
| hsa-miR-29b-3p | 6.84 | 8.00 | 0.0193 | 0.0640 | Upregulated |
| hsa-miR-3174 | 11.87 | 12.43 | 0.0195 | 0.0640 | Upregulated |
| hsa-miR-3682-3p | 91.55 | 88.72 | 0.0193 | 0.0640 | Downregulated |
| hsa-miR-425-5p | 4.64 | 5.44 | 0.0196 | 0.0640 | Upregulated |
| hsa-miR-6131 | 157.59 | 148.79 | 0.0196 | 0.0640 | Downregulated |
| hsa-miR-1234-5p | 5138.31 | 4885.10 | 0.0199 | 0.0647 | Downregulated |
| hsa-miR-146b-5p | 0.82 | 1.19 | 0.0207 | 0.0671 | Upregulated |
| hsa-miR-1275 | 786.60 | 732.96 | 0.0209 | 0.0672 | Downregulated |
| hsa-miR-188-5p | 453.93 | 431.59 | 0.0209 | 0.0672 | Downregulated |
| hsa-miR-4745-5p | 286.40 | 274.20 | 0.0212 | 0.0679 | Downregulated |
| hsa-let-7i-5p | 31.72 | 34.42 | 0.0213 | 0.0680 | Upregulated |
| hsa-miR-3198 | 182.01 | 174.24 | 0.0218 | 0.0686 | Downregulated |
| hsa-miR-378d | 0.46 | 0.63 | 0.0217 | 0.0686 | Upregulated |
| hsa-miR-378f | 10.13 | 10.34 | 0.0221 | 0.0686 | Upregulated |
| hsa-miR-4298 | 165.06 | 157.79 | 0.0219 | 0.0686 | Downregulated |
| hsa-miR-4516 | 17739.82 | 17120.55 | 0.0219 | 0.0686 | Downregulated |
| hsa-miR-4651 | 229.68 | 219.52 | 0.0221 | 0.0686 | Downregulated |
| hsa-miR-490-5p | 21.17 | 20.29 | 0.0220 | 0.0686 | Downregulated |
| hsa-miR-150-3p | 271.13 | 260.97 | 0.0223 | 0.0687 | Downregulated |
| hsa-miR-371b-5p | 1447.86 | 1358.48 | 0.0222 | 0.0687 | Downregulated |
| hsa-miR-1185-1-3p | 423.02 | 403.44 | 0.0226 | 0.0694 | Downregulated |
| hsa-miR-6076 | 264.22 | 253.52 | 0.0228 | 0.0698 | Downregulated |
| hsa-miR-4531 | 10.18 | 10.48 | 0.0233 | 0.0710 | Upregulated |
| hsa-miR-3137 | 236.64 | 225.58 | 0.0239 | 0.0723 | Downregulated |
| hsa-miR-3648 | 282.05 | 269.88 | 0.0239 | 0.0723 | Downregulated |
| hsa-miR-4664-3p | 34.68 | 33.46 | 0.0243 | 0.0730 | Downregulated |
| hsa-miR-4793-5p | 217.76 | 212.18 | 0.0243 | 0.0730 | Downregulated |
| hsa-miR-4478 | 325.53 | 315.49 | 0.0245 | 0.0733 | Downregulated |
| hsa-miR-4281 | 3909.82 | 3721.03 | 0.0249 | 0.0737 | Downregulated |
| hsa-miR-4776-5p | 36.80 | 35.74 | 0.0249 | 0.0737 | Downregulated |
| hsa-miR-484 | 0.53 | 0.78 | 0.0249 | 0.0737 | Upregulated |
| hsa-miR-6129 | 88.24 | 82.02 | 0.0254 | 0.0749 | Downregulated |
| hsa-miR-6087 | 17370.94 | 16701.52 | 0.0263 | 0.0770 | Downregulated |
| hsa-miR-6723-5p | 132.00 | 128.48 | 0.0262 | 0.0770 | Downregulated |
| hsa-miR-3667-5p | 24.59 | 23.93 | 0.0269 | 0.0785 | Downregulated |
| hsa-miR-939-5p | 665.00 | 630.21 | 0.0271 | 0.0788 | Downregulated |
| hsa-let-7f-5p | 99.16 | 112.23 | 0.0274 | 0.0791 | Upregulated |
| hsa-miR-1228-5p | 4.77 | 5.05 | 0.0275 | 0.0791 | Upregulated |
| hsa-miR-1305 | 139.66 | 132.27 | 0.0273 | 0.0791 | Downregulated |
| hsa-miR-6089 | 32876.04 | 31165.56 | 0.0277 | 0.0794 | Downregulated |
| hsa-miR-197-5p | 3286.45 | 3105.19 | 0.0292 | 0.0834 | Downregulated |
| hsa-miR-103a-3p | 39.33 | 43.62 | 0.0296 | 0.0837 | Upregulated |
| hsa-miR-1268b | 1198.55 | 1133.71 | 0.0296 | 0.0837 | Downregulated |
| hsa-miR-4257 | 358.98 | 338.22 | 0.0296 | 0.0837 | Downregulated |
| hsa-miR-10b-3p | 50.93 | 49.24 | 0.0301 | 0.0848 | Downregulated |
| hsa-miR-3200-5p | 29.68 | 28.66 | 0.0304 | 0.0854 | Downregulated |
| hsa-miR-192-3p | 1.23 | 1.63 | 0.0308 | 0.0859 | Upregulated |
| hsa-miR-6717-5p | 144.81 | 137.07 | 0.0307 | 0.0859 | Downregulated |
| hsa-miR-566 | 3.07 | 3.36 | 0.0315 | 0.0876 | Upregulated |
| hsa-miR-4697-5p | 115.83 | 109.68 | 0.0318 | 0.0881 | Downregulated |
| hsa-miR-5090 | 13.11 | 12.28 | 0.0319 | 0.0881 | Downregulated |
| hsa-miR-135a-3p | 164.63 | 156.33 | 0.0323 | 0.0886 | Downregulated |
| hsa-miR-4459 | 25685.12 | 24185.73 | 0.0324 | 0.0886 | Downregulated |
| hsa-miR-4472 | 8.90 | 8.28 | 0.0323 | 0.0886 | Downregulated |
| hsa-miR-1225-5p | 2984.00 | 2836.77 | 0.0330 | 0.0893 | Downregulated |
| hsa-miR-148a-3p | 8.84 | 9.98 | 0.0328 | 0.0893 | Upregulated |
| hsa-miR-762 | 2088.02 | 2009.06 | 0.0330 | 0.0893 | Downregulated |
| hsa-let-7a-5p | 191.79 | 215.76 | 0.0336 | 0.0894 | Upregulated |
| hsa-miR-1224-5p | 962.98 | 923.94 | 0.0336 | 0.0894 | Downregulated |
| hsa-miR-4324 | 1.10 | 1.24 | 0.0336 | 0.0894 | Upregulated |
| hsa-miR-4698 | 37.22 | 35.83 | 0.0335 | 0.0894 | Downregulated |
| hsa-miR-5581-5p | 128.22 | 123.12 | 0.0335 | 0.0894 | Downregulated |
| SMAD3 (rs7176870) | AA ( N=332 ) | AG/GG ( N=778 ) |  |  |  |
| hsa-miR-1181 | 243.69 | 228.27 | 0.0009 | 0.0820 | Downregulated |
| hsa-miR-345-5p | 63.93 | 60.90 | 0.0009 | 0.0820 | Downregulated |
| hsa-miR-3925-5p | 45.88 | 43.42 | 0.0008 | 0.0820 | Downregulated |
| hsa-miR-4451 | 15.78 | 14.69 | 0.0006 | 0.0820 | Downregulated |
| hsa-miR-4538 | 187.31 | 176.31 | 0.0002 | 0.0820 | Downregulated |
| hsa-miR-4632-5p | 220.85 | 208.18 | 0.0007 | 0.0820 | Downregulated |
| hsa-miR-4733-5p | 70.37 | 66.38 | 0.0009 | 0.0820 | Downregulated |
| hsa-miR-4758-5p | 131.99 | 120.67 | 0.0005 | 0.0820 | Downregulated |
| hsa-miR-4776-5p | 37.15 | 35.62 | 0.0003 | 0.0820 | Downregulated |
| ***RUNX1*** (rs8134179) | TT ( N=793 ) | TC/CC ( N=317 ) |  |  |  |
| hsa-miR-138-2-3p | 6.29 | 7.08 | 0.0003 | 0.0469 | Upregulated |
| hsa-miR-3177-5p | 4.23 | 4.85 | 0.0004 | 0.0469 | Upregulated |
| hsa-miR-3614-5p | 4.96 | 5.95 | <.0001 | 0.0469 | Upregulated |
| hsa-miR-4461 | 3.36 | 4.14 | 0.0003 | 0.0469 | Upregulated |
| hsa-miR-4519 | 7.77 | 8.67 | 0.0003 | 0.0469 | Upregulated |
| hsa-miR-5696 | 6.03 | 7.10 | 0.0004 | 0.0469 | Upregulated |
| hsa-miR-658 | 0.64 | 0.80 | 0.0002 | 0.0469 | Upregulated |
| hsa-miR-4793-3p | 5.24 | 6.26 | 0.0006 | 0.0547 | Upregulated |
| hsa-miR-5585-5p | 4.32 | 5.06 | 0.0006 | 0.0547 | Upregulated |
| hsa-miR-1203 | 1.79 | 2.11 | 0.0012 | 0.0703 | Upregulated |
| hsa-miR-3124-5p | 1.14 | 1.47 | 0.0010 | 0.0703 | Upregulated |
| hsa-miR-508-5p | 3.30 | 4.04 | 0.0011 | 0.0703 | Upregulated |
| hsa-miR-526b-5p | 3.36 | 3.81 | 0.0009 | 0.0703 | Upregulated |
| hsa-miR-550a-5p | 7.77 | 8.40 | 0.0012 | 0.0703 | Upregulated |
| hsa-miR-4659b-3p | 4.03 | 4.67 | 0.0014 | 0.0717 | Upregulated |
| hsa-miR-516b-5p | 4.58 | 5.21 | 0.0014 | 0.0717 | Upregulated |
| hsa-miR-4316 | 5.37 | 6.17 | 0.0016 | 0.0772 | Upregulated |
| hsa-miR-583 | 3.20 | 3.80 | 0.0017 | 0.0774 | Upregulated |
| hsa-miR-2110 | 0.42 | 0.58 | 0.0018 | 0.0777 | Upregulated |
| hsa-miR-5194 | 62.58 | 57.93 | 0.0020 | 0.0820 | Downregulated |

| Supplemental Table 2. SNPs in TGFBeta signaling pathway associated with miRNAs in normal colonic mucosa; FDR<0.09 | | | | | |
| --- | --- | --- | --- | --- | --- |
| miRNA | Mean | Mean | P-values | FDR adjusted P | Direction |
| ***RUNX2*** (rs12333172) | CC/CT ( N=626 ) | TT ( N=36 ) |  |  |  |
| hsa-miR-1226-5p | 54.59 | 79.49 | 0.0015 | 0.0717 | Upregulated |
| hsa-miR-26a-5p | 82.50 | 64.46 | 0.0009 | 0.0717 | Downregulated |
| hsa-miR-30c-1-3p | 11.15 | 15.53 | 0.0012 | 0.0717 | Upregulated |
| hsa-miR-3621 | 37.96 | 53.40 | 0.0014 | 0.0717 | Upregulated |
| hsa-miR-3663-3p | 167.22 | 252.07 | 0.0015 | 0.0717 | Upregulated |
| hsa-miR-3960 | 10454.46 | 20170.24 | 0.0015 | 0.0717 | Upregulated |
| hsa-miR-4316 | 6.93 | 4.87 | 0.0014 | 0.0717 | Downregulated |
| hsa-miR-4498 | 63.88 | 56.98 | 0.0002 | 0.0717 | Downregulated |
| hsa-miR-4516 | 17558.75 | 31136.08 | 0.0015 | 0.0717 | Upregulated |
| hsa-miR-4634 | 326.83 | 612.64 | 0.0013 | 0.0717 | Upregulated |
| hsa-miR-4701-3p | 75.06 | 109.36 | 0.0003 | 0.0717 | Upregulated |
| hsa-miR-4783-3p | 16.70 | 23.43 | 0.0013 | 0.0717 | Upregulated |
| hsa-miR-5739 | 1304.71 | 1856.95 | 0.0012 | 0.0717 | Upregulated |
| hsa-miR-628-3p | 18.91 | 17.63 | 0.0010 | 0.0717 | Downregulated |
| hsa-miR-6723-5p | 135.57 | 197.55 | 0.0009 | 0.0717 | Upregulated |
| hsa-miR-758-5p | 32.86 | 30.14 | 0.0008 | 0.0717 | Downregulated |
| hsa-miR-921 | 21.74 | 18.66 | 0.0012 | 0.0717 | Downregulated |
| hsa-miR-1183 | 64.03 | 79.17 | 0.0044 | 0.0723 | Upregulated |
| hsa-miR-1202 | 1438.89 | 2337.70 | 0.0069 | 0.0723 | Upregulated |
| hsa-miR-1207-5p | 2040.66 | 3497.26 | 0.0039 | 0.0723 | Upregulated |
| hsa-miR-1225-3p | 18.19 | 26.06 | 0.0043 | 0.0723 | Upregulated |
| hsa-miR-1247-3p | 26.09 | 32.26 | 0.0037 | 0.0723 | Upregulated |
| hsa-miR-126-3p | 13.78 | 10.00 | 0.0057 | 0.0723 | Downregulated |
| hsa-miR-1275 | 756.28 | 1346.75 | 0.0038 | 0.0723 | Upregulated |
| hsa-miR-129-5p | 9.83 | 14.39 | 0.0040 | 0.0723 | Upregulated |
| hsa-miR-1307-3p | 12.71 | 15.97 | 0.0047 | 0.0723 | Upregulated |
| hsa-miR-134 | 285.56 | 470.82 | 0.0034 | 0.0723 | Upregulated |
| hsa-miR-139-3p | 12.03 | 15.12 | 0.0062 | 0.0723 | Upregulated |
| hsa-miR-146b-5p | 1.27 | 0.25 | 0.0022 | 0.0723 | Downregulated |
| hsa-miR-187-5p | 18.86 | 34.90 | 0.0028 | 0.0723 | Upregulated |
| hsa-miR-1915-3p | 3424.83 | 5479.73 | 0.0062 | 0.0723 | Upregulated |
| hsa-miR-192-5p | 124.60 | 105.26 | 0.0057 | 0.0723 | Downregulated |
| hsa-miR-194-5p | 108.48 | 92.04 | 0.0028 | 0.0723 | Downregulated |
| hsa-miR-200c-3p | 104.48 | 92.68 | 0.0058 | 0.0723 | Downregulated |
| hsa-miR-211-3p | 63.89 | 87.91 | 0.0046 | 0.0723 | Upregulated |
| hsa-miR-214-3p | 4.76 | 3.37 | 0.0060 | 0.0723 | Downregulated |
| hsa-miR-24-3p | 52.34 | 46.70 | 0.0030 | 0.0723 | Downregulated |
| hsa-miR-26b-5p | 17.40 | 12.61 | 0.0058 | 0.0723 | Downregulated |
| hsa-miR-3131 | 16.83 | 37.51 | 0.0056 | 0.0723 | Upregulated |
| hsa-miR-3138 | 80.54 | 127.33 | 0.0047 | 0.0723 | Upregulated |
| hsa-miR-3185 | 17.52 | 25.05 | 0.0052 | 0.0723 | Upregulated |
| hsa-miR-3195 | 1059.65 | 1988.34 | 0.0030 | 0.0723 | Upregulated |
| hsa-miR-3197 | 30.82 | 38.26 | 0.0043 | 0.0723 | Upregulated |
| hsa-miR-320c | 173.01 | 555.27 | 0.0046 | 0.0723 | Upregulated |
| hsa-miR-345-3p | 60.14 | 98.51 | 0.0058 | 0.0723 | Upregulated |
| hsa-miR-3667-5p | 22.79 | 57.09 | 0.0027 | 0.0723 | Upregulated |
| hsa-miR-378a-3p | 146.57 | 134.82 | 0.0042 | 0.0723 | Downregulated |
| hsa-miR-378d | 0.62 | 0.14 | 0.0071 | 0.0723 | Downregulated |
| hsa-miR-378i | 72.84 | 66.99 | 0.0027 | 0.0723 | Downregulated |
| hsa-miR-3945 | 47.69 | 70.74 | 0.0023 | 0.0723 | Upregulated |
| hsa-miR-4253 | 48.01 | 59.19 | 0.0039 | 0.0723 | Upregulated |
| hsa-miR-4315 | 0.64 | 0.08 | 0.0067 | 0.0723 | Downregulated |
| hsa-miR-4508 | 57.50 | 81.15 | 0.0041 | 0.0723 | Upregulated |
| hsa-miR-4513 | 29.24 | 36.13 | 0.0072 | 0.0723 | Upregulated |
| hsa-miR-4534 | 255.35 | 436.18 | 0.0027 | 0.0723 | Upregulated |
| hsa-miR-4657 | 2.16 | 1.14 | 0.0049 | 0.0723 | Downregulated |
| hsa-miR-4664-3p | 34.49 | 44.65 | 0.0052 | 0.0723 | Upregulated |
| hsa-miR-4665-5p | 61.27 | 77.74 | 0.0020 | 0.0723 | Upregulated |
| hsa-miR-4689 | 127.10 | 199.44 | 0.0069 | 0.0723 | Upregulated |
| hsa-miR-4739 | 1027.30 | 1560.24 | 0.0062 | 0.0723 | Upregulated |
| hsa-miR-4741 | 1271.30 | 1735.60 | 0.0060 | 0.0723 | Upregulated |
| hsa-miR-4745-5p | 288.96 | 403.41 | 0.0055 | 0.0723 | Upregulated |
| hsa-miR-493-3p | 2.70 | 1.87 | 0.0069 | 0.0723 | Downregulated |
| hsa-miR-5006-5p | 738.00 | 941.70 | 0.0059 | 0.0723 | Upregulated |
| hsa-miR-5703 | 270.52 | 1071.90 | 0.0046 | 0.0723 | Upregulated |
| hsa-miR-572 | 509.16 | 825.38 | 0.0071 | 0.0723 | Upregulated |
| hsa-miR-5787 | 2096.90 | 5640.36 | 0.0016 | 0.0723 | Upregulated |
| hsa-miR-601 | 46.81 | 68.28 | 0.0056 | 0.0723 | Upregulated |
| hsa-miR-6069 | 18.84 | 27.24 | 0.0067 | 0.0723 | Upregulated |
| hsa-miR-6087 | 17312.26 | 26452.71 | 0.0065 | 0.0723 | Upregulated |
| hsa-miR-6089 | 32507.61 | 49717.23 | 0.0067 | 0.0723 | Upregulated |
| hsa-miR-6090 | 6308.64 | 13423.72 | 0.0036 | 0.0723 | Upregulated |
| hsa-miR-622 | 87.82 | 131.99 | 0.0056 | 0.0723 | Upregulated |
| hsa-miR-623 | 48.44 | 66.53 | 0.0034 | 0.0723 | Upregulated |
| hsa-miR-630 | 398.26 | 1592.15 | 0.0026 | 0.0723 | Upregulated |
| hsa-miR-638 | 4161.19 | 6327.05 | 0.0070 | 0.0723 | Upregulated |
| hsa-miR-6500-5p | 19.04 | 24.47 | 0.0023 | 0.0723 | Upregulated |
| hsa-miR-6510-5p | 275.04 | 489.33 | 0.0070 | 0.0723 | Upregulated |
| hsa-miR-718 | 114.87 | 182.74 | 0.0024 | 0.0723 | Upregulated |
| hsa-miR-762 | 2116.24 | 2890.47 | 0.0055 | 0.0723 | Upregulated |
| hsa-miR-99b-3p | 59.95 | 69.48 | 0.0072 | 0.0723 | Upregulated |
| hsa-miR-141-3p | 28.69 | 22.80 | 0.0074 | 0.0725 | Downregulated |
| hsa-miR-3121-3p | 5.99 | 4.40 | 0.0074 | 0.0725 | Downregulated |
| hsa-miR-1228-3p | 34.35 | 48.70 | 0.0078 | 0.0731 | Upregulated |
| hsa-miR-2276 | 95.76 | 131.31 | 0.0076 | 0.0731 | Upregulated |
| hsa-miR-4270 | 480.91 | 846.12 | 0.0080 | 0.0731 | Upregulated |
| hsa-miR-4787-3p | 110.39 | 174.43 | 0.0079 | 0.0731 | Upregulated |
| hsa-miR-513b | 209.71 | 201.22 | 0.0080 | 0.0731 | Downregulated |
| hsa-miR-6068 | 2661.92 | 4240.78 | 0.0079 | 0.0731 | Upregulated |
| hsa-miR-5088 | 55.39 | 72.25 | 0.0087 | 0.0786 | Upregulated |
| hsa-miR-5684 | 38.35 | 36.82 | 0.0088 | 0.0786 | Downregulated |
| hsa-miR-30a-5p | 2.64 | 1.25 | 0.0099 | 0.0797 | Downregulated |
| hsa-miR-30b-5p | 18.19 | 14.07 | 0.0098 | 0.0797 | Downregulated |
| hsa-miR-3174 | 13.64 | 11.86 | 0.0096 | 0.0797 | Downregulated |
| hsa-miR-3620-5p | 97.47 | 131.01 | 0.0098 | 0.0797 | Upregulated |
| hsa-miR-431-5p | 54.67 | 50.09 | 0.0095 | 0.0797 | Upregulated |
| hsa-miR-4433-5p | 19.47 | 28.46 | 0.0093 | 0.0797 | Upregulated |
| hsa-miR-4725-5p | 11.03 | 14.82 | 0.0098 | 0.0797 | Upregulated |
| hsa-miR-5003-3p | 20.28 | 26.57 | 0.0098 | 0.0797 | Upregulated |
| hsa-miR-510 | 0.39 | 0.08 | 0.0093 | 0.0797 | Downregulated |
| hsa-miR-769-3p | 24.87 | 32.77 | 0.0094 | 0.0797 | Upregulated |
| hsa-miR-3935 | 16.66 | 16.15 | 0.0101 | 0.0805 | Downregulated |
| hsa-miR-1261 | 3.76 | 2.89 | 0.0111 | 0.0813 | Downregulated |
| hsa-miR-1266 | 1.38 | 0.62 | 0.0112 | 0.0813 | Downregulated |
| hsa-miR-135a-3p | 160.07 | 227.59 | 0.0103 | 0.0813 | Upregulated |
| hsa-miR-4429 | 25.42 | 36.16 | 0.0111 | 0.0813 | Upregulated |
| hsa-miR-4459 | 24244.04 | 42288.88 | 0.0105 | 0.0813 | Upregulated |
| hsa-miR-4468 | 18.36 | 16.73 | 0.0106 | 0.0813 | Downregulated |
| hsa-miR-4476 | 73.91 | 113.75 | 0.0108 | 0.0813 | Upregulated |
| hsa-miR-483-5p | 98.57 | 157.26 | 0.0109 | 0.0813 | Upregulated |
| hsa-miR-498 | 27.73 | 38.34 | 0.0112 | 0.0813 | Upregulated |
| hsa-miR-6085 | 695.59 | 1008.69 | 0.0110 | 0.0813 | Upregulated |
| hsa-miR-151a-3p | 0.75 | 0.15 | 0.0125 | 0.0837 | Downregulated |
| hsa-miR-200b-3p | 121.72 | 108.22 | 0.0136 | 0.0837 | Downregulated |
| hsa-miR-215 | 64.82 | 53.61 | 0.0118 | 0.0837 | Downregulated |
| hsa-miR-22-3p | 14.67 | 12.04 | 0.0127 | 0.0837 | Downregulated |
| hsa-miR-298 | 27.98 | 32.76 | 0.0122 | 0.0837 | Upregulated |
| hsa-miR-3180-3p | 17.28 | 27.80 | 0.0121 | 0.0837 | Upregulated |
| hsa-miR-3926 | 33.56 | 32.18 | 0.0136 | 0.0837 | Downregulated |
| hsa-miR-3937 | 71.62 | 97.68 | 0.0140 | 0.0837 | Upregulated |
| hsa-miR-4280 | 3.14 | 2.00 | 0.0121 | 0.0837 | Downregulated |
| hsa-miR-4311 | 2.18 | 1.36 | 0.0140 | 0.0837 | Downregulated |
| hsa-miR-4466 | 1931.91 | 3272.18 | 0.0138 | 0.0837 | Upregulated |
| hsa-miR-4486 | 185.59 | 241.37 | 0.0125 | 0.0837 | Upregulated |
| hsa-miR-4532 | 365.21 | 603.11 | 0.0135 | 0.0837 | Upregulated |
| hsa-miR-4632-5p | 224.01 | 275.44 | 0.0135 | 0.0837 | Upregulated |
| hsa-miR-4655-3p | 25.19 | 37.72 | 0.0129 | 0.0837 | Upregulated |
| hsa-miR-4685-5p | 23.93 | 29.61 | 0.0130 | 0.0837 | Upregulated |
| hsa-miR-4700-5p | 0.40 | 0.10 | 0.0135 | 0.0837 | Downregulated |
| hsa-miR-4763-3p | 1392.44 | 2137.15 | 0.0139 | 0.0837 | Upregulated |
| hsa-miR-4767 | 28.09 | 40.27 | 0.0135 | 0.0837 | Upregulated |
| hsa-miR-4800-5p | 164.30 | 230.32 | 0.0138 | 0.0837 | Upregulated |
| hsa-miR-6086 | 358.38 | 524.26 | 0.0128 | 0.0837 | Upregulated |
| hsa-miR-6125 | 10660.42 | 16524.07 | 0.0131 | 0.0837 | Upregulated |
| hsa-miR-6165 | 323.37 | 510.42 | 0.0128 | 0.0837 | Upregulated |
| hsa-miR-874 | 220.57 | 282.91 | 0.0128 | 0.0837 | Upregulated |
| hsa-miR-125a-3p | 242.69 | 315.92 | 0.0144 | 0.0848 | Upregulated |
| hsa-miR-2861 | 6433.29 | 9627.03 | 0.0147 | 0.0848 | Upregulated |
| hsa-miR-500a-3p | 2.57 | 1.82 | 0.0143 | 0.0848 | Downregulated |
| hsa-miR-6126 | 692.83 | 1050.09 | 0.0145 | 0.0848 | Upregulated |
| hsa-miR-939-5p | 681.60 | 898.39 | 0.0147 | 0.0848 | Upregulated |
| hsa-miR-3158-5p | 32.55 | 49.98 | 0.0155 | 0.0851 | Upregulated |
| hsa-miR-4296 | 0.77 | 0.32 | 0.0151 | 0.0851 | Downregulated |
| hsa-miR-4442 | 292.53 | 385.52 | 0.0154 | 0.0851 | Upregulated |
| hsa-miR-4530 | 7510.04 | 9487.60 | 0.0152 | 0.0851 | Upregulated |
| hsa-miR-4655-5p | 34.60 | 44.09 | 0.0151 | 0.0851 | Upregulated |
| hsa-miR-4778-5p | 56.79 | 80.39 | 0.0153 | 0.0851 | Upregulated |
| hsa-miR-6722-3p | 102.86 | 167.24 | 0.0154 | 0.0851 | Upregulated |
| hsa-miR-518c-5p | 1.56 | 0.83 | 0.0160 | 0.0873 | Downregulated |
| hsa-miR-652-5p | 84.07 | 107.93 | 0.0162 | 0.0878 | Upregulated |
| hsa-miR-30c-5p | 8.18 | 6.09 | 0.0164 | 0.0882 | Downregulated |
| hsa-miR-3188 | 245.94 | 391.69 | 0.0166 | 0.0882 | Upregulated |
| hsa-miR-6124 | 516.40 | 774.76 | 0.0166 | 0.0882 | Upregulated |
| hsa-miR-4710 | 40.65 | 50.71 | 0.0168 | 0.0887 | Upregulated |
| ***EIF4E*** (rs12498533) | AA ( N=188 ) | AC/CC ( N=473 ) |  |  |  |
| hsa-miR-3180-3p | 16.08 | 18.65 | <.0001 | 0.0813 | Upregulated |
| ***BMPR1B*** (rs13134042) | GG/GA ( N=640 ) | AA ( N=22 ) |  |  |  |
| hsa-miR-10a-3p | 3.40 | 6.55 | <.0001 | 0.0813 | Upregulated |
| ***RUNX1*** (rs8134179) | TT ( N=463 ) | TC/CC ( N=199 ) |  |  |  |
| hsa-miR-526b-5p | 3.52 | 4.13 | <.0001 | 0.0406 | Upregulated |
| hsa-miR-658 | 0.72 | 0.95 | <.0001 | 0.0406 | Upregulated |
| hsa-miR-4659b-3p | 3.77 | 4.78 | 0.0002 | 0.0542 | Upregulated |
| hsa-miR-3187-5p | 2.03 | 2.74 | 0.0004 | 0.0813 | Upregulated |
| hsa-miR-34c-3p | 4.63 | 5.28 | 0.0006 | 0.0813 | Upregulated |
| hsa-miR-4722-5p | 3.39 | 4.13 | 0.0006 | 0.0813 | Upregulated |

| Supplemental Table 3. SNPs in TGFBeta-Signaling Pathway Associated with miRNA in normal rectal mucosa; FDR<0.09 | | | | | |
| --- | --- | --- | --- | --- | --- |
| miRNA | Mean | Mean | P-values | FDR adjusted P | Direction |
| ***SMAD3*** (rs12904944) | GG ( N=193 ) | GA/AA ( N=255 ) |  |  |  |
| hsa-miR-662 | 38.47 | 42.35 | <.0001 | 0.0825 | Upregulated |
| ***TGFBR1*** (rs1571590) | AA ( N=301 ) | AG/GG ( N=147 ) |  |  |  |
| hsa-miR-29b-3p | 7.59 | 6.19 | 0.0002 | 0.0825 | Downregulated |
| hsa-miR-29c-3p | 15.03 | 12.35 | 0.0002 | 0.0825 | Downregulated |
| ***EIF4EBP3*** (rs250425) | CC ( N=288 ) | CT/TT ( N=181 ) |  |  |  |
| hsa-miR-4715-5p | 1.79 | 2.68 | <.0001 | 0.0825 | Upregulated |
| ***SMAD7*** (rs3736242) | GG/GA ( N=423 ) | AA ( N=25 ) |  |  |  |
| hsa-miR-431-5p | 37.73 | 29.85 | <.0001 | 0.0825 | Downregulated |
| ***PTEN*** (rs532678) | CC ( N=167 ) | CT/TT ( N=281 ) |  |  |  |
| hsa-miR-10a-3p | 2.55 | 1.86 | 0.0002 | 0.0450 | Downregulated |
| hsa-miR-1203 | 2.03 | 1.55 | 0.0014 | 0.0450 | Downregulated |
| hsa-miR-1261 | 3.26 | 2.61 | 0.0012 | 0.0450 | Downregulated |
| hsa-miR-130b-3p | 3.22 | 2.47 | 0.0002 | 0.0450 | Downregulated |
| hsa-miR-1323 | 3.74 | 2.91 | 0.0008 | 0.0450 | Downregulated |
| hsa-miR-138-2-3p | 5.85 | 4.80 | 0.0018 | 0.0450 | Downregulated |
| hsa-miR-146b-5p | 1.11 | 0.73 | 0.0008 | 0.0450 | Downregulated |
| hsa-miR-3617-5p | 3.28 | 2.63 | 0.0012 | 0.0450 | Downregulated |
| hsa-miR-3660 | 3.56 | 2.73 | 0.0003 | 0.0450 | Downregulated |
| hsa-miR-425-3p | 13.76 | 16.55 | 0.0005 | 0.0450 | Upregulated |
| hsa-miR-4433-5p | 18.04 | 20.40 | 0.0010 | 0.0450 | Upregulated |
| hsa-miR-4436a | 4.67 | 3.67 | 0.0005 | 0.0450 | Downregulated |
| hsa-miR-4436b-3p | 6.46 | 5.40 | 0.0011 | 0.0450 | Downregulated |
| hsa-miR-4448 | 8.25 | 7.07 | 0.0017 | 0.0450 | Downregulated |
| hsa-miR-4508 | 51.88 | 57.14 | 0.0012 | 0.0450 | Upregulated |
| hsa-miR-452-5p | 8.70 | 7.25 | 0.0009 | 0.0450 | Downregulated |
| hsa-miR-4657 | 1.60 | 1.16 | 0.0018 | 0.0450 | Downregulated |
| hsa-miR-4665-3p | 104.81 | 119.79 | 0.0012 | 0.0450 | Upregulated |
| hsa-miR-4682 | 0.95 | 0.62 | 0.0018 | 0.0450 | Downregulated |
| hsa-miR-4746-5p | 2.91 | 2.33 | 0.0013 | 0.0450 | Downregulated |
| hsa-miR-4748 | 4.64 | 3.78 | 0.0006 | 0.0450 | Downregulated |
| hsa-miR-4787-3p | 94.67 | 106.09 | 0.0003 | 0.0450 | Upregulated |
| hsa-miR-500a-3p | 2.43 | 1.95 | 0.0007 | 0.0450 | Downregulated |
| hsa-miR-509-5p | 7.57 | 6.81 | 0.0018 | 0.0450 | Downregulated |
| hsa-miR-516b-5p | 5.41 | 4.24 | <.0001 | 0.0450 | Downregulated |
| hsa-miR-518a-5p | 4.58 | 3.86 | 0.0013 | 0.0450 | Downregulated |
| hsa-miR-518c-5p | 1.22 | 0.82 | 0.0014 | 0.0450 | Downregulated |
| hsa-miR-519e-5p | 1.93 | 1.32 | 0.0006 | 0.0450 | Downregulated |
| hsa-miR-583 | 3.31 | 2.54 | 0.0018 | 0.0450 | Downregulated |
| hsa-miR-629-3p | 14.57 | 16.06 | 0.0015 | 0.0450 | Upregulated |
| hsa-miR-659-5p | 1.58 | 0.98 | 0.0008 | 0.0450 | Downregulated |
| hsa-miR-766-3p | 26.60 | 29.75 | 0.0017 | 0.0450 | Upregulated |
| hsa-miR-92a-1-5p | 0.50 | 0.33 | 0.0015 | 0.0450 | Downregulated |
| hsa-miR-1225-3p | 18.37 | 20.77 | 0.0019 | 0.0461 | Upregulated |
| hsa-miR-1321 | 4.44 | 3.78 | 0.0021 | 0.0471 | Downregulated |
| hsa-miR-183-3p | 0.56 | 0.34 | 0.0021 | 0.0471 | Downregulated |
| hsa-miR-4296 | 0.72 | 0.46 | 0.0024 | 0.0471 | Downregulated |
| hsa-miR-4510 | 5.28 | 4.62 | 0.0024 | 0.0471 | Downregulated |
| hsa-miR-4749-3p | 13.94 | 15.68 | 0.0022 | 0.0471 | Upregulated |
| hsa-miR-4768-3p | 3.12 | 2.48 | 0.0024 | 0.0471 | Downregulated |
| hsa-miR-4769-5p | 6.80 | 5.90 | 0.0023 | 0.0471 | Downregulated |
| hsa-miR-6069 | 17.67 | 19.96 | 0.0022 | 0.0471 | Upregulated |
| hsa-miR-520b | 16.73 | 18.97 | 0.0025 | 0.0480 | Upregulated |
| hsa-miR-4421 | 1.28 | 0.84 | 0.0027 | 0.0506 | Downregulated |
| hsa-miR-4485 | 1263.45 | 1395.70 | 0.0028 | 0.0513 | Upregulated |
| hsa-miR-339-3p | 3.22 | 2.69 | 0.0029 | 0.0520 | Downregulated |
| hsa-miR-4303 | 1.65 | 1.16 | 0.0030 | 0.0527 | Downregulated |
| hsa-miR-1281 | 48.12 | 55.83 | 0.0031 | 0.0533 | Upregulated |
| hsa-miR-4458 | 5.48 | 4.74 | 0.0033 | 0.0556 | Downregulated |
| hsa-miR-1234-3p | 50.92 | 56.80 | 0.0036 | 0.0560 | Upregulated |
| hsa-miR-3121-3p | 5.61 | 4.71 | 0.0035 | 0.0560 | Downregulated |
| hsa-miR-4436b-5p | 23.81 | 27.76 | 0.0036 | 0.0560 | Upregulated |
| hsa-miR-4638-5p | 2.98 | 2.17 | 0.0036 | 0.0560 | Downregulated |
| hsa-miR-1266 | 1.07 | 0.71 | 0.0040 | 0.0611 | Downregulated |
| hsa-miR-4755-3p | 6.99 | 5.97 | 0.0043 | 0.0645 | Downregulated |
| hsa-miR-200a-5p | 4.60 | 3.91 | 0.0051 | 0.0690 | Downregulated |
| hsa-miR-2117 | 2.21 | 1.67 | 0.0049 | 0.0690 | Downregulated |
| hsa-miR-3122 | 1.33 | 0.94 | 0.0047 | 0.0690 | Downregulated |
| hsa-miR-4654 | 1.66 | 1.17 | 0.0051 | 0.0690 | Downregulated |
| hsa-miR-4767 | 26.19 | 29.05 | 0.0051 | 0.0690 | Upregulated |
| hsa-miR-4773 | 7.99 | 6.93 | 0.0048 | 0.0690 | Downregulated |
| hsa-miR-658 | 0.64 | 0.48 | 0.0053 | 0.0705 | Downregulated |
| hsa-miR-1228-3p | 31.57 | 34.98 | 0.0056 | 0.0722 | Upregulated |
| hsa-miR-4725-5p | 11.48 | 13.21 | 0.0056 | 0.0722 | Upregulated |
| hsa-miR-1303 | 2.74 | 2.17 | 0.0068 | 0.0759 | Downregulated |
| hsa-miR-206 | 9.23 | 8.35 | 0.0062 | 0.0759 | Downregulated |
| hsa-miR-28-5p | 1.21 | 0.83 | 0.0064 | 0.0759 | Downregulated |
| hsa-miR-3648 | 253.35 | 275.72 | 0.0064 | 0.0759 | Upregulated |
| hsa-miR-3935 | 16.21 | 17.66 | 0.0068 | 0.0759 | Upregulated |
| hsa-miR-4418 | 5.10 | 4.41 | 0.0069 | 0.0759 | Downregulated |
| hsa-miR-4709-3p | 0.80 | 0.57 | 0.0069 | 0.0759 | Downregulated |
| hsa-miR-4722-5p | 2.99 | 2.30 | 0.0069 | 0.0759 | Downregulated |
| hsa-miR-4731-3p | 1.02 | 0.73 | 0.0063 | 0.0759 | Downregulated |
| hsa-miR-5572 | 5.96 | 5.15 | 0.0068 | 0.0759 | Downregulated |
| hsa-miR-5696 | 5.82 | 4.81 | 0.0067 | 0.0759 | Downregulated |
| hsa-miR-1291 | 3.04 | 2.55 | 0.0073 | 0.0792 | Downregulated |
| hsa-miR-4728-3p | 13.04 | 14.97 | 0.0075 | 0.0793 | Upregulated |
| hsa-miR-940 | 736.26 | 826.12 | 0.0074 | 0.0793 | Upregulated |
| hsa-miR-4499 | 405.42 | 441.10 | 0.0078 | 0.0804 | Upregulated |
| hsa-miR-671-3p | 0.67 | 0.47 | 0.0077 | 0.0804 | Downregulated |
| hsa-miR-139-5p | 3.07 | 2.54 | 0.0083 | 0.0815 | Downregulated |
| hsa-miR-3162-3p | 15.58 | 18.84 | 0.0083 | 0.0815 | Upregulated |
| hsa-miR-3186-3p | 1.00 | 0.66 | 0.0083 | 0.0815 | Downregulated |
| hsa-miR-4280 | 2.67 | 2.01 | 0.0084 | 0.0815 | Downregulated |
| hsa-miR-5093 | 3.33 | 2.83 | 0.0085 | 0.0815 | Downregulated |
| hsa-miR-526b-5p | 3.41 | 3.01 | 0.0085 | 0.0815 | Downregulated |
| hsa-miR-23a-5p | 6.17 | 5.52 | 0.0093 | 0.0825 | Downregulated |
| hsa-miR-3680-3p | 6.15 | 5.62 | 0.0094 | 0.0825 | Downregulated |
| hsa-miR-4313 | 26.84 | 29.17 | 0.0094 | 0.0825 | Upregulated |
| hsa-miR-4316 | 4.28 | 3.50 | 0.0089 | 0.0825 | Downregulated |
| hsa-miR-432-5p | 1.67 | 1.27 | 0.0089 | 0.0825 | Downregulated |
| hsa-miR-4506 | 5.97 | 5.27 | 0.0094 | 0.0825 | Downregulated |
| hsa-miR-4726-5p | 18.86 | 19.73 | 0.0094 | 0.0825 | Upregulated |
| hsa-miR-493-3p | 2.25 | 1.85 | 0.0088 | 0.0825 | Downregulated |
| hsa-miR-3163 | 7.58 | 6.78 | 0.0097 | 0.0842 | Downregulated |
| hsa-miR-146a-5p | 4.92 | 4.29 | 0.0100 | 0.0859 | Downregulated |
| hsa-miR-197-3p | 15.12 | 17.75 | 0.0103 | 0.0867 | Upregulated |
| hsa-miR-4676-5p | 1.44 | 1.07 | 0.0103 | 0.0867 | Downregulated |
| hsa-miR-192-3p | 1.98 | 1.47 | 0.0105 | 0.0875 | Downregulated |
| hsa-miR-6087 | 14790.16 | 16064.57 | 0.0107 | 0.0883 | Upregulated |
| ***TGFBR1*** (rs6478974) | TT ( N=150 ) | TA/AA ( N=298 ) |  |  |  |
| hsa-miR-3917 | 94.31 | 87.13 | <.0001 | 0.0825 | Downregulated |

| Supplemental Table 4. SNPs in TGFBeta-signaling pathway associated with miRNAs in differential colon tissue; FDR<0.09 | | | | | |
| --- | --- | --- | --- | --- | --- |
| miRNA | Mean | Mean | P-values | FDR adjusted P | Direction |
| ***BMPR1B*** (rs12508087) | TT ( N=378 ) | TA/AA ( N=252 ) |  |  |  |
| hsa-miR-1182 | 0.29 | 3.37 | 0.0008 | 0.0165 | Upregulated |
| hsa-miR-1224-5p | -127.48 | -16.55 | 0.0008 | 0.0165 | Upregulated |
| hsa-miR-1225-5p | -422.11 | 83.75 | 0.0009 | 0.0165 | Upregulated |
| hsa-miR-1228-3p | -2.66 | 2.94 | 0.0006 | 0.0165 | Upregulated |
| hsa-miR-1233-1-5p | -26.41 | -7.97 | 0.0012 | 0.0165 | Upregulated |
| hsa-miR-1234-5p | -828.58 | -28.93 | 0.0012 | 0.0165 | Upregulated |
| hsa-miR-1268b | -24.76 | 179.09 | 0.0003 | 0.0165 | Upregulated |
| hsa-miR-138-2-3p | -0.75 | -1.86 | 0.0002 | 0.0165 | Downregulated |
| hsa-miR-140-3p | -1.45 | -2.78 | <.0001 | 0.0165 | Downregulated |
| hsa-miR-146b-5p | 1.07 | 0.36 | 0.0004 | 0.0165 | Downregulated |
| hsa-miR-150-3p | -31.45 | 11.22 | 0.0007 | 0.0165 | Upregulated |
| hsa-miR-188-5p | -76.36 | 2.88 | 0.0010 | 0.0165 | Upregulated |
| hsa-miR-1915-3p | -668.47 | -162.98 | 0.0003 | 0.0165 | Upregulated |
| hsa-miR-194-3p | -1.66 | -0.88 | 0.0006 | 0.0165 | Upregulated |
| hsa-miR-2861 | -1203.17 | -328.03 | 0.0002 | 0.0165 | Upregulated |
| hsa-miR-3141 | 5.19 | 45.05 | 0.0011 | 0.0165 | Upregulated |
| hsa-miR-345-3p | -1.80 | 7.61 | 0.0008 | 0.0165 | Upregulated |
| hsa-miR-3621 | -3.53 | 0.83 | 0.0011 | 0.0165 | Upregulated |
| hsa-miR-3648 | 38.94 | 92.29 | <.0001 | 0.0165 | Upregulated |
| hsa-miR-3656 | -298.14 | 144.78 | 0.0004 | 0.0165 | Upregulated |
| hsa-miR-3665 | -590.36 | -110.88 | 0.0004 | 0.0165 | Upregulated |
| hsa-miR-3940-5p | -127.51 | -9.57 | 0.0008 | 0.0165 | Upregulated |
| hsa-miR-4298 | 12.36 | 42.06 | 0.0009 | 0.0165 | Upregulated |
| hsa-miR-4322 | 3.79 | 16.68 | 0.0003 | 0.0165 | Upregulated |
| hsa-miR-4433-3p | -32.27 | 34.14 | 0.0008 | 0.0165 | Upregulated |
| hsa-miR-4436a | -0.07 | -0.82 | 0.0010 | 0.0165 | Downregulated |
| hsa-miR-4463 | -20.48 | 28.51 | 0.0005 | 0.0165 | Upregulated |
| hsa-miR-4466 | -264.72 | 32.20 | 0.0012 | 0.0165 | Upregulated |
| hsa-miR-4507 | -330.49 | -70.42 | 0.0006 | 0.0165 | Upregulated |
| hsa-miR-4508 | -10.99 | -5.08 | 0.0011 | 0.0165 | Upregulated |
| hsa-miR-4640-5p | 2.32 | 5.29 | 0.0005 | 0.0165 | Upregulated |
| hsa-miR-4687-3p | -435.67 | -17.28 | 0.0006 | 0.0165 | Upregulated |
| hsa-miR-4688 | 1.16 | 5.27 | 0.0004 | 0.0165 | Upregulated |
| hsa-miR-4690-5p | -54.41 | -29.56 | 0.0010 | 0.0165 | Upregulated |
| hsa-miR-4701-3p | -0.09 | 7.90 | 0.0011 | 0.0165 | Upregulated |
| hsa-miR-4707-5p | -7.99 | 7.31 | 0.0006 | 0.0165 | Upregulated |
| hsa-miR-4710 | 0.85 | 5.10 | 0.0011 | 0.0165 | Upregulated |
| hsa-miR-4741 | -91.90 | 96.96 | 0.0009 | 0.0165 | Upregulated |
| hsa-miR-483-5p | 3.36 | 25.50 | 0.0010 | 0.0165 | Upregulated |
| hsa-miR-487b | 0.04 | -0.44 | 0.0011 | 0.0165 | Downregulated |
| hsa-miR-498 | -3.77 | 0.20 | 0.0008 | 0.0165 | Upregulated |
| hsa-miR-5196-5p | 6.80 | 16.80 | 0.0006 | 0.0165 | Upregulated |
| hsa-miR-5585-3p | -7.93 | 25.37 | 0.0011 | 0.0165 | Upregulated |
| hsa-miR-572 | -95.60 | -27.10 | 0.0007 | 0.0165 | Upregulated |
| hsa-miR-6068 | -507.73 | -158.29 | 0.0005 | 0.0165 | Upregulated |
| hsa-miR-6069 | -1.29 | 2.00 | 0.0003 | 0.0165 | Upregulated |
| hsa-miR-6076 | -32.27 | -1.35 | 0.0006 | 0.0165 | Upregulated |
| hsa-miR-6083 | -8.29 | -4.69 | 0.0006 | 0.0165 | Upregulated |
| hsa-miR-6088 | -412.01 | 127.38 | 0.0012 | 0.0165 | Upregulated |
| hsa-miR-6089 | -5587.19 | -1383.72 | 0.0010 | 0.0165 | Upregulated |
| hsa-miR-6165 | 17.88 | 87.26 | 0.0008 | 0.0165 | Upregulated |
| hsa-miR-623 | -5.24 | 0.67 | 0.0010 | 0.0165 | Upregulated |
| hsa-miR-638 | -690.46 | -126.32 | 0.0008 | 0.0165 | Upregulated |
| hsa-miR-6511a-5p | 1.31 | 6.67 | 0.0003 | 0.0165 | Upregulated |
| hsa-miR-671-5p | -49.00 | 2.17 | 0.0003 | 0.0165 | Upregulated |
| hsa-miR-6722-3p | -11.44 | 7.03 | <.0001 | 0.0165 | Upregulated |
| hsa-miR-6724-5p | -90.62 | 32.59 | 0.0012 | 0.0165 | Upregulated |
| hsa-miR-718 | -17.75 | 1.48 | 0.0004 | 0.0165 | Upregulated |
| hsa-miR-937-5p | -43.41 | 42.52 | 0.0009 | 0.0165 | Upregulated |
| hsa-miR-3937 | -6.32 | 3.66 | 0.0013 | 0.0170 | Upregulated |
| hsa-miR-4734 | -24.78 | -2.09 | 0.0013 | 0.0170 | Upregulated |
| hsa-miR-6073 | -1.63 | -2.34 | 0.0013 | 0.0170 | Downregulated |
| hsa-miR-130a-3p | 1.36 | 0.42 | 0.0015 | 0.0172 | Downregulated |
| hsa-miR-1471 | -33.02 | 4.05 | 0.0015 | 0.0172 | Upregulated |
| hsa-miR-3620-5p | -15.30 | -2.07 | 0.0015 | 0.0172 | Upregulated |
| hsa-miR-3679-5p | -114.53 | 0.22 | 0.0015 | 0.0172 | Upregulated |
| hsa-miR-4486 | -15.90 | 3.35 | 0.0015 | 0.0172 | Upregulated |
| hsa-miR-4655-5p | -1.51 | 2.81 | 0.0014 | 0.0172 | Upregulated |
| hsa-miR-4726-5p | -1.26 | 0.58 | 0.0015 | 0.0172 | Upregulated |
| hsa-miR-4743-5p | -1.17 | 3.66 | 0.0015 | 0.0172 | Upregulated |
| hsa-miR-6510-5p | -14.03 | 51.66 | 0.0014 | 0.0172 | Upregulated |
| hsa-miR-1207-5p | -281.57 | 48.63 | 0.0017 | 0.0177 | Upregulated |
| hsa-miR-1227-5p | -171.83 | -27.93 | 0.0017 | 0.0177 | Upregulated |
| hsa-miR-1236-5p | -6.75 | 14.22 | 0.0016 | 0.0177 | Upregulated |
| hsa-miR-1323 | -0.30 | -0.72 | 0.0017 | 0.0177 | Downregulated |
| hsa-miR-4689 | 0.13 | 22.15 | 0.0017 | 0.0177 | Upregulated |
| hsa-miR-6087 | -2917.17 | -895.59 | 0.0017 | 0.0177 | Upregulated |
| hsa-miR-6125 | -2244.91 | -885.70 | 0.0017 | 0.0177 | Upregulated |
| hsa-miR-1226-5p | -4.36 | 2.82 | 0.0020 | 0.0179 | Upregulated |
| hsa-miR-1268a | 178.74 | 349.58 | 0.0022 | 0.0179 | Upregulated |
| hsa-miR-134 | -37.61 | 4.55 | 0.0022 | 0.0179 | Upregulated |
| hsa-miR-135a-3p | -22.81 | -0.29 | 0.0022 | 0.0179 | Upregulated |
| hsa-miR-1587 | -113.15 | 36.92 | 0.0021 | 0.0179 | Upregulated |
| hsa-miR-3147 | 0.67 | 4.32 | 0.0021 | 0.0179 | Upregulated |
| hsa-miR-3162-5p | -394.10 | 30.63 | 0.0018 | 0.0179 | Upregulated |
| hsa-miR-3185 | -0.52 | 2.05 | 0.0019 | 0.0179 | Upregulated |
| hsa-miR-33b-3p | -2.18 | -0.22 | 0.0021 | 0.0179 | Upregulated |
| hsa-miR-3663-3p | -24.44 | 2.79 | 0.0021 | 0.0179 | Upregulated |
| hsa-miR-4433-5p | -1.49 | 2.21 | 0.0019 | 0.0179 | Upregulated |
| hsa-miR-4488 | -7.89 | 4.88 | 0.0021 | 0.0179 | Upregulated |
| hsa-miR-4496 | -6.79 | 1.30 | 0.0021 | 0.0179 | Upregulated |
| hsa-miR-4516 | -3152.98 | -766.19 | 0.0022 | 0.0179 | Upregulated |
| hsa-miR-4532 | -65.64 | -27.31 | 0.0022 | 0.0179 | Upregulated |
| hsa-miR-4669 | -26.18 | 16.73 | 0.0018 | 0.0179 | Upregulated |
| hsa-miR-4672 | -75.77 | 4.65 | 0.0019 | 0.0179 | Upregulated |
| hsa-miR-4745-5p | -39.84 | -6.55 | 0.0020 | 0.0179 | Upregulated |
| hsa-miR-557 | -3.34 | 7.74 | 0.0019 | 0.0179 | Upregulated |
| hsa-miR-6075 | -47.23 | -20.63 | 0.0022 | 0.0179 | Upregulated |
| hsa-miR-939-5p | -79.61 | 9.45 | 0.0018 | 0.0179 | Upregulated |
| hsa-miR-99b-5p | 1.34 | 0.66 | 0.0021 | 0.0179 | Downregulated |
| hsa-miR-1183 | -2.63 | 2.65 | 0.0024 | 0.0182 | Upregulated |
| hsa-miR-3911 | 7.01 | 18.38 | 0.0023 | 0.0182 | Upregulated |
| hsa-miR-4271 | -23.82 | 16.91 | 0.0024 | 0.0182 | Upregulated |
| hsa-miR-4327 | -7.75 | 32.89 | 0.0024 | 0.0182 | Upregulated |
| hsa-miR-5001-5p | -228.37 | -85.34 | 0.0023 | 0.0182 | Upregulated |
| hsa-miR-5088 | -10.55 | -4.23 | 0.0024 | 0.0182 | Upregulated |
| hsa-miR-5787 | -416.75 | 223.84 | 0.0024 | 0.0182 | Upregulated |
| hsa-miR-1202 | -170.97 | 117.20 | 0.0027 | 0.0183 | Upregulated |
| hsa-miR-1273f | -16.02 | 7.40 | 0.0027 | 0.0183 | Upregulated |
| hsa-miR-365a-5p | -1.22 | 1.22 | 0.0027 | 0.0183 | Upregulated |
| hsa-miR-3917 | -3.53 | 4.99 | 0.0027 | 0.0183 | Upregulated |
| hsa-miR-4314 | -4.65 | 0.97 | 0.0027 | 0.0183 | Upregulated |
| hsa-miR-4505 | -411.82 | -59.53 | 0.0026 | 0.0183 | Upregulated |
| hsa-miR-4539 | -32.02 | -25.94 | 0.0026 | 0.0183 | Upregulated |
| hsa-miR-4695-5p | -40.03 | -7.71 | 0.0026 | 0.0183 | Upregulated |
| hsa-miR-4738-3p | -1.42 | 3.12 | 0.0027 | 0.0183 | Upregulated |
| hsa-miR-4778-5p | 10.72 | 25.41 | 0.0025 | 0.0183 | Upregulated |
| hsa-miR-602 | -4.10 | 0.38 | 0.0025 | 0.0183 | Upregulated |
| hsa-miR-6511b-5p | 0.22 | 7.10 | 0.0027 | 0.0183 | Upregulated |
| hsa-miR-762 | -294.97 | -81.37 | 0.0025 | 0.0183 | Upregulated |
| hsa-miR-1185-1-3p | -50.26 | 15.50 | 0.0030 | 0.0186 | Upregulated |
| hsa-miR-3188 | -41.77 | -5.50 | 0.0030 | 0.0186 | Upregulated |
| hsa-miR-3960 | -1578.98 | 148.02 | 0.0029 | 0.0186 | Upregulated |
| hsa-miR-4417 | 18.41 | 47.42 | 0.0029 | 0.0186 | Upregulated |
| hsa-miR-4419a | -0.12 | 9.45 | 0.0030 | 0.0186 | Upregulated |
| hsa-miR-4497 | -386.07 | -33.23 | 0.0029 | 0.0186 | Upregulated |
| hsa-miR-4530 | -656.63 | 83.93 | 0.0030 | 0.0186 | Upregulated |
| hsa-miR-4534 | -45.03 | -8.34 | 0.0030 | 0.0186 | Upregulated |
| hsa-miR-4634 | -68.14 | -20.16 | 0.0029 | 0.0186 | Upregulated |
| hsa-miR-4749-5p | -0.75 | 0.91 | 0.0028 | 0.0186 | Upregulated |
| hsa-miR-6090 | -1010.73 | 45.17 | 0.0029 | 0.0186 | Upregulated |
| hsa-miR-3202 | -2.14 | 1.33 | 0.0031 | 0.0188 | Upregulated |
| hsa-miR-4758-5p | -3.00 | 22.38 | 0.0031 | 0.0188 | Upregulated |
| hsa-miR-642b-3p | -82.47 | 33.39 | 0.0031 | 0.0188 | Upregulated |
| hsa-miR-1185-2-3p | -13.97 | 13.78 | 0.0032 | 0.0190 | Upregulated |
| hsa-miR-1275 | -123.14 | 2.95 | 0.0032 | 0.0190 | Upregulated |
| hsa-miR-3138 | -5.84 | 5.35 | 0.0032 | 0.0190 | Upregulated |
| hsa-miR-4257 | -42.61 | -3.36 | 0.0033 | 0.0194 | Upregulated |
| hsa-miR-1914-3p | -14.62 | -0.34 | 0.0034 | 0.0199 | Upregulated |
| hsa-miR-4763-3p | -138.51 | 88.71 | 0.0035 | 0.0203 | Upregulated |
| hsa-miR-1229-5p | -73.52 | 63.68 | 0.0036 | 0.0206 | Upregulated |
| hsa-miR-4800-5p | 17.94 | 47.18 | 0.0036 | 0.0206 | Upregulated |
| hsa-miR-3194-5p | -20.75 | 2.27 | 0.0037 | 0.0207 | Upregulated |
| hsa-miR-4721 | -201.31 | -57.63 | 0.0037 | 0.0207 | Upregulated |
| hsa-miR-5190 | -1.60 | 0.73 | 0.0037 | 0.0207 | Upregulated |
| hsa-miR-3130-5p | -0.16 | -0.48 | 0.0039 | 0.0216 | Downregulated |
| hsa-miR-3196 | -198.72 | -68.82 | 0.0039 | 0.0216 | Upregulated |
| hsa-miR-4646-5p | -6.41 | 5.32 | 0.0040 | 0.0218 | Upregulated |
| hsa-miR-601 | -6.72 | -0.34 | 0.0040 | 0.0218 | Upregulated |
| hsa-miR-1261 | -0.63 | -1.16 | 0.0042 | 0.0226 | Downregulated |
| hsa-miR-497-5p | -3.27 | -3.84 | 0.0042 | 0.0226 | Downregulated |
| hsa-miR-5696 | -0.82 | -1.77 | 0.0045 | 0.0241 | Downregulated |
| hsa-miR-142-3p | -1.84 | -2.31 | 0.0046 | 0.0242 | Downregulated |
| hsa-miR-15a-5p | 1.99 | 1.12 | 0.0047 | 0.0242 | Downregulated |
| hsa-miR-3652 | -12.83 | -1.34 | 0.0047 | 0.0242 | Upregulated |
| hsa-miR-4281 | -369.45 | 326.97 | 0.0048 | 0.0242 | Upregulated |
| hsa-miR-4459 | -5356.84 | -940.79 | 0.0047 | 0.0242 | Upregulated |
| hsa-miR-4481 | -9.68 | 0.59 | 0.0048 | 0.0242 | Upregulated |
| hsa-miR-4484 | -10.34 | -2.40 | 0.0048 | 0.0242 | Upregulated |
| hsa-miR-5006-5p | -105.22 | -41.77 | 0.0047 | 0.0242 | Upregulated |
| hsa-miR-6085 | -82.79 | 27.55 | 0.0046 | 0.0242 | Upregulated |
| hsa-miR-4442 | -17.92 | 23.16 | 0.0049 | 0.0244 | Upregulated |
| hsa-miR-514b-5p | 0.10 | 4.32 | 0.0049 | 0.0244 | Upregulated |
| hsa-miR-3195 | -219.51 | -80.21 | 0.0052 | 0.0258 | Upregulated |
| hsa-miR-3610 | -26.45 | -3.77 | 0.0054 | 0.0264 | Upregulated |
| hsa-miR-663a | 134.69 | 170.11 | 0.0054 | 0.0264 | Upregulated |
| hsa-miR-1249 | -2.18 | 4.01 | 0.0055 | 0.0265 | Upregulated |
| hsa-miR-3934-5p | -2.00 | 0.56 | 0.0056 | 0.0265 | Upregulated |
| hsa-miR-4499 | -70.13 | -22.08 | 0.0055 | 0.0265 | Upregulated |
| hsa-miR-4632-5p | -17.83 | 2.78 | 0.0056 | 0.0265 | Upregulated |
| hsa-miR-6126 | -119.02 | -43.70 | 0.0056 | 0.0265 | Upregulated |
| hsa-miR-6127 | -167.51 | -9.59 | 0.0055 | 0.0265 | Upregulated |
| hsa-miR-4787-5p | -348.69 | -197.23 | 0.0057 | 0.0268 | Upregulated |
| hsa-miR-4253 | -3.93 | 0.34 | 0.0058 | 0.0271 | Upregulated |
| hsa-miR-1225-3p | -2.32 | -0.12 | 0.0059 | 0.0274 | Upregulated |
| hsa-miR-6723-5p | -11.63 | 1.52 | 0.0060 | 0.0277 | Upregulated |
| hsa-miR-642a-3p | -518.64 | 126.90 | 0.0061 | 0.0280 | Upregulated |
| hsa-miR-4270 | -35.50 | 77.33 | 0.0062 | 0.0282 | Upregulated |
| hsa-miR-550a-3-5p | -0.88 | 1.66 | 0.0062 | 0.0282 | Upregulated |
| hsa-miR-199b-5p | 1.26 | 0.91 | 0.0065 | 0.0294 | Downregulated |
| hsa-miR-1273e | -9.89 | -0.54 | 0.0066 | 0.0295 | Upregulated |
| hsa-miR-5008-5p | -2.42 | 1.92 | 0.0066 | 0.0295 | Upregulated |
| hsa-miR-214-3p | 6.03 | 5.60 | 0.0068 | 0.0296 | Downregulated |
| hsa-miR-4465 | -105.78 | 61.17 | 0.0068 | 0.0296 | Upregulated |
| hsa-miR-4673 | -17.47 | -9.33 | 0.0067 | 0.0296 | Upregulated |
| hsa-miR-760 | -1.38 | 2.28 | 0.0067 | 0.0296 | Upregulated |
| hsa-miR-765 | 0.27 | 5.26 | 0.0068 | 0.0296 | Upregulated |
| hsa-miR-4651 | -22.42 | 11.48 | 0.0069 | 0.0298 | Upregulated |
| hsa-miR-3174 | 0.43 | -0.25 | 0.0070 | 0.0301 | Downregulated |
| hsa-miR-3692-5p | -6.39 | 2.89 | 0.0071 | 0.0304 | Upregulated |
| hsa-miR-4429 | -0.98 | 2.48 | 0.0072 | 0.0306 | Upregulated |
| hsa-miR-489 | -0.21 | -1.07 | 0.0073 | 0.0309 | Downregulated |
| hsa-miR-143-3p | -0.48 | -1.65 | 0.0075 | 0.0316 | Downregulated |
| hsa-miR-4746-3p | -47.81 | -13.15 | 0.0076 | 0.0317 | Upregulated |
| hsa-miR-769-3p | -3.99 | -1.99 | 0.0076 | 0.0317 | Upregulated |
| hsa-miR-99b-3p | -6.17 | -1.09 | 0.0078 | 0.0324 | Upregulated |
| hsa-miR-622 | -2.93 | 9.12 | 0.0079 | 0.0326 | Upregulated |
| hsa-miR-2392 | -10.33 | 26.44 | 0.0080 | 0.0327 | Upregulated |
| hsa-miR-3660 | -0.58 | -1.15 | 0.0080 | 0.0327 | Downregulated |
| hsa-miR-429 | 4.01 | 3.87 | 0.0081 | 0.0329 | Upregulated |
| hsa-miR-512-3p | -0.92 | -1.15 | 0.0082 | 0.0332 | Downregulated |
| hsa-miR-3654 | 1.99 | 4.07 | 0.0084 | 0.0336 | Upregulated |
| hsa-miR-4317 | 0.64 | 0.33 | 0.0084 | 0.0336 | Downregulated |
| hsa-miR-617 | -8.49 | -3.81 | 0.0085 | 0.0339 | Upregulated |
| hsa-miR-4443 | -257.01 | -36.22 | 0.0086 | 0.0341 | Upregulated |
| hsa-miR-1181 | -25.94 | 0.92 | 0.0089 | 0.0351 | Upregulated |
| hsa-miR-3162-3p | -1.05 | 1.22 | 0.0090 | 0.0353 | Upregulated |
| hsa-miR-4739 | -148.02 | -36.69 | 0.0092 | 0.0358 | Upregulated |
| hsa-miR-4787-3p | -20.00 | -7.48 | 0.0092 | 0.0358 | Upregulated |
| hsa-miR-3158-5p | -3.55 | 0.28 | 0.0094 | 0.0364 | Upregulated |
| hsa-miR-149-3p | -3.49 | 0.26 | 0.0095 | 0.0366 | Upregulated |
| hsa-miR-125a-3p | -30.53 | -8.73 | 0.0096 | 0.0368 | Upregulated |
| hsa-miR-198 | -5.46 | 0.24 | 0.0100 | 0.0382 | Upregulated |
| hsa-miR-4636 | -0.13 | -0.55 | 0.0102 | 0.0387 | Downregulated |
| hsa-miR-5195-3p | -17.25 | 8.72 | 0.0106 | 0.0401 | Upregulated |
| hsa-miR-2276 | 1.46 | 16.29 | 0.0108 | 0.0406 | Upregulated |
| hsa-miR-4767 | -3.12 | 0.23 | 0.0110 | 0.0412 | Upregulated |
| hsa-miR-6086 | -28.35 | 28.66 | 0.0112 | 0.0418 | Upregulated |
| hsa-miR-484 | 0.06 | -0.25 | 0.0113 | 0.0419 | Downregulated |
| hsa-miR-610 | -1.11 | 1.00 | 0.0115 | 0.0425 | Upregulated |
| hsa-miR-200a-5p | 1.28 | 0.59 | 0.0116 | 0.0427 | Downregulated |
| hsa-miR-4793-5p | 1.34 | 21.87 | 0.0118 | 0.0432 | Upregulated |
| hsa-miR-2467-3p | 1.23 | 2.31 | 0.0119 | 0.0434 | Upregulated |
| hsa-miR-4788 | -31.31 | 23.86 | 0.0121 | 0.0437 | Upregulated |
| hsa-miR-874 | -24.91 | -4.63 | 0.0121 | 0.0437 | Upregulated |
| hsa-miR-4535 | -6.14 | -0.34 | 0.0122 | 0.0439 | Upregulated |
| hsa-miR-6124 | -22.10 | 86.10 | 0.0123 | 0.0440 | Upregulated |
| hsa-miR-4776-5p | -1.53 | 0.62 | 0.0126 | 0.0447 | Upregulated |
| hsa-miR-921 | -4.00 | -4.74 | 0.0126 | 0.0447 | Downregulated |
| hsa-miR-5189 | -1.63 | 0.76 | 0.0127 | 0.0449 | Upregulated |
| hsa-miR-373-5p | -1.41 | 0.69 | 0.0130 | 0.0457 | Upregulated |
| hsa-miR-30c-5p | -2.32 | -2.70 | 0.0132 | 0.0459 | Downregulated |
| hsa-miR-30e-5p | -0.84 | -1.34 | 0.0132 | 0.0459 | Downregulated |
| hsa-miR-4450 | 0.44 | -0.21 | 0.0131 | 0.0459 | Downregulated |
| hsa-miR-1247-3p | 0.46 | 3.26 | 0.0133 | 0.0460 | Upregulated |
| hsa-miR-500a-3p | 0.10 | -0.30 | 0.0135 | 0.0465 | Downregulated |
| hsa-miR-1229-3p | -5.43 | 3.11 | 0.0136 | 0.0466 | Upregulated |
| hsa-miR-99a-5p | 1.35 | 0.85 | 0.0140 | 0.0478 | Downregulated |
| hsa-miR-125b-5p | 12.69 | 9.50 | 0.0143 | 0.0484 | Downregulated |
| hsa-miR-30a-5p | -1.04 | -1.66 | 0.0143 | 0.0484 | Downregulated |
| hsa-miR-320c | -20.18 | 32.39 | 0.0146 | 0.0492 | Upregulated |
| hsa-miR-3177-5p | -0.91 | -1.28 | 0.0147 | 0.0494 | Downregulated |
| hsa-miR-1307-5p | -18.33 | -12.96 | 0.0152 | 0.0506 | Upregulated |
| hsa-miR-4513 | -1.46 | 1.26 | 0.0152 | 0.0506 | Upregulated |
| hsa-miR-3154 | -1.62 | 0.56 | 0.0154 | 0.0509 | Upregulated |
| hsa-miR-584-5p | -2.91 | -1.17 | 0.0154 | 0.0509 | Upregulated |
| hsa-miR-4446-3p | -2.25 | -3.24 | 0.0156 | 0.0511 | Upregulated |
| hsa-miR-4476 | -6.10 | 3.93 | 0.0156 | 0.0511 | Upregulated |
| hsa-miR-3173-3p | 0.62 | -0.13 | 0.0158 | 0.0516 | Downregulated |
| hsa-miR-3614-5p | -1.05 | -1.21 | 0.0164 | 0.0533 | Upregulated |
| hsa-miR-4419b | -2.02 | 0.34 | 0.0165 | 0.0534 | Upregulated |
| hsa-miR-1306-3p | -0.14 | 2.28 | 0.0170 | 0.0548 | Upregulated |
| hsa-miR-100-5p | 1.77 | 0.39 | 0.0173 | 0.0552 | Downregulated |
| hsa-miR-139-3p | -2.91 | -1.49 | 0.0173 | 0.0552 | Upregulated |
| hsa-miR-195-5p | -6.29 | -7.02 | 0.0172 | 0.0552 | Downregulated |
| hsa-miR-197-5p | -426.19 | -88.47 | 0.0174 | 0.0553 | Upregulated |
| hsa-miR-342-3p | -5.14 | -5.37 | 0.0177 | 0.0560 | Upregulated |
| hsa-miR-371b-5p | -369.43 | -199.28 | 0.0179 | 0.0562 | Upregulated |
| hsa-miR-4783-3p | -3.03 | -1.61 | 0.0179 | 0.0562 | Upregulated |
| hsa-miR-4728-3p | -1.26 | 0.42 | 0.0180 | 0.0563 | Upregulated |
| hsa-miR-628-3p | -1.86 | -2.28 | 0.0184 | 0.0573 | Downregulated |
| hsa-miR-22-3p | 1.83 | 1.06 | 0.0188 | 0.0583 | Upregulated |
| hsa-miR-3127-5p | -4.76 | -2.03 | 0.0199 | 0.0615 | Upregulated |
| hsa-miR-936 | -0.15 | 3.31 | 0.0201 | 0.0619 | Upregulated |
| hsa-let-7i-5p | 18.60 | 17.55 | 0.0205 | 0.0629 | Upregulated |
| hsa-miR-4685-5p | -1.12 | 0.66 | 0.0206 | 0.0630 | Upregulated |
| hsa-miR-3682-3p | -7.98 | -3.21 | 0.0208 | 0.0633 | Upregulated |
| hsa-miR-150-5p | -25.80 | -23.28 | 0.0211 | 0.0640 | Upregulated |
| hsa-miR-146a-5p | 2.60 | 1.21 | 0.0212 | 0.0641 | Downregulated |
| hsa-miR-4296 | 0.48 | 0.20 | 0.0214 | 0.0644 | Downregulated |
| hsa-miR-4746-5p | 0.77 | 0.20 | 0.0217 | 0.0651 | Downregulated |
| hsa-miR-4665-3p | -17.24 | -6.21 | 0.0223 | 0.0666 | Upregulated |
| hsa-miR-3137 | -12.26 | 2.79 | 0.0225 | 0.0668 | Upregulated |
| hsa-miR-4430 | -163.06 | -44.46 | 0.0226 | 0.0668 | Upregulated |
| hsa-miR-566 | -0.27 | -0.61 | 0.0225 | 0.0668 | Downregulated |
| hsa-miR-630 | -94.75 | -3.14 | 0.0228 | 0.0672 | Upregulated |
| hsa-miR-3135b | -39.12 | -2.69 | 0.0236 | 0.0693 | Upregulated |
| hsa-miR-4665-5p | -4.03 | 0.99 | 0.0243 | 0.0711 | Upregulated |
| hsa-miR-5572 | -0.33 | -0.81 | 0.0245 | 0.0714 | Downregulated |
| hsa-miR-877-5p | -0.84 | 2.86 | 0.0260 | 0.0755 | Upregulated |
| hsa-miR-664a-5p | 0.65 | -0.38 | 0.0262 | 0.0758 | Downregulated |
| hsa-miR-4316 | -1.27 | -1.98 | 0.0273 | 0.0787 | Downregulated |
| hsa-miR-4515 | -47.26 | -26.05 | 0.0278 | 0.0796 | Upregulated |
| hsa-miR-4748 | -0.22 | -0.40 | 0.0277 | 0.0796 | Downregulated |
| hsa-miR-509-3-5p | -0.56 | -0.90 | 0.0288 | 0.0821 | Upregulated |
| hsa-miR-4487 | -10.76 | -4.77 | 0.0297 | 0.0838 | Upregulated |
| hsa-miR-4709-3p | 0.25 | 0.03 | 0.0296 | 0.0838 | Downregulated |
| hsa-miR-6074 | -0.46 | 0.69 | 0.0295 | 0.0838 | Upregulated |
| hsa-miR-5739 | -71.03 | 87.71 | 0.0305 | 0.0858 | Upregulated |
| hsa-miR-3121-3p | -0.02 | -0.78 | 0.0308 | 0.0863 | Downregulated |
| hsa-miR-766-3p | -4.33 | -2.27 | 0.0318 | 0.0885 | Upregulated |
| hsa-miR-940 | -141.22 | -87.18 | 0.0318 | 0.0885 | Upregulated |
| hsa-miR-5703 | -60.42 | 3.48 | 0.0320 | 0.0888 | Upregulated |
| ***BMPR1B*** (rs2120834) | GG/GC ( N=545 ) | CC ( N=85 ) |  |  |  |
| hsa-miR-4638-3p | -0.02 | 0.66 | <.0001 | 0.0813 | Upregulated |
| ***BMPR2*** (rs2228545) | GG ( N=598 ) | GA/AA ( N=32 ) |  |  |  |
| hsa-miR-3676-3p | 0.06 | 3.22 | <.0001 | 0.0406 | Upregulated |
| hsa-miR-550a-5p | -0.86 | 1.65 | <.0001 | 0.0406 | Upregulated |
| hsa-miR-933 | -0.30 | 1.13 | 0.0003 | 0.0813 | Upregulated |
| ***EIF4EBP3*** (rs250425) | CC ( N=398 ) | CT/TT ( N=235 ) |  |  |  |
| hsa-let-7g-5p | 9.14 | -2.75 | 0.0003 | 0.0406 | Downregulated |
| hsa-miR-103a-3p | 21.18 | 9.39 | 0.0005 | 0.0406 | Downregulated |
| hsa-miR-141-3p | 13.30 | 3.76 | 0.0004 | 0.0406 | Downregulated |
| hsa-miR-15b-5p | 9.35 | 1.64 | <.0001 | 0.0406 | Downregulated |
| hsa-miR-16-5p | 16.07 | -0.96 | 0.0004 | 0.0406 | Downregulated |
| hsa-miR-194-5p | -20.67 | -42.84 | 0.0004 | 0.0406 | Downregulated |
| hsa-miR-200c-3p | 36.61 | 6.04 | 0.0003 | 0.0406 | Downregulated |
| hsa-miR-215 | -20.96 | -33.34 | 0.0004 | 0.0406 | Downregulated |
| hsa-miR-30b-5p | 2.26 | -2.26 | 0.0004 | 0.0406 | Downregulated |
| hsa-miR-92a-3p | 62.24 | 45.02 | 0.0005 | 0.0406 | Downregulated |
| hsa-miR-192-5p | -32.40 | -59.23 | 0.0007 | 0.0474 | Downregulated |
| hsa-miR-200a-3p | 5.36 | 2.30 | 0.0007 | 0.0474 | Downregulated |
| hsa-miR-107 | 13.80 | 6.52 | 0.0009 | 0.0488 | Downregulated |
| hsa-miR-20a-5p | 43.71 | 33.81 | 0.0009 | 0.0488 | Downregulated |
| hsa-miR-29a-3p | 58.54 | 37.06 | 0.0008 | 0.0488 | Downregulated |
| hsa-miR-200b-3p | 33.13 | 3.61 | 0.0011 | 0.0497 | Downregulated |
| hsa-miR-27a-3p | 31.16 | 23.35 | 0.0010 | 0.0497 | Downregulated |
| hsa-miR-27b-3p | 8.79 | 3.34 | 0.0011 | 0.0497 | Downregulated |
| hsa-miR-4482-3p | 0.53 | 1.71 | 0.0013 | 0.0556 | Upregulated |
| hsa-miR-26a-5p | 12.73 | -7.66 | 0.0015 | 0.0610 | Downregulated |
| hsa-let-7i-5p | 21.68 | 12.54 | 0.0016 | 0.0619 | Downregulated |
| hsa-let-7f-5p | 51.14 | 21.27 | 0.0018 | 0.0636 | Downregulated |
| hsa-miR-23b-3p | 5.74 | -4.69 | 0.0018 | 0.0636 | Downregulated |
| hsa-miR-20b-5p | 10.98 | 8.41 | 0.0020 | 0.0677 | Downregulated |
| hsa-miR-17-5p | 36.45 | 29.31 | 0.0021 | 0.0683 | Downregulated |
| hsa-let-7b-5p | 74.76 | 17.19 | 0.0025 | 0.0729 | Downregulated |
| hsa-let-7d-5p | 15.35 | 8.41 | 0.0026 | 0.0729 | Downregulated |
| hsa-miR-30c-5p | -1.77 | -3.58 | 0.0026 | 0.0729 | Downregulated |
| hsa-miR-3651 | 31.43 | 23.36 | 0.0025 | 0.0729 | Downregulated |
| hsa-miR-23a-3p | 85.18 | 60.36 | 0.0029 | 0.0786 | Downregulated |
| hsa-let-7a-5p | 79.78 | 26.67 | 0.0030 | 0.0787 | Downregulated |
| hsa-miR-30b-3p | 0.43 | 1.08 | 0.0031 | 0.0788 | Upregulated |
| hsa-miR-378f | 0.31 | 1.04 | 0.0036 | 0.0887 | Upregulated |

| Supplemental Table 5. TGFBeta Pathway SNPs associated with miRNAs in differential rectal tissue; FDR<0.09 | | | | | |
| --- | --- | --- | --- | --- | --- |
| miRNA | Mean | Mean | P-values | FDR adjusted P | Direction |
| ***EIF4E* (rs11727086)** | AA ( N=268 ) | AG/GG ( N=193 ) |  |  |  |
| hsa-miR-3189-3p | 0.42 | 1.45 | 0.0002 | 0.0825 | Upregulated |
| hsa-miR-5700 | -0.08 | 0.39 | 0.0002 | 0.0825 | Upregulated |
| ***SMAD3* (rs12901071)** | AA/AG ( N=401 ) | GG ( N=35 ) |  |  |  |
| hsa-miR-106b-5p | 8.99 | 10.77 | 0.0062 | 0.0633 | Downregulated |
| hsa-miR-1202 | -87.93 | -307.26 | 0.0050 | 0.0633 | Downregulated |
| hsa-miR-1207-5p | -271.79 | -642.49 | 0.0037 | 0.0633 | Downregulated |
| hsa-miR-1224-5p | -108.00 | -243.68 | 0.0038 | 0.0633 | Downregulated |
| hsa-miR-1226-5p | -3.96 | -12.74 | 0.0049 | 0.0633 | Downregulated |
| hsa-miR-1229-3p | -7.12 | -11.15 | 0.0021 | 0.0633 | Downregulated |
| hsa-miR-1233-1-5p | -26.35 | -52.61 | 0.0061 | 0.0633 | Downregulated |
| hsa-miR-1249 | -1.87 | -8.53 | 0.0023 | 0.0633 | Downregulated |
| hsa-miR-1273c | -0.78 | -5.34 | 0.0020 | 0.0633 | Downregulated |
| hsa-miR-134 | -35.39 | -87.31 | 0.0011 | 0.0633 | Downregulated |
| hsa-miR-149-3p | -2.88 | -7.98 | 0.0046 | 0.0633 | Downregulated |
| hsa-miR-17-5p | 40.01 | 46.53 | 0.0030 | 0.0633 | Upregulated |
| hsa-miR-188-5p | -52.14 | -138.57 | 0.0026 | 0.0633 | Downregulated |
| hsa-miR-197-5p | -238.29 | -716.04 | 0.0036 | 0.0633 | Downregulated |
| hsa-miR-19b-3p | 17.46 | 20.65 | 0.0024 | 0.0633 | Downregulated |
| hsa-miR-20b-5p | 12.23 | 14.48 | 0.0018 | 0.0633 | Upregulated |
| hsa-miR-26b-5p | 0.16 | 2.92 | 0.0037 | 0.0633 | Downregulated |
| hsa-miR-2861 | -1175.18 | -2275.40 | 0.0065 | 0.0633 | Downregulated |
| hsa-miR-29b-3p | 11.43 | 16.18 | 0.0002 | 0.0633 | Upregulated |
| hsa-miR-29c-3p | 1.32 | 5.20 | 0.0017 | 0.0633 | Downregulated |
| hsa-miR-3132 | -0.42 | -4.66 | 0.0022 | 0.0633 | Downregulated |
| hsa-miR-3188 | -35.57 | -68.70 | 0.0059 | 0.0633 | Downregulated |
| hsa-miR-3194-5p | -15.89 | -43.87 | 0.0063 | 0.0633 | Downregulated |
| hsa-miR-3196 | -223.46 | -396.40 | 0.0047 | 0.0633 | Downregulated |
| hsa-miR-3197 | -3.13 | -7.12 | 0.0055 | 0.0633 | Downregulated |
| hsa-miR-34a-5p | 8.57 | 11.04 | 0.0012 | 0.0633 | Upregulated |
| hsa-miR-3610 | -20.83 | -45.81 | 0.0049 | 0.0633 | Downregulated |
| hsa-miR-3621 | -3.32 | -8.78 | 0.0038 | 0.0633 | Downregulated |
| hsa-miR-3665 | -533.04 | -1101.36 | 0.0066 | 0.0633 | Downregulated |
| hsa-miR-3682-3p | -1.62 | -12.28 | 0.0063 | 0.0633 | Downregulated |
| hsa-miR-370 | 2.70 | -4.18 | 0.0021 | 0.0633 | Downregulated |
| hsa-miR-371a-5p | -7.65 | -39.77 | 0.0021 | 0.0633 | Downregulated |
| hsa-miR-3917 | -3.25 | -18.21 | 0.0045 | 0.0633 | Downregulated |
| hsa-miR-3925-5p | -4.64 | -10.52 | 0.0064 | 0.0633 | Downregulated |
| hsa-miR-3934-3p | -2.65 | -6.87 | 0.0007 | 0.0633 | Downregulated |
| hsa-miR-3945 | -7.76 | -14.21 | 0.0056 | 0.0633 | Downregulated |
| hsa-miR-4253 | -2.64 | -9.12 | 0.0009 | 0.0633 | Downregulated |
| hsa-miR-4270 | -20.58 | -94.56 | 0.0042 | 0.0633 | Downregulated |
| hsa-miR-4314 | -3.87 | -10.44 | 0.0057 | 0.0633 | Downregulated |
| hsa-miR-4419b | -1.11 | -4.38 | 0.0041 | 0.0633 | Downregulated |
| hsa-miR-4430 | -168.61 | -399.75 | 0.0021 | 0.0633 | Downregulated |
| hsa-miR-4476 | -6.62 | -16.39 | 0.0066 | 0.0633 | Downregulated |
| hsa-miR-4481 | -4.73 | -16.10 | 0.0051 | 0.0633 | Downregulated |
| hsa-miR-4486 | -14.98 | -50.49 | 0.0017 | 0.0633 | Downregulated |
| hsa-miR-4487 | -9.85 | -25.84 | 0.0030 | 0.0633 | Downregulated |
| hsa-miR-4497 | -404.65 | -1071.14 | 0.0006 | 0.0633 | Downregulated |
| hsa-miR-4508 | -11.27 | -19.57 | 0.0058 | 0.0633 | Downregulated |
| hsa-miR-4513 | 0.07 | -3.34 | 0.0057 | 0.0633 | Downregulated |
| hsa-miR-4516 | -3221.62 | -6327.76 | 0.0031 | 0.0633 | Downregulated |
| hsa-miR-4530 | -582.44 | -2083.04 | 0.0007 | 0.0633 | Downregulated |
| hsa-miR-4535 | -5.24 | -13.70 | 0.0043 | 0.0633 | Downregulated |
| hsa-miR-4632-5p | -18.74 | -47.70 | 0.0037 | 0.0633 | Downregulated |
| hsa-miR-4646-5p | -2.94 | -19.35 | 0.0042 | 0.0633 | Downregulated |
| hsa-miR-4655-5p | -0.88 | -6.36 | 0.0013 | 0.0633 | Downregulated |
| hsa-miR-4656 | -2.80 | -21.20 | 0.0044 | 0.0633 | Downregulated |
| hsa-miR-4665-5p | -3.35 | -10.95 | 0.0030 | 0.0633 | Downregulated |
| hsa-miR-4695-5p | -40.01 | -97.81 | 0.0015 | 0.0633 | Downregulated |
| hsa-miR-4721 | -158.17 | -394.68 | 0.0031 | 0.0633 | Downregulated |
| hsa-miR-4734 | -26.33 | -64.44 | 0.0019 | 0.0633 | Downregulated |
| hsa-miR-4739 | -150.29 | -326.46 | 0.0008 | 0.0633 | Downregulated |
| hsa-miR-4740-5p | -1.84 | -7.04 | 0.0022 | 0.0633 | Downregulated |
| hsa-miR-4741 | -61.75 | -305.13 | 0.0028 | 0.0633 | Downregulated |
| hsa-miR-4745-5p | -36.68 | -76.35 | 0.0024 | 0.0633 | Downregulated |
| hsa-miR-4758-5p | 2.72 | -16.93 | 0.0061 | 0.0633 | Downregulated |
| hsa-miR-4763-3p | -115.00 | -347.06 | 0.0055 | 0.0633 | Downregulated |
| hsa-miR-4783-3p | -2.21 | -5.03 | 0.0023 | 0.0633 | Downregulated |
| hsa-miR-4788 | -15.89 | -64.61 | 0.0036 | 0.0633 | Downregulated |
| hsa-miR-5001-5p | -220.39 | -447.83 | 0.0063 | 0.0633 | Downregulated |
| hsa-miR-5195-3p | -13.20 | -40.01 | 0.0049 | 0.0633 | Downregulated |
| hsa-miR-550b-2-5p | -3.60 | -8.67 | 0.0011 | 0.0633 | Downregulated |
| hsa-miR-557 | -2.46 | -14.88 | 0.0053 | 0.0633 | Downregulated |
| hsa-miR-5739 | -70.96 | -284.36 | 0.0052 | 0.0633 | Downregulated |
| hsa-miR-575 | -54.94 | -159.21 | 0.0020 | 0.0633 | Downregulated |
| hsa-miR-601 | -4.27 | -11.50 | 0.0025 | 0.0633 | Downregulated |
| hsa-miR-6068 | -493.94 | -937.29 | 0.0054 | 0.0633 | Downregulated |
| hsa-miR-6085 | -43.72 | -147.12 | 0.0065 | 0.0633 | Downregulated |
| hsa-miR-617 | -7.94 | -15.85 | 0.0049 | 0.0633 | Downregulated |
| hsa-miR-638 | -698.81 | -1357.28 | 0.0061 | 0.0633 | Downregulated |
| hsa-miR-6511b-5p | -1.58 | -13.01 | 0.0041 | 0.0633 | Downregulated |
| hsa-miR-718 | -15.39 | -32.74 | 0.0025 | 0.0633 | Downregulated |
| hsa-miR-760 | -0.80 | -6.16 | 0.0059 | 0.0633 | Downregulated |
| hsa-miR-762 | -358.56 | -665.57 | 0.0046 | 0.0633 | Downregulated |
| hsa-miR-769-3p | -3.77 | -6.56 | 0.0048 | 0.0633 | Downregulated |
| hsa-miR-877-5p | -0.59 | -5.39 | 0.0048 | 0.0633 | Downregulated |
| hsa-miR-887 | -10.39 | -17.90 | 0.0018 | 0.0633 | Downregulated |
| hsa-miR-99b-3p | -5.92 | -13.85 | 0.0058 | 0.0633 | Downregulated |
| hsa-miR-125a-3p | -22.67 | -54.14 | 0.0068 | 0.0637 | Downregulated |
| hsa-miR-6125 | -2115.61 | -3916.96 | 0.0068 | 0.0637 | Downregulated |
| hsa-miR-1185-2-3p | -9.33 | -28.89 | 0.0083 | 0.0639 | Downregulated |
| hsa-miR-1225-5p | -353.88 | -781.66 | 0.0089 | 0.0639 | Downregulated |
| hsa-miR-1229-5p | -67.63 | -171.09 | 0.0094 | 0.0639 | Downregulated |
| hsa-miR-1234-5p | -783.10 | -1601.53 | 0.0069 | 0.0639 | Downregulated |
| hsa-miR-1307-5p | -13.73 | -24.12 | 0.0083 | 0.0639 | Downregulated |
| hsa-miR-135a-3p | -19.97 | -43.41 | 0.0083 | 0.0639 | Downregulated |
| hsa-miR-1915-3p | -577.21 | -1170.78 | 0.0090 | 0.0639 | Downregulated |
| hsa-miR-3162-5p | -302.14 | -762.70 | 0.0073 | 0.0639 | Downregulated |
| hsa-miR-345-5p | -4.79 | -11.67 | 0.0088 | 0.0639 | Downregulated |
| hsa-miR-3622a-5p | -5.19 | -10.25 | 0.0096 | 0.0639 | Downregulated |
| hsa-miR-3656 | -302.97 | -896.25 | 0.0088 | 0.0639 | Downregulated |
| hsa-miR-3937 | -4.48 | -14.81 | 0.0075 | 0.0639 | Downregulated |
| hsa-miR-3960 | -1355.00 | -3407.81 | 0.0092 | 0.0639 | Downregulated |
| hsa-miR-423-5p | -5.93 | -12.85 | 0.0090 | 0.0639 | Downregulated |
| hsa-miR-4271 | -16.45 | -57.06 | 0.0076 | 0.0639 | Downregulated |
| hsa-miR-4499 | -66.90 | -145.62 | 0.0079 | 0.0639 | Downregulated |
| hsa-miR-4532 | -73.72 | -163.47 | 0.0072 | 0.0639 | Downregulated |
| hsa-miR-4651 | -16.96 | -49.92 | 0.0081 | 0.0639 | Downregulated |
| hsa-miR-4664-3p | -6.40 | -11.24 | 0.0096 | 0.0639 | Downregulated |
| hsa-miR-4687-3p | -331.87 | -647.67 | 0.0092 | 0.0639 | Downregulated |
| hsa-miR-4787-5p | -317.29 | -552.51 | 0.0076 | 0.0639 | Downregulated |
| hsa-miR-5003-3p | -1.37 | -4.84 | 0.0087 | 0.0639 | Downregulated |
| hsa-miR-5006-5p | -97.97 | -170.89 | 0.0083 | 0.0639 | Downregulated |
| hsa-miR-513c-5p | -7.53 | -17.93 | 0.0096 | 0.0639 | Downregulated |
| hsa-miR-516a-5p | -3.23 | -7.83 | 0.0094 | 0.0639 | Downregulated |
| hsa-miR-5189 | -1.78 | -5.78 | 0.0094 | 0.0639 | Downregulated |
| hsa-miR-564 | 0.10 | -6.96 | 0.0073 | 0.0639 | Downregulated |
| hsa-miR-6087 | -2894.51 | -5484.66 | 0.0085 | 0.0639 | Downregulated |
| hsa-miR-6089 | -5792.69 | -11436.68 | 0.0079 | 0.0639 | Downregulated |
| hsa-miR-6127 | -131.93 | -347.80 | 0.0084 | 0.0639 | Downregulated |
| hsa-miR-623 | -4.34 | -11.10 | 0.0077 | 0.0639 | Downregulated |
| hsa-miR-6511a-5p | 1.54 | -4.32 | 0.0088 | 0.0639 | Downregulated |
| hsa-miR-6722-3p | -8.90 | -23.71 | 0.0073 | 0.0639 | Downregulated |
| hsa-miR-6723-5p | -8.99 | -25.27 | 0.0075 | 0.0639 | Downregulated |
| hsa-miR-874 | -30.99 | -57.72 | 0.0086 | 0.0639 | Downregulated |
| hsa-miR-937-5p | -40.63 | -153.78 | 0.0074 | 0.0639 | Downregulated |
| hsa-miR-4538 | -40.04 | -58.24 | 0.0097 | 0.0640 | Downregulated |
| hsa-miR-4634 | -55.69 | -112.53 | 0.0099 | 0.0648 | Downregulated |
| hsa-miR-1470 | -1.20 | -0.62 | 0.0101 | 0.0651 | Upregulated |
| hsa-miR-345-3p | 0.35 | -8.64 | 0.0101 | 0.0651 | Downregulated |
| hsa-miR-572 | -97.52 | -169.04 | 0.0104 | 0.0665 | Downregulated |
| hsa-miR-6076 | -19.46 | -50.54 | 0.0106 | 0.0673 | Downregulated |
| hsa-miR-1185-1-3p | -42.98 | -93.11 | 0.0115 | 0.0679 | Downregulated |
| hsa-miR-1307-3p | -2.00 | -3.75 | 0.0116 | 0.0679 | Downregulated |
| hsa-miR-1469 | -5.60 | -11.60 | 0.0111 | 0.0679 | Downregulated |
| hsa-miR-150-3p | -21.50 | -53.88 | 0.0111 | 0.0679 | Downregulated |
| hsa-miR-20a-5p | 47.45 | 53.82 | 0.0114 | 0.0679 | Downregulated |
| hsa-miR-30c-1-3p | -1.75 | -3.02 | 0.0110 | 0.0679 | Downregulated |
| hsa-miR-3156-5p | -6.48 | -17.30 | 0.0112 | 0.0679 | Downregulated |
| hsa-miR-3663-3p | -19.24 | -41.53 | 0.0116 | 0.0679 | Downregulated |
| hsa-miR-4515 | -53.54 | -97.25 | 0.0114 | 0.0679 | Downregulated |
| hsa-miR-4784 | -1.41 | -3.71 | 0.0112 | 0.0679 | Downregulated |
| hsa-miR-6086 | -37.07 | -93.41 | 0.0108 | 0.0679 | Downregulated |
| hsa-miR-4463 | -14.40 | -52.81 | 0.0117 | 0.0680 | Downregulated |
| hsa-miR-1236-5p | -6.08 | -26.53 | 0.0119 | 0.0682 | Downregulated |
| hsa-miR-671-5p | -31.00 | -69.98 | 0.0119 | 0.0682 | Downregulated |
| hsa-miR-4433-5p | -1.30 | -5.21 | 0.0120 | 0.0683 | Downregulated |
| hsa-miR-1227-5p | -169.95 | -314.35 | 0.0122 | 0.0689 | Downregulated |
| hsa-miR-373-5p | -0.98 | -3.46 | 0.0126 | 0.0693 | Downregulated |
| hsa-miR-4746-3p | -52.52 | -107.17 | 0.0126 | 0.0693 | Downregulated |
| hsa-miR-5088 | -7.76 | -15.12 | 0.0126 | 0.0693 | Downregulated |
| hsa-miR-939-5p | -76.51 | -176.01 | 0.0124 | 0.0693 | Downregulated |
| hsa-miR-550a-3-5p | -0.74 | -3.60 | 0.0127 | 0.0694 | Downregulated |
| hsa-miR-1273g-5p | -0.38 | 1.12 | 0.0129 | 0.0700 | Upregulated |
| hsa-miR-3940-5p | -114.05 | -237.00 | 0.0130 | 0.0701 | Downregulated |
| hsa-miR-10a-5p | 15.51 | 20.86 | 0.0134 | 0.0709 | Downregulated |
| hsa-miR-4327 | 3.44 | -30.21 | 0.0134 | 0.0709 | Downregulated |
| hsa-miR-4534 | -38.01 | -68.99 | 0.0133 | 0.0709 | Downregulated |
| hsa-miR-639 | -1.00 | -2.35 | 0.0136 | 0.0715 | Downregulated |
| hsa-miR-3138 | -7.10 | -16.65 | 0.0138 | 0.0720 | Downregulated |
| hsa-miR-198 | -5.20 | -13.06 | 0.0140 | 0.0722 | Downregulated |
| hsa-miR-6126 | -144.15 | -234.99 | 0.0140 | 0.0722 | Downregulated |
| hsa-miR-4776-5p | -0.93 | -4.18 | 0.0146 | 0.0743 | Downregulated |
| hsa-miR-5190 | -0.76 | -3.68 | 0.0145 | 0.0743 | Downregulated |
| hsa-miR-3622b-5p | 0.38 | -3.68 | 0.0147 | 0.0744 | Downregulated |
| hsa-miR-1183 | -1.56 | -8.41 | 0.0150 | 0.0745 | Downregulated |
| hsa-miR-1299 | -2.15 | -6.02 | 0.0149 | 0.0745 | Downregulated |
| hsa-miR-4701-3p | -0.63 | -13.47 | 0.0150 | 0.0745 | Downregulated |
| hsa-miR-15b-5p | 8.95 | 11.45 | 0.0152 | 0.0746 | Downregulated |
| hsa-miR-4689 | 0.28 | -17.82 | 0.0152 | 0.0746 | Downregulated |
| hsa-miR-1587 | -132.35 | -361.64 | 0.0154 | 0.0747 | Downregulated |
| hsa-miR-298 | -4.64 | -7.15 | 0.0154 | 0.0747 | Downregulated |
| hsa-miR-4496 | -3.68 | -11.49 | 0.0156 | 0.0748 | Downregulated |
| hsa-miR-4738-3p | -1.38 | -6.45 | 0.0156 | 0.0748 | Downregulated |
| hsa-miR-4442 | -9.39 | -43.83 | 0.0159 | 0.0758 | Downregulated |
| hsa-miR-1228-3p | -2.88 | -9.32 | 0.0162 | 0.0768 | Downregulated |
| hsa-miR-199a-3p | 21.72 | 22.53 | 0.0172 | 0.0811 | Downregulated |
| hsa-miR-3180-5p | 0.00 | 0.85 | 0.0174 | 0.0816 | Upregulated |
| hsa-miR-361-5p | 5.93 | 8.16 | 0.0175 | 0.0816 | Upregulated |
| hsa-miR-4257 | -41.13 | -91.62 | 0.0176 | 0.0816 | Downregulated |
| hsa-miR-4429 | -0.52 | -3.39 | 0.0177 | 0.0816 | Downregulated |
| hsa-miR-10b-3p | -5.57 | -11.54 | 0.0181 | 0.0820 | Downregulated |
| hsa-miR-1275 | -126.82 | -225.99 | 0.0181 | 0.0820 | Downregulated |
| hsa-miR-371b-5p | -322.89 | -547.17 | 0.0181 | 0.0820 | Downregulated |
| hsa-miR-3158-5p | -2.81 | -7.00 | 0.0188 | 0.0843 | Downregulated |
| hsa-miR-4466 | -268.95 | -556.69 | 0.0187 | 0.0843 | Downregulated |
| hsa-miR-6075 | -42.43 | -75.71 | 0.0196 | 0.0874 | Downregulated |
| hsa-miR-4733-5p | -2.54 | -11.12 | 0.0204 | 0.0886 | Downregulated |
| hsa-miR-4787-3p | -18.09 | -30.89 | 0.0202 | 0.0886 | Downregulated |
| hsa-miR-602 | -3.48 | -8.56 | 0.0200 | 0.0886 | Downregulated |
| hsa-miR-610 | -0.78 | -3.64 | 0.0202 | 0.0886 | Downregulated |
| hsa-miR-662 | -5.46 | -10.15 | 0.0203 | 0.0886 | Downregulated |
| hsa-miR-6088 | -373.88 | -842.76 | 0.0207 | 0.0894 | Downregulated |
| ***SMAD3* (rs1498506)** | AA ( N=120 ) | AC/CC ( N=316 ) |  |  |  |
| hsa-miR-652-3p | 0.05 | 0.63 | <.0001 | 0.0825 | Upregulated |
| ***BMPR2* (rs2228545)** | GG ( N=403 ) | GA/AA ( N=33 ) |  |  |  |
| hsa-miR-324-5p | 1.42 | 3.90 | 0.0002 | 0.0825 | Upregulated |
| hsa-miR-484 | 0.06 | 1.57 | <.0001 | 0.0825 | Upregulated |
| ***NFKB1* (rs230510)** | AA ( N=166 ) | AT/TT ( N=270 ) |  |  |  |
| hsa-miR-1276 | 0.59 | -0.02 | 0.0013 | 0.0975 | Downregulated |
| hsa-miR-3616-3p | 1.42 | 0.25 | 0.0006 | 0.0722 | Downregulated |
| hsa-miR-3617-5p | 0.74 | -0.31 | 0.0005 | 0.0722 | Downregulated |
| hsa-miR-4458 | -0.90 | -2.24 | 0.0002 | 0.0722 | Downregulated |
| hsa-miR-4518 | 0.23 | -0.49 | 0.0007 | 0.0722 | Downregulated |
| hsa-miR-4684-3p | 0.38 | -0.27 | 0.0003 | 0.0722 | Downregulated |
| hsa-miR-4768-3p | 0.43 | -0.49 | 0.0007 | 0.0722 | Downregulated |
| hsa-miR-493-3p | 0.86 | 0.14 | 0.0005 | 0.0722 | Downregulated |
| hsa-miR-6720-3p | 0.55 | -0.66 | 0.0007 | 0.0722 | Downregulated |
| ***SMAD3* (rs2414937)** | GG/GC ( N=418 ) | CC ( N=18 ) |  |  |  |
| hsa-miR-1273g-3p | -791.41 | -65.42 | 0.0006 | 0.0550 | Upregulated |
| hsa-miR-17-5p | 41.32 | 22.24 | 0.0003 | 0.0550 | Downregulated |
| hsa-miR-196b-5p | 11.40 | 2.67 | 0.0003 | 0.0550 | Downregulated |
| hsa-miR-20a-5p | 48.80 | 28.38 | 0.0005 | 0.0550 | Downregulated |
| hsa-miR-3651 | 34.71 | 18.55 | 0.0006 | 0.0550 | Downregulated |
| hsa-miR-375 | -46.53 | -24.12 | 0.0005 | 0.0550 | Upregulated |
| hsa-miR-4538 | -42.83 | -10.65 | 0.0004 | 0.0550 | Upregulated |
| hsa-miR-4539 | -23.96 | -5.29 | <.0001 | 0.0550 | Upregulated |
| hsa-miR-4697-5p | -19.48 | 13.48 | 0.0003 | 0.0550 | Upregulated |
| hsa-miR-30c-1-3p | -1.96 | 0.68 | 0.0007 | 0.0577 | Upregulated |
| hsa-miR-1229-3p | -7.77 | 0.12 | 0.0008 | 0.0600 | Upregulated |
| hsa-miR-3945 | -8.78 | 3.37 | 0.0009 | 0.0619 | Upregulated |
| hsa-miR-24-3p | 41.16 | 14.59 | 0.0011 | 0.0648 | Downregulated |
| hsa-miR-4690-5p | -49.58 | 8.56 | 0.0011 | 0.0648 | Upregulated |
| hsa-miR-1471 | -38.71 | 41.18 | 0.0013 | 0.0660 | Upregulated |
| hsa-miR-21-5p | 269.70 | 134.22 | 0.0016 | 0.0660 | Upregulated |
| hsa-miR-4459 | -6473.12 | 2453.53 | 0.0016 | 0.0660 | Upregulated |
| hsa-miR-5006-5p | -109.74 | 33.63 | 0.0012 | 0.0660 | Upregulated |
| hsa-miR-718 | -18.10 | 13.88 | 0.0015 | 0.0660 | Upregulated |
| hsa-miR-93-5p | 24.24 | 12.84 | 0.0015 | 0.0660 | Downregulated |
| hsa-miR-19b-3p | 18.06 | 9.70 | 0.0021 | 0.0825 | Downregulated |
| hsa-miR-23a-3p | 87.54 | 43.56 | 0.0023 | 0.0862 | Downregulated |
| hsa-miR-29b-3p | 12.13 | 4.44 | 0.0028 | 0.0888 | Upregulated |
| hsa-miR-4470 | -7.09 | -0.66 | 0.0027 | 0.0888 | Upregulated |
| hsa-miR-4476 | -8.14 | 9.59 | 0.0028 | 0.0888 | Upregulated |
| hsa-miR-6083 | -7.59 | -0.61 | 0.0025 | 0.0888 | Upregulated |
| ***RUNX2* (rs2819854)** | CC/CT ( N=341 ) | TT ( N=95 ) |  |  |  |
| hsa-miR-10b-5p | -2.34 | 1.92 | 0.0012 | 0.0715 | Upregulated |
| hsa-miR-1182 | 1.20 | -1.13 | 0.0008 | 0.0715 | Downregulated |
| hsa-miR-1281 | -9.98 | -17.90 | 0.0013 | 0.0715 | Downregulated |
| hsa-miR-129-5p | -1.16 | -2.30 | 0.0008 | 0.0715 | Downregulated |
| hsa-miR-3150b-5p | -3.63 | -6.53 | 0.0013 | 0.0715 | Upregulated |
| hsa-miR-3648 | 45.76 | 9.95 | 0.0009 | 0.0715 | Downregulated |
| hsa-miR-365a-5p | -0.28 | -2.66 | 0.0006 | 0.0715 | Downregulated |
| hsa-miR-3911 | 10.28 | 1.46 | 0.0008 | 0.0715 | Downregulated |
| hsa-miR-4665-3p | -20.84 | -40.12 | 0.0012 | 0.0715 | Downregulated |
| hsa-miR-4767 | -2.92 | -7.01 | 0.0009 | 0.0715 | Downregulated |
| hsa-miR-4769-3p | -2.33 | -4.60 | 0.0009 | 0.0715 | Downregulated |
| hsa-miR-514b-5p | 2.08 | -1.03 | 0.0013 | 0.0715 | Downregulated |
| hsa-miR-5585-3p | 5.26 | -36.48 | <.0001 | 0.0715 | Downregulated |
| hsa-miR-663a | 123.56 | 81.47 | 0.0013 | 0.0715 | Downregulated |
| hsa-miR-940 | -180.62 | -295.99 | 0.0013 | 0.0715 | Downregulated |
| hsa-miR-1181 | -13.62 | -32.79 | 0.0069 | 0.0776 | Downregulated |
| hsa-miR-1185-1-3p | -38.97 | -75.87 | 0.0053 | 0.0776 | Downregulated |
| hsa-miR-1185-2-3p | -8.19 | -20.62 | 0.0045 | 0.0776 | Downregulated |
| hsa-miR-1225-3p | -2.74 | -5.73 | 0.0016 | 0.0776 | Downregulated |
| hsa-miR-1228-3p | -2.37 | -7.10 | 0.0038 | 0.0776 | Downregulated |
| hsa-miR-1234-3p | -12.45 | -18.77 | 0.0052 | 0.0776 | Downregulated |
| hsa-miR-1234-5p | -740.56 | -1237.35 | 0.0066 | 0.0776 | Downregulated |
| hsa-miR-1249 | -1.69 | -4.99 | 0.0077 | 0.0776 | Downregulated |
| hsa-miR-1258 | -0.67 | 0.13 | 0.0049 | 0.0776 | Upregulated |
| hsa-miR-1268b | -25.27 | -149.47 | 0.0025 | 0.0776 | Downregulated |
| hsa-miR-1273d | -0.41 | -2.45 | 0.0052 | 0.0776 | Downregulated |
| hsa-miR-1273f | -17.00 | -43.29 | 0.0016 | 0.0776 | Downregulated |
| hsa-miR-1285-3p | -0.93 | -2.63 | 0.0017 | 0.0776 | Downregulated |
| hsa-miR-1471 | -28.69 | -59.53 | 0.0052 | 0.0776 | Downregulated |
| hsa-miR-150-3p | -18.98 | -42.49 | 0.0042 | 0.0776 | Downregulated |
| hsa-miR-1587 | -118.31 | -267.24 | 0.0043 | 0.0776 | Downregulated |
| hsa-miR-1972 | -3.30 | -12.25 | 0.0035 | 0.0776 | Downregulated |
| hsa-miR-2467-3p | 1.77 | 0.65 | 0.0054 | 0.0776 | Downregulated |
| hsa-miR-28-3p | -0.05 | 0.41 | 0.0048 | 0.0776 | Upregulated |
| hsa-miR-29c-3p | 0.93 | 4.13 | 0.0079 | 0.0776 | Upregulated |
| hsa-miR-3135b | -50.06 | -98.00 | 0.0059 | 0.0776 | Downregulated |
| hsa-miR-3141 | 14.59 | -5.76 | 0.0041 | 0.0776 | Downregulated |
| hsa-miR-3147 | 1.70 | -1.14 | 0.0077 | 0.0776 | Downregulated |
| hsa-miR-3202 | -0.35 | -2.55 | 0.0060 | 0.0776 | Downregulated |
| hsa-miR-345-5p | -4.28 | -9.15 | 0.0051 | 0.0776 | Downregulated |
| hsa-miR-371a-5p | -6.12 | -24.99 | 0.0049 | 0.0776 | Downregulated |
| hsa-miR-378b | 0.28 | -1.41 | 0.0076 | 0.0776 | Downregulated |
| hsa-miR-3926 | 0.32 | -2.15 | 0.0051 | 0.0776 | Downregulated |
| hsa-miR-4259 | 2.09 | 0.54 | 0.0031 | 0.0776 | Downregulated |
| hsa-miR-4271 | -14.63 | -37.93 | 0.0077 | 0.0776 | Downregulated |
| hsa-miR-4298 | 17.54 | -0.82 | 0.0020 | 0.0776 | Downregulated |
| hsa-miR-4327 | 5.39 | -15.96 | 0.0079 | 0.0776 | Downregulated |
| hsa-miR-4417 | 22.27 | 6.16 | 0.0047 | 0.0776 | Downregulated |
| hsa-miR-4419a | 3.92 | -2.58 | 0.0030 | 0.0776 | Downregulated |
| hsa-miR-4433-3p | -17.18 | -48.70 | 0.0042 | 0.0776 | Downregulated |
| hsa-miR-4433-5p | -0.95 | -4.01 | 0.0019 | 0.0776 | Downregulated |
| hsa-miR-4455 | -4.48 | -21.40 | 0.0028 | 0.0776 | Downregulated |
| hsa-miR-4484 | -4.91 | -11.46 | 0.0071 | 0.0776 | Downregulated |
| hsa-miR-4515 | -50.49 | -80.57 | 0.0076 | 0.0776 | Downregulated |
| hsa-miR-4669 | -21.24 | -53.71 | 0.0078 | 0.0776 | Downregulated |
| hsa-miR-4690-5p | -42.60 | -63.64 | 0.0065 | 0.0776 | Downregulated |
| hsa-miR-4707-3p | -2.88 | -4.48 | 0.0027 | 0.0776 | Downregulated |
| hsa-miR-4710 | 1.49 | -2.35 | 0.0046 | 0.0776 | Downregulated |
| hsa-miR-4728-3p | -1.51 | -3.44 | 0.0033 | 0.0776 | Downregulated |
| hsa-miR-4743-5p | 1.41 | -2.78 | 0.0079 | 0.0776 | Downregulated |
| hsa-miR-4746-3p | -48.28 | -87.89 | 0.0035 | 0.0776 | Downregulated |
| hsa-miR-4749-3p | -6.06 | -7.85 | 0.0068 | 0.0776 | Downregulated |
| hsa-miR-4749-5p | -0.03 | -1.95 | 0.0022 | 0.0776 | Downregulated |
| hsa-miR-4776-5p | -0.66 | -3.10 | 0.0044 | 0.0776 | Downregulated |
| hsa-miR-4787-3p | -16.62 | -28.08 | 0.0074 | 0.0776 | Downregulated |
| hsa-miR-4793-5p | 4.83 | -13.06 | 0.0038 | 0.0776 | Downregulated |
| hsa-miR-4800-5p | 30.27 | 13.28 | 0.0045 | 0.0776 | Downregulated |
| hsa-miR-5008-5p | -0.30 | -4.26 | 0.0045 | 0.0776 | Downregulated |
| hsa-miR-5096 | 0.61 | -3.43 | 0.0024 | 0.0776 | Downregulated |
| hsa-miR-5196-5p | 7.49 | 1.69 | 0.0070 | 0.0776 | Downregulated |
| hsa-miR-550a-3-5p | -0.51 | -2.63 | 0.0052 | 0.0776 | Downregulated |
| hsa-miR-574-5p | -37.85 | -127.46 | 0.0043 | 0.0776 | Downregulated |
| hsa-miR-6069 | -1.06 | -3.85 | 0.0064 | 0.0776 | Downregulated |
| hsa-miR-6075 | -39.93 | -63.66 | 0.0075 | 0.0776 | Downregulated |
| hsa-miR-6085 | -34.29 | -115.63 | 0.0075 | 0.0776 | Downregulated |
| hsa-miR-6086 | -32.91 | -72.77 | 0.0059 | 0.0776 | Downregulated |
| hsa-miR-6088 | -338.71 | -672.85 | 0.0071 | 0.0776 | Downregulated |
| hsa-miR-6126 | -135.07 | -210.22 | 0.0063 | 0.0776 | Downregulated |
| hsa-miR-6165 | 35.19 | 2.62 | 0.0063 | 0.0776 | Downregulated |
| hsa-miR-6508-5p | -0.50 | -2.75 | 0.0038 | 0.0776 | Downregulated |
| hsa-miR-762 | -339.53 | -539.98 | 0.0038 | 0.0776 | Downregulated |
| hsa-miR-877-3p | -7.57 | -12.80 | 0.0019 | 0.0776 | Downregulated |
| hsa-miR-936 | -2.39 | -6.56 | 0.0061 | 0.0776 | Downregulated |
| hsa-miR-937-5p | -37.20 | -94.61 | 0.0069 | 0.0776 | Downregulated |
| hsa-miR-4758-5p | 3.48 | -7.25 | 0.0081 | 0.0786 | Downregulated |
| hsa-miR-602 | -3.15 | -6.54 | 0.0082 | 0.0787 | Downregulated |
| hsa-miR-6515-3p | -0.11 | -1.98 | 0.0083 | 0.0787 | Downregulated |
| hsa-miR-671-5p | -26.96 | -59.90 | 0.0084 | 0.0787 | Downregulated |
| hsa-miR-1224-5p | -104.06 | -172.13 | 0.0089 | 0.0816 | Downregulated |
| hsa-miR-765 | 2.65 | -1.76 | 0.0088 | 0.0816 | Downregulated |
| hsa-miR-6511b-5p | -1.31 | -6.78 | 0.0091 | 0.0825 | Downregulated |
| hsa-miR-3925-5p | -4.42 | -7.61 | 0.0097 | 0.0833 | Downregulated |
| hsa-miR-4257 | -38.60 | -68.80 | 0.0093 | 0.0833 | Downregulated |
| hsa-miR-4281 | -315.92 | -709.18 | 0.0096 | 0.0833 | Downregulated |
| hsa-miR-4534 | -34.20 | -63.12 | 0.0097 | 0.0833 | Downregulated |
| hsa-miR-6129 | -15.39 | -26.28 | 0.0098 | 0.0833 | Downregulated |
| hsa-miR-6724-5p | -69.27 | -149.32 | 0.0096 | 0.0833 | Downregulated |
| hsa-miR-1229-3p | -6.95 | -9.22 | 0.0101 | 0.0841 | Downregulated |
| hsa-miR-3940-5p | -107.71 | -182.13 | 0.0101 | 0.0841 | Downregulated |
| hsa-miR-4738-3p | -0.98 | -4.69 | 0.0102 | 0.0841 | Downregulated |
| hsa-miR-1238-3p | -0.33 | -2.68 | 0.0106 | 0.0849 | Downregulated |
| hsa-miR-3194-5p | -15.76 | -26.69 | 0.0105 | 0.0849 | Downregulated |
| hsa-miR-4707-5p | -6.70 | -17.35 | 0.0104 | 0.0849 | Downregulated |
| ***NFKB1* (rs3821958)** | AA ( N=137 ) | AG/GG ( N=299 ) |  |  |  |
| hsa-miR-3617-5p | -0.71 | 0.45 | <.0001 | 0.0825 | Upregulated |

| Supplemental Table 7. Summary of model used and previously identified associations for colorectal cancer risk and survival. | | | | | | | | | | | |  |  |  |  |
| --- | --- | --- | --- | --- | --- | --- | --- | --- | --- | --- | --- | --- | --- | --- | --- |
|  |  | Colon | | | | | | | Rectal | | | | | | |
|  |  |  | Cancer Risk | | | Survival | | |  | Cancer Risk | | | Survival |  |  |
| Gene | SNP | Model Used | OR | (95% CI) | | HR | (95% CI) | | Model Used | OR | (95% CI) | | HR | (95% CI) |  |
| *MTOR* | rs1057079 | Dom | 1.21 | (1.05, | 1.38) |  |  |  |  |  |  |  |  |  |  |
| *TGFβR1* | rs10733710 | Dom |  |  |  | 0.64 | (0.48, | 0.87) |  |  |  |  |  |  |  |
| *RUNX2* | rs10948238 | Dom |  |  |  | 0.77 | (0.60, | 0.97) |  |  |  |  |  |  |  |
| *SMAD3* | rs11639295 | Rec |  |  |  | 0.42 | (0.11, | 0.85) |  |  |  |  |  |  |  |
| *RUNX1* | rs11701453 |  |  |  |  |  |  |  | Dom | 1.29 | (1.05, | 1.59) |  |  |  |
| *RUNX1* | rs11702779 |  |  |  |  |  |  |  | Rec | 0.68 | (0.49, | 0.93) |  |  |  |
| *NFKB1* | rs11722146 |  |  |  |  |  |  |  | Dom | 1.24 | (1.03, | 1.51) |  |  |  |
| *EIF4E* | rs11727086 | Dom | 1.22 | (1.07, | 1.40) |  |  |  |  |  |  |  |  |  |  |
| *MAPK1* | rs11913721 |  |  |  |  |  |  |  | Rec | 0.74 | (0.96, | 0.56) |  |  |  |
| *BMP1* | rs12114940 |  |  |  |  |  |  |  | Rec |  |  |  | 2.36 | (1.47, | 3.77) |
| *RUNX2* | rs12208240 | Dom | 0.27 | (0.08, | 0.97) |  |  |  |  |  |  |  |  |  |  |
| *RUNX2* | rs12333172 | Rec | 1.52 | (1.08, | 2.13) |  |  |  |  |  |  |  |  |  |  |
| *BMPR2* | rs12477602 | Dom | 0.85 | (0.72, | 1.00) |  |  |  |  |  |  |  |  |  |  |
| *EIF4E* | rs12498533 | Dom |  |  |  | 1.48 | (1.08, | 2.03) |  |  |  |  |  |  |  |
| *BMPR1B* | rs12508087 | Dom |  |  |  | 0.73 | (0.54, | 0.97) |  |  |  |  |  |  |  |
| *SMAD3* | rs12708492 | Dom |  |  |  | 1.69 | (1.21, | 2.37) | Dom |  |  |  | 0.38 | (0.24, | 0.59) |
| *BMPR1A* | rs12765929 | Dom |  |  |  | 1.59 | (1.18, | 2.14) |  |  |  |  |  |  |  |
| *GDF10* | rs12769499 | Rec |  |  |  | 2.41 | (1.03, | 5.62) |  |  |  |  |  |  |  |
| *SMAD3* | rs12901071 | Rec | 0.68 | (0.55, | 0.85) |  |  |  |  |  |  |  |  |  |  |
| *SMAD3* | rs12904944 |  |  |  |  |  |  |  | Dom |  |  |  | 1.43 | (1.00, | 2.03) |
| *SMAD7* | rs12953717 | Dom | 1.36 | (1.12, | 1.65) |  |  |  | Rec |  |  |  | 0.38 | (0.21, | 0.71) |
| *BMPR1B* | rs13134042 | Rec | 0.69 | (0.49, | 0.96) |  |  |  | Dom |  |  |  | 2.07 | (1.42, | 3.02) |
| *RUNX1* | rs1475840 |  |  |  |  |  |  |  | Dom |  |  |  | 0.48 | (0.32, | 0.73) |
| *SMAD3* | rs1498506 | Dom | 0.69 | (0.57, | 0.84) |  |  |  |  |  |  |  |  |  |  |
| *TGFβR1* | rs1571590 | Rec | 1.41 | (1.00, | 1.98) |  |  |  | Dom |  |  |  | 0.64 | (0.43, | 0.95) |
| *SMAD3* | rs16950687 | Dom |  |  |  | 0.73 | (0.55, | 0.97) | Rec |  |  |  | 1.92 | (1.07, | 3.43) |
| *SMAD3* | rs17293443 |  |  |  |  |  |  |  | Rec | 1.81 | (1.12, | 2.91) |  |  |  |
| *BMP4* | rs17563 | Rec | 1.19 | (1.01, | 1.41) | 1.45 | (1.05, | 1.99) |  |  |  |  |  |  |  |
| *BMPR1B* | rs17616243 | Dom | 1.23 | (1.06, | 1.43) |  |  |  |  |  |  |  |  |  |  |
| *SMAD2* | rs1787199 | Dom | 1.25 | (1.03, | 1.51) |  |  |  |  |  |  |  |  |  |  |
| *SMAD2* | rs1792689 |  |  |  |  |  |  |  | Dom | 0.78 | (0.62, | 0.98) |  |  |  |
| *TGFβ1* | rs1800469 | Dom | 0.65 | (0.51, | 0.84) |  |  |  |  |  |  |  |  |  |  |
| *BMPR1B* | rs1863652 | Rec | 0.80 | (0.64, | 0.99) |  |  |  | Rec |  |  |  | 1.95 | (1.12, | 3.40) |
| *RUNX1* | rs1883066 |  |  |  |  |  |  |  | Dom |  |  |  | 0.51 | (0.31, | 0.84) |
| *BMPR1B* | rs2120834 | Rec | 0.77 | (0.63, | 0.94) | 1.52 | (1.03, | 2.26) |  |  |  |  |  |  |  |
| *RUNX3* | rs2135756 |  |  |  |  |  |  |  | Rec |  |  |  | 0.56 | (0.36, | 0.88) |
| *BMPR1A* | rs2168730 |  |  |  |  |  |  |  | Dom |  |  |  | 0.65 | (0.44, | 0.97) |
| *BMPR1B* | rs2214395 | Rec |  |  |  | 2.43 | (1.49, | 3.97) |  |  |  |  |  |  |  |
| *BMPR2* | rs2228545 |  |  |  |  |  |  |  | Dom | 1.92 | (1.27, | 2.89) |  |  |  |
| *RUNX3* | rs2236850 | Dom |  |  |  | 1.48 | (1.13, | 1.94) |  |  |  |  |  |  |  |
| *RUNX1* | rs2242878 | Dom |  |  |  | 1.30 | (1.02, | 1.66) |  |  |  |  |  |  |  |
| *RUNX1* | rs2253319 |  |  |  |  |  |  |  | Dom |  |  |  | 1.68 | (1.13, | 2.51) |
| *RUNX1* | rs2300395 |  |  |  |  |  |  |  | Dom |  |  |  | 0.68 | (0.48, | 0.95) |
| *NFKB1* | rs230510 |  |  |  |  |  |  |  | Dom | 0.79 | (0.51, | 0.94) |  |  |  |
| *SMAD3* | rs2414937 | Rec | 0.67 | (0.47, | 0.97) | 1.51 | (1.14, | 2.01)D |  |  |  |  |  |  |  |
| *EIF4EBP3* | rs250425 | Dom |  |  |  | 1.39 | (1.05, | 1.84) |  |  |  |  |  |  |  |
| *RUNX2* | rs2819854 | Rec | 1.21 | (1.03, | 1.42) |  |  |  |  |  |  |  |  |  |  |
| *RUNX1* | rs2834645 | Dom | 0.83 | (0.72, | 0.96) | 1.33 | (1.04, | 1.70) |  |  |  |  |  |  |  |
| *RUNX1* | rs2834670 | Dom |  |  |  | 0.70 | (0.53, | 0.91) |  |  |  |  |  |  |  |
| *GDF10* | rs2853838 |  |  |  |  |  |  |  | Rec |  |  |  | 0.23 | (0.06, | 0.93) |
| *BMPR1A* | rs2883420 | Dom |  |  |  | 1.47 | (1.09, | 1.99) |  |  |  |  |  |  |  |
| *BMP2* | rs3178250 | Dom |  |  |  | 0.66 | (0.51, | 0.84) | Rec | 1.63 | (1.02, | 2.60) |  |  |  |
| *SMAD7* | rs3736242 | Rec |  |  |  | 1.88 | (1.06, | 3.32) |  |  |  |  |  |  |  |
| *SMAD3* | rs3743343 | Rec |  |  |  | 0.52 | (0.28, | 0.97) |  |  |  |  |  |  |  |
| *NFKB1* | rs3821958 |  |  |  |  |  |  |  | Dom | 1.32 | (1.00, | 1.75) |  |  |  |
| *SMAD3* | rs3825977 |  |  |  |  |  |  |  | Dom |  |  |  | 1.64 | (1.09, | 2.47) |
| *BMP1* | rs3857979 |  |  |  |  |  |  |  | Dom |  |  |  | 1.56 | (1.04, | 2.34) |
| *BMP1* | rs4076873 |  |  |  |  |  |  |  | Dom |  |  |  | 1.93 | (1.32, | 2.83) |
| *NFKB1* | rs4648110 | Rec | 0.66 | (0.45, | 0.96) |  |  |  |  |  |  |  |  |  |  |
| *TGFβ1* | rs4803455 | Dom | 1.43 | (1.18, | 1.73) |  |  |  |  |  |  |  |  |  |  |
| *SMAD7* | rs4939827 | Dom | 0.79 | (0.66, | 0.95) |  |  |  |  |  |  |  |  |  |  |
| *SMAD2* | rs4940086 | Dom | 1.33 | (1.06, | 1.67) |  |  |  |  |  |  |  |  |  |  |
| *PTEN* | rs532678 |  |  |  |  |  |  |  | Dom | 1.25 | (1.02, | 1.52) |  |  |  |
| *TGFβR1* | rs6478974 | Dom | 0.85 | (0.74, | 0.99) |  |  |  | Dom |  |  |  | 1.44 | (1.00 | 2.08) |
| *RUNX3* | rs6672420 | Dom | 0.79 | (0.68, | 0.92) |  |  |  | Rec | 1.29 | (1.03, | 1.62) |  |  |  |
| *BMPR2* | rs6751210 | Rec | 1.23 | (1.05, | 1.46) |  |  |  |  |  |  |  |  |  |  |
| *BMPR1B* | rs6849425 |  |  |  |  |  |  |  | Rec | 0.62 | (0.39, | 0.98) |  |  |  |
| *BMPR1A* | rs7088641 | Dom | 0.84 | (0.73, | 0.96) |  |  |  |  |  |  |  |  |  |  |
| *SMAD3* | rs7163381 | Rec | 0.76 | (0.59, | 0.98) |  |  |  |  |  |  |  |  |  |  |
| *SMAD3* | rs7176870 | Dom |  |  |  | 0.74 | (0.58, | 0.95) |  |  |  |  |  |  |  |
| *SMAD3* | rs7181556 |  |  |  |  |  |  |  | Rec |  |  |  | 2.30 | (1.17, | 4.52) |
| *RUNX1* | rs7279123 | Dom | 1.17 | (1.02, | 1.35) |  |  |  | Dom |  |  |  | 1.48 | (1.06, | 2.08) |
| *RUNX1* | rs7280028 |  |  |  |  |  |  |  | Dom | 0.80 | (0.65, | 0.99) | 3.72 R | (1.54- | 8.96 |
| *RUNX3* | rs7517302 | Dom |  |  |  | 1.50 | (1.09, | 2.06) |  |  |  |  |  |  |  |
| *GDF10* | rs762454 |  |  |  |  |  |  |  | Rec | 1.41 | (1.04, | 1.89) |  |  |  |
| *BMPR1B* | rs7694043 |  |  |  |  |  |  |  | Rec |  |  |  | 2.08 | (1.20, | 3.59) |
| *BMPR1B* | rs7698964 | Rec |  |  |  | 0.20 | (0.06, | 0.68) |  |  |  |  |  |  |  |
| *BMPR1A* | rs7895217 | Dom |  |  |  | 1.30 | (1.02, | 1.66) |  |  |  |  |  |  |  |
| *RUNX1* | rs8134179 | Dom |  |  |  | 0.70 | (0.51, | 0.96) |  |  |  |  |  |  |  |
